# Supplementary material for: Comparative analyses of putative toxin gene homologs from an Old World viper, Daboia russelii
Source: PeerJ. 2017 Dec 5;5:e4104. doi: 10.7717/peerj.4104 (PMC5721910; doi:10.7717/peerj.4104)
Supplement: Supplemental Information 1 [file peerj-05-4104-s001.pdf]

Fig. S1: Agarose gel electrophoresis of genomic DNA (VRU) isolated from shed skin of *Daboia russelii*.

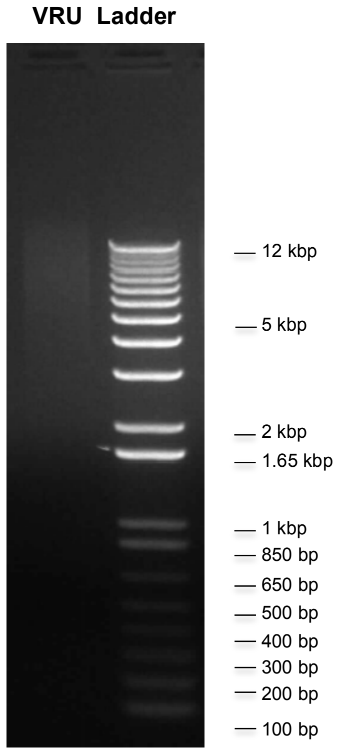

Fig. S2: Exon-intron structures for all the putative toxin gene homologs in Russell's viper.

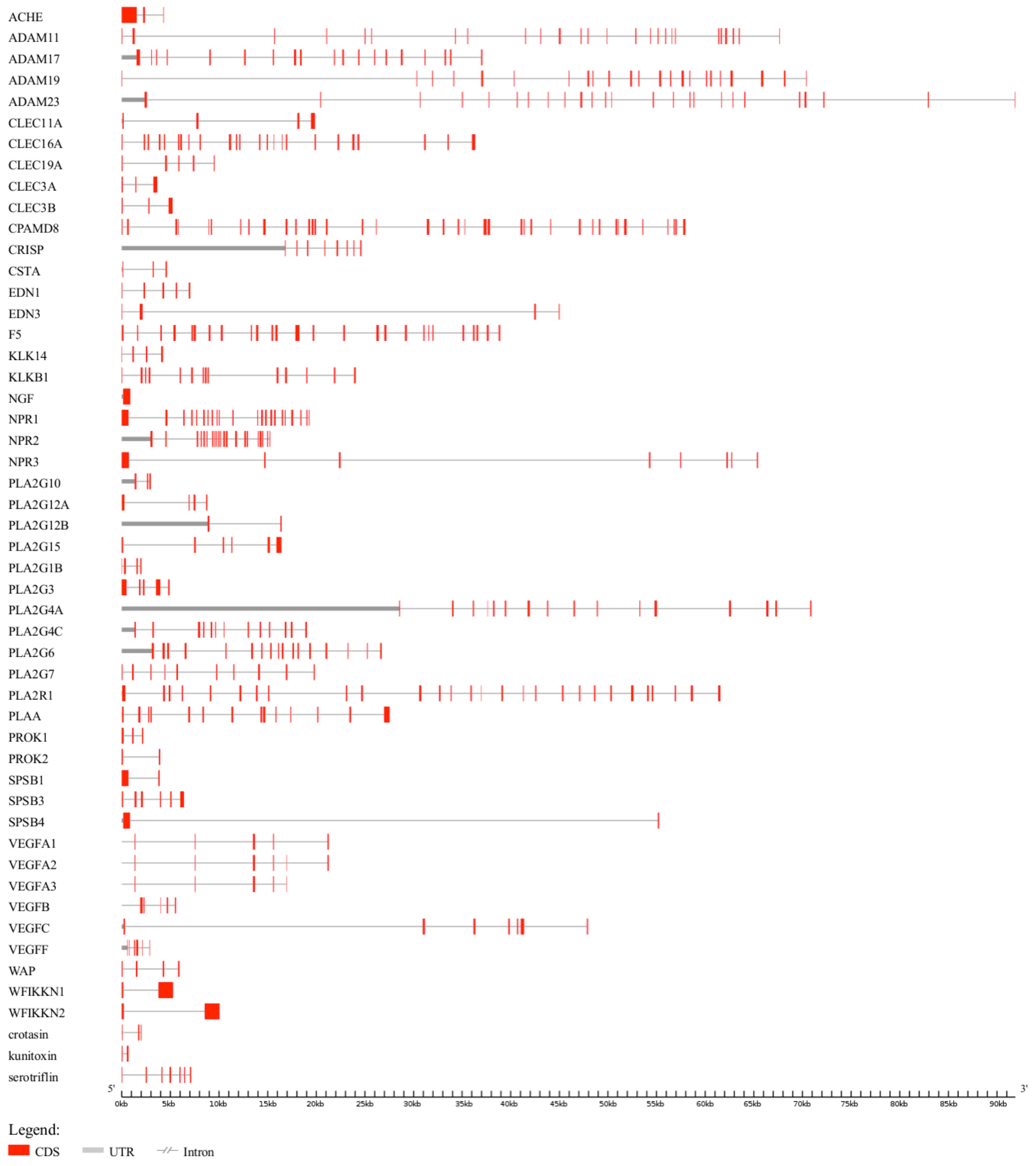

Fig. S3: Comparison of 10 toxin family protein sequences from venom-associated proteins from venom/venom gland of Russell's viper, *Daboia russelii* (Sharma *et al.,* 2016) with the putative venom homologs from genome sequencing, assembly and annotation of *Daboia russelii's* (this manuscript). Comparative analyses were performed using blastp, with the venom/venom gland associated protein sequence as the query (accession IDs from Supplementary Figure S1 of Sharma et al. 2016), against the putative venom homologs from skin. In the multiple sequence alignments, red indicates an identical match in comparison in at least one of the compared venom protein sequences. Light gray indicates non-identical amino acid.**Fig. S6:** Graphical summary of comparative domain analysis across 18 completely sequenced snake species.

I. PLA2 Family

Accession	Description
lcl|Query_172485	PLA2G10
P86169.1	RecName: Full=Basic phospholipase A2 A; Short=svPLA2; AltName: Full=Crotoxin basic chain 1; AltName: Full=Phosphatidylcholine 2-acylhydrolase
AAK49822.1	phospholipase A2 [Echis coloratus]
Q2YHJ7.1	RecName: Full=Basic phospholipase A2 Tpu-G6D49; Short=svPLA2; AltName: Full=Phosphatidylcholine 2-acylhydrolase; Flags: Precursor
CAE47155.1	ammodytin I1(C) variant [Vipera berus berus]
A8E2V8.1	RecName: Full=Acidic phospholipase A2 Tgc-E6; Short=svPLA2; AltName: Full=Phosphatidylcholine 2-acylhydrolase; Flags: Precursor
P21789.1	RecName: Full=Acidic phospholipase A2; Short=svPLA2; AltName: Full=Phosphatidylcholine 2-acylhydrolase
AAB36471.1	phospholipase A2 acidic subunit, PLA2-I subunit A [Vipera aspis, zinnikeri, venom, Peptide, 122 aa]
1OQS_G	Chain G, Crystal Structure Of Rv4RV7 COMPLEX
P31100.1	RecName: Full=Acidic phospholipase A2 RV-7; Short=svPLA2; AltName: Full=F7; AltName: Full=Phosphatidylcholine 2-acylhydrolase; AltName: Full=Phospholipase A2 inhibitor; AltName: Full=S4; AltName: Full=Viperotoxin F; AltName: Full=Viperotoxin non-toxic acidic component; Flags: Precursor
1OZ6_A	Chain A, X-ray Structure Of Acidic Phospholipase A2 From Indian Saw- Scaled Viper (echis Carinatus) With A Potent Platelet Aggregation Inhibitory Activity
P81458.1	RecName: Full=Basic phospholipase A2 RVV-VD; Short=svPLA2; AltName: Full=Phosphatidylcholine 2-acylhydrolase; AltName: Full=R1
P59071.1	RecName: Full=Basic phospholipase A2 VRV-PL-VIIIa; Short=svPLA2; AltName: Full=DPLA2; AltName: Full=P1; AltName: Full=Phosphatidylcholine 2-acylhydrolase; AltName: Full=Phospholipase A2 4; Short=PLA24
A8CG84.1	RecName: Full=Basic phospholipase A2 DsM-S1; Short=svPLA2; AltName: Full=Phosphatidylcholine 2-acylhydrolase; Flags: Precursor
P81478.1	RecName: Full=Acidic phospholipase A2 2; Short=svPLA2; AltName: Full=Phosphatidylcholine 2-acylhydrolase; AltName: Full=Phospholipase A2 isozyme II; Short=PLA2-II
P86368.1	RecName: Full=Basic phospholipase A2 3; Short=PLA23; Short=svPLA2; AltName: Full=Phosphatidylcholine 2-acylhydrolase

```
Query_172485  1  [36]GILQLAGMIQCTTGRTPL-AYIRYGCYCGWGGRGWPKDQVXXCCFKHDCCYGRAeeHSCAPKTWWYPWECQDGKAKCDDiEDKCQKMACECDRSAAKCLAKA--PYNMT-YLFWPDTQCGEKGPTC[4]  162 61%
P86169        1      HLLQFNKMIKFETRKNAIPFYAFYGCYCGWGGRGRPKDATDRCCFVHDCCYGKL--AKCNTKWDIYPYSLKSGYITCGK-GTWCEEQICECDRVAAECLRRSLSTYKYG-YMFYPDSRCRGPSETC     122
AAK49822      1  [16]HLYQFENMIYQKTGKFAIIAYSNYGCYCGWGGKGKPQDATDRCCFVHDCCYGRV--NGCDPKMGTYSYSFQNGDIVCGG-DDPCLRAVCECDRVAANCFAENLKTYNKK-YWLSSIIDCKEESEKC     138
Q2YHJ7        1  [16]SLLEFGRMIKEETGKNPLFSYISYGCYCGWGGQGQPKDATDRCCFVHDCCYGKL--WSCSPKTDIYFYYRKNGAIVCAR-GTWCEKQICECDKAAAICFRENLGTYKAE-YESYGKSRCTEKSLKC     138
CAE47155      1  [16]HLSQFGDMINKKTGIFGIMSYIYYGCYCGWGGKGKPLDATDRCCFVHDCCYGRV--NGCDPKLSTYSYSFENGDIVCGG-DDPCLRAVCECDRVAAICFGENMNTYDKK-YMLYSLLDCGEESEQC     138
A8E2V8        1  [16]SLMQFEMLIMKLAKSSGMFWYSAYGCYCGWGGQGRPQDATDRCCFVHDCCYGKA--TGCDPKKDVYTYSEENGDIVCGG-DDPCRKEVCECDKAAAICFRDNMDTYNSKtYWMFPAKNCQEESEPC     139
P21789        1      NLYQFGKMIFKMTGKSPIFSYGDYGCYCGWGGKGTPVDATDRCCFVHDCCYGRV--NSCNPKRSTYSYSFQNGGIVCDD-QNLCKRAVCECDRVAAICFGENVNTYDKK-YKDYPTSQC-TETEQ-     120
AAB36471      1      NLFQFGDMILQKTGKEAVHSYAIYGCYCGWGGQGRAQDATDRCCFAQDCCYGRV--NDCNPKMATYTYSFENGDIVCGD-NDLCLRAVCECDRAAAICLGENVNTYDKN-YEYYSISHCTEESEQC     122
1OQS_G        1      NLFQFGEMILQKTGKEVVHSYAIYGCYCGWGGQGRAQDATDRCCFVHDCCYGTV--NDCNPKTATYSYSFENGDIVCGD-NDLCLRTVCECDRAAAICLGQNVNTYDKN-YEYYSISHCTEESEQC     122
P31100        1  [16]NLFQFGEMILEKTGKEVVHSYAIYGCYCGWGGQGRAQDATDRCCFVHDCCYGTV--NDCNPKTATYSYSFENGDIVCGD-NDLCLRTVCECDRAAAICLGQNVNTYDKN-YEYYSISHCTEESEQC     138
1OZ6_A        1      NLYQFGRMIWNRTGKLPILSYGSYGCYCGWGGQGPPKDATDRCCLVHDCCYTRV--GDCSPKMTLYSYRFENGDIICDN-KDPCKRAVCECDREAAICLGENVNTYDKK-YKSYE--DCTEEVQEC     120
P81458        1      NLFQFAEMIVKMTGKNPLSSYSDYGCYCGWGGKGKPQDATDRCCFVHDCCYEKV--KSCKPKLSLYSYSFQNGGIVCGD-NHSCKRAVCECDRVAATCFRDNLNTYDKK-YHNYPPSQC-TGTEQC     121
P59071        1      SLLEFGKMILEETGKLAIPSYSSYGCYCGWGGKGTPKDATDRCCFVHDCCYGNL--PDCNPKSDRYKYKRVNGAIVCEK-GTSCENRICECDKAAAICFRQNLNTYSKK-YMLYPDFLC-KGELKC     121
A8CG84        1  [16]SLLEFGKMILEETGKLAIPSYSSYGCYCGWGGKGTPKDATDRCCFVHDCCYGNL--PDCNPKSDRYKYKRVNGAIVCEK-GTSCENRICECDKAAAICFRQNLNTYSKK-YMLYPDFLC-KGELRC     137
P81478        1      NLLQFENMIRNVAGRSGIWWYSDYGCYCGKGGHGRPQDASDRCCFVHDCCYGKV--NGCNPKKAVYIYSLENGDIVCGG-DDPCRKEVCECDKAAAICFRDNKDTYDNK-YWNIPSENCQEESEPC     122
P86368        1      SLLEFGMMILEETGKLAVPFYSSYGCYCGWGGKATPKDATDRCCFVHDCCYGNL--PDCNPKSDRYKYKRVNGAIVCEQ-GTSCENRICECDKAAAICFRRNLNTYSKI-YMLYPDFLC-KGELKC     121
```

II. SVSPs Family

Accession	Description	Links
Q9PT51.1	RecName: Full=Beta-fibrinogenase brevinase; AltName: Full=Snake venom serine protease; Short=SVSP; Contains: RecName: Full=Beta-fibrinogenase brevinase chain A; Contains: RecName: Full=Beta-fibrinogenase brevinase chain B	Related Information
BAM44992.1	hypothetical protein [Gloydius blomhoffii]	
E0Y418.1	RecName: Full=Serine protease VLSP-1; AltName: Full=Snake venom serine protease; Short=SVSP; Flags: Precursor	Related Information
E0Y420.1	RecName: Full=Serine protease VLSP-3; AltName: Full=Snake venom serine protease; Short=SVSP; Flags: Precursor	Related Information
Q9PT40.1	RecName: Full=Venom serine proteinase-like protein 2; Short=VLP2; Flags: Precursor	Related Information
E0Y419.1	RecName: Full=Beta-fibrinogenase; Short=VLBF; AltName: Full=Snake venom serine protease; Short=SVSP; Flags: Precursor	Related Information
P84788.1	RecName: Full=Thrombin-like enzyme elegaxobin-1; Short=SVTLE; AltName: Full=Elegaxobin I; AltName: Full=Fibrinogen-clotting enzyme; AltName: Full=Snake venom serine protease; Short=SVSP	
ADP88560.1	serine beta-fibrinogenase-like protein precursor [Daboia siamensis]	Related Information
O13059.1	RecName: Full=Snake venom serine protease 1; Short=SVSP 1; Flags: Precursor	Related Information
P18964.1	RecName: Full=Factor V activator RVV-V alpha; AltName: Full=Russel's viper venom FV activator alpha; Short=RVV-V alpha; AltName: Full=Snake venom serine protease; Short=SVSP	
ADI47565.1	serine protease [Echis carinatus sochureki]	
lcl|Query_10001	lcl|Query_227806 KLK14	
lcl|Query_10002	KLKB1	

```
Query_10001  1   ------------------------------------------------------------------XXXXXXX[5]XXXXKVCLGKHNLKKVEPTEQCVDVA-QAIPHPEYNKKKMNKDYMLIRL----------KPYKLPAKMQ------------------------------CANVSIVPSKQCNKAYSG-KITSHMLCAGVPQXXXXXXXGDSGGPLVCN   GLVEGVVSWGTFVCAQRGNPGVYXXXXXXXXXWIRNTIKNWIRSLYDGfex  167 74%
Query_10002  1   [369]LRLCQTSKSPVCVQKPEKQSR---VVGGSNSSAGEWPWQVSLHTvLPvQSHQCGGSIISDQWILTAAHCFK[5]EHWHIYSSILKQSEMTNETAFFKVQ-KLIIHPRYEFTETGYDIALLKLDRPMNFSALQQPICLPSEEEinMAYTECWVTGWGY-TEERGSITDMLQKVKIPLIPNAECQFQYPEHTITDKMLCAGYSXXXXXXXQGDSGGPLSCK[4]WYVVGITSWGEG-CARPGQPGVYTNVAKFVDWILEQTLEDX---------  629
46%
Q9PT51       1   ------------------------VIGGDECNINEHRFLALLY---S-ERFQCGGTLINEEWVLTAAHCDM   GNMYIYLGVHNVSVQYDDEQRRYPKKKYFCLSSRNYNQWDNDIMLIRLNRPVRNSAHIAPLSLPSGPP--SVGSVCRVMGWGTITSPNETYPDVPHCANINILDYEVCRAAYAGLPATSRTLCAGILEGGKDSCRGDSGGPLICN   GEIQGIVSWGGNICAQPREPGLYTKVFDYIDWIQSIIAGNTTVNCPP---  233
BAM44992     1   MVLIRVLANLLILQLSYAQKSSELVIGGDECNINEHRSLVVLFN--S-SSFLCGGTLINQEWVLTAAHCDS   KNFQMLFGVHSKKILNEDEQTRDPKEKFICPNKKKDDENDKDIMLIRLDSPVSNSEHIAPLSLPSSSP--TVDSVCRIMGWGTIKPTEETYPDVPHCANINILDHTVCRAAYPVLLAESSTLCAGILEGGKDTCVGDSGGPLICN   GQIQGIVSWGGHPCGQGSKPGVYTKVFDHLDWIKSIIAGNTAVTCPP---  258
E0Y418       1   MVLIRVLANLLVLHLSYAQKSSELVIGGDECNINEHPFLALMYNsTS-MKFHCSGTLLNEEWVLTAAHCDM   ENMQIYLGVHDKKNPNKDQQTRVPKEMFFCLSNKSYTPWDKDIMLIRLNSPVTYSTHIAPFSLPSSPP--TVGSVCRIMGWGAITSPNETYPDVPHCANIEIYDYSVCRKAYGGLPEKSRTLCAGVLQGGIDTCLADSGGPLICN   GQFQGIVAWGRHPCAQPQLPAFYTKVFDYSDWIQSIIAGNTAATCPS---  260
E0Y420       1   MVLIRVLANLLVLQLSYAQKSSELVIGGDECNINEHRSLVYLYNdSN---FQCGGTLINQEWVLSAAHCDM   ENMEIYLGVHNLSLPNKDQKRRDPKEKFFCLSSKNYTKWDKDIMLIKLNRPVKTSTHIAPLSLPSSPP--SVGSVCRIMGWGTVTSPNETLLDVPHCANINILNYTVCRAASPRLPTQSRTLCAGILQGGIDACKGDSGGPLICN   GQIQGIVSWGNHPCAQPLKPGHYTHVFDYTDWIQSIIAGNTTATCPP---  258
Q9PT40       1   MVLIRVLANLLVLQLSYAQKSSELVIGGDECNINEHPFPVALHTaRS-KRFYCAGTLINQEWVLTAARCDR   KNIRIIILGVHSKNVPNEDQQIRVPKEKFFCLSSKTYTRWDKDIMLIRLKKPVNDSTHIVPLSLPSSPP--SVGSVCRIMGWGTITTTKVTYPDVPHCANINMFDYSVCRKVYRKLPEKSRTLCAGILQGGIDSCKVDNGGPLICN   GQIQGIVSWGGHPCAQPHKPALYTNVFDYTDWIQSIIAGNITATCPP---  260
E0Y419       1   MVLIRVLANLLLLQLSHAQKSSELVVGGDECNINEHRSLVFLYN-SS---FGCGGTLINQEWVLSAAHCDM   ENMRIYLGWHNFSLPNMNQKRRVAKEKFFCLSSKNYTEWDKDIMLIKMNRPVTYSTHVAPLSLPSSPP--SVGSVCRIMGWGAITSPNETYPDVPHCANINILNYTVCRAAHPWLPAQSRTLCAGILQGGIDTCKGDSGGPLICN   GQIQGIVSWGDNPCAQPLKPGHYTNVFDYTDWIQSIIAGNTTATCPP---  257
P84788       1   ------------------------VIGGDECNINEHPFLVLVY---Y-DDYQCGGTLINEEWVLTAAHCNG   KNMEIYLGVHSKKVPNKDVQRRVPKEKFFCDSSKTYTKWNKDIMLIRLDRPVRKSAHIAPLSLPSSPP--SVGSVCRVMGWGTITSPQETYPDVPHCAKINLLDYSECRAAYPGLPPKSRTLCAGVLEGGKDTCGGDSGGPLICN   GQFQGIVSWGGDPCAQPHEPGSYTNVFDHLDWIKGIIAGNTDATCPL---  233
ADP88560     1   MVLIKVLANLLVLQLSYAQKSSELVVGGDECNINEHRSLVFLYN----NSFGCSGTLINQQWVLSAVHCDM   ENVRIYLGVHNLTLRNNAE-IRLPEERFFCLSNKNYTKWDKDIMLIKLDRPVKTSTYIAPLSLPSSPP--RVGSVCRIMGWGAITSPNETFPGVTHCANINILPYSVCRAAYKGLPAQSRTLCGGILEGGIGSCMGDSGGPLICN   GEMHGIVAWGDDTCAQPHKPVHYTKVYDYTDWIQSIIAGNTAATCPP---  256
O13059       1   MVLIRVLANLLILQLSYAQKSSELVVGGDECNINEHRSLVAIFN--S-TGFFCSGTLINQEWVVTAAHCDS   NNFKMKFGAHSQKVLNEDEQIRNPKEKFICPNKKNNEVLDKDIMLIKLDSSVSNSEHIAPLSLPSSPP--SVGSVCRIMGWGSITPTKVTYPDVPYCANINLLDDAECKPGYPELLPEYRTLCAGIVQGGKDTCGGDSGGPLICN   GQFHGIVSYGAHPCGQSLKPGIYTTVFDYNDWIKSIIAGNTAATCPP---  258
P18964       1   ------------------------VVGGDECNINEHPFLVALYTsTS-STIHCGGALINREWVLTAAHCDR   RNIRIKLGMHSKNIRNEDEQIRVPRGKYFCLNTKFPNGLDKDIMLIRLRRPVTYSTHIAPVSLPSRSR--GVGSRCRIMGWGKISTTEDTYPDVPHCTNIFIVKHKWCEPLYPWVPADSRTLCAGILKGGRDTCHGDSGGPLICN   GQIQGIVAGGSEPCGQHLKPAVYTKVFDYNNWIQNIIAGNRTVTCPP---  236
ADI47565     1   MVLIRMLANLLVLQLSYAQKSSELVTGGDECNINEHPFLVALHTaRS-KRFHCTGTLINEQWVLTAARCNR   KNIRIKLGVHNKNVRNENEEMRVPAEKVFCVSSKTYTRWDKDIMLIKMKRPVNNSTHIAPLSLPSNPA--SVGSVCRIMGWGTITTTKVTYPDVPHCANIKIFDYSVCRGAYRKLPEKSRTLCAGVLEGGIDSCKADTGGPLICN   GQFQGIASWGGQPCAQPLKPALYT--------------------------  237
```

III. RVV X light chain (Alpha subunit)

Accession	Description	Links
Q696W1.1	RecName: Full=Snaclec coagulation factor X-activating enzyme light chain 2; AltName: Full=VL factor X activator light chain 2; Short=VLFXA light chain 2; Flags: Precursor	Related Information
ADK22819.1	factor X activator light chain 2 [Daboia siamensis]	
ADJ67473.1	factor X activator light chain 2 [Daboia russellii russellii]	
lcl|Query_10001	lcl|Query_119202 CLEC19A	
lcl|Query_10002	PLA2R1	

```
Query_10001  1     [ 14]XXXXXXXXXXXXXXXQALPESILSYSCPLFWTEYEGYCYRYFPIN-KTWAEADLYCAEFSiglKSAKLXXXXSWEENVFVYDLVNSRVPGIPTDIWMGL--NDLRQESNFEWTDGSPYDYNYWDGN[10]EEEDCVQIWYrySSXXXXXXXXXCSREFPFVCKIPSLA[   3]  188  44%
Query_10002  1     [145]PWCATTSHYEQDEKWGLCPNSGSGCDIFWKINTDTQICYQINLLSvLSWNEARVACQGQG-----AELLSITDMREQKYIGGLIXXXNTK-EAMIWTGL--NQLDETAGWQWSDGAPLALVNWLMD[ 8]--AHCKAFNLk---IQNDWPSYSCESGLPYVCKKYLNA[1107]  1411 29%
Q696W1       1          MGRSISVSFGLLAVFLSLSGTGAGLDCPPDSSPYRYFCYRVFKEQ-KNWADAERFCAERP---NNGHLVSIESMEEAEFVAQLLSKITGKFITHFWIGLRIEDKKQQCRSEWSDGSSVSYDNLLKR    EFRKCFGLEK--GTGYRSWFNLNCEEPYPFVCKVPPNC        158
ADK22819     1          MGRFISVSFGLLVVFLSLSGTGAGLDCPPDSSLYRYFCYRVFKEH-KTWEAAERFCMEHP---NNGHLVSVESMEEAEFVAKLLSNITEKFITHFWIGLMIKDKEQECSSEWSDGSSVSYDNLDKR    EFRKCFVLEK--ESGYRMWFNRNCEERYLFVCKVPPEC        158
ADJ67473     1          MGRFIFVSFGLLAVFLSLSGTGAGLDCPPDSSPYRYFCYRVFKLR-KSWEAAERFCMEHP---NNGHLVSIESMEEAEFVAKLLSNTTGKFITHFWIGLRIKDKEQECSSEWSDGSSVSYDNLGKE    EFRKCFVLQK--ESGYRMWFNHKCEEPYPFVCKVPPEC        158
```

IV. RVV X light chain (Beta subunit)

Accession	Description	Links
Q4PRC8.1	RecName: Full=Snaclec 5; AltName: Full=C-type lectin-like 5; Flags: Precursor	Related Information
Q4PRC9.1	RecName: Full=Snaclec 4; AltName: Full=C-type lectin-like 4; Flags: Precursor	Related Information
AAB22478.1	coagulation factor X activating enzyme light chain, RVV-X-light chain, LC1=metalloproteinase with disintegrin (platelet aggregation inhibitor)-like and C-type lectin-like domains [Vipera russelli=Russell's viper, Siamensis, venom, Peptide, 123 aa]	
lcl|Query_10001	lcl|Query_125072 CLEC19A	
lcl|Query_10002	PLA2R1	

```
Query_10001  1     [ 14]XXXXXXXXXXXXXXXQALPESILSYSCPLFWTE--YEGYCYRYFPI   NKTWAEADLYCAEFSiglKSAKLXXXXSWEENVFVYDLVNSRV-PGipTDIWMGLN--DLRQESNFEWTDGSPYDYNYWD[12]EEEDCVQIWYRYS[4]XXXXXXCSREFPFVCKIPS[  5]  188 48%
Query_10002  1     [606]--------------------DESFNSCMHGWESdhYLLNCYKVFHS[5]KRTWQEAETLCQDFG-----AHLASFKQVLEEDFLNKLLNTMFhXDdeRQFWIGFNkrNPLSGGSWEWSDGTPVSSAFLE[ 8]--KNCAAYKTNGT   -VFPLHCNSEHEWICKIPK[662]  1411 47%
Q4PRC8       1          MGRFISISFGLLVMFLSLSGTGAKQDCLSDWSF--YEGYCYKVFNE   KKTWEDAEKFCTEQH---KGSHLLSLHNIAEADFVLKKTLAML-KD--GVIWMGLN--DVWNECNWGWTDGAKLDYKAWN    EGTNCFVFKIAKN   HWSHMDCSSTHNFVCKFRV        148
Q4PRC9       1          MGRFISISFGLLVVFLSLSGTEAAFCCPSGWSA--YDQNCYKVFTE   EMNWADAEKFCTEQK---KGSHLVSLHSREEEKFVVNLISENL-EY--PATWIGLG--NMWKDCRMEWSDRGNVKYKALA    EESYCLIMITHEK   VWKSMTCNFIAPVVCKF--        146
AAB22478     1          --------------VLD---------CPSGWLS--YEQHCYKGFND   LKNWTDAEKFCTEQK---KGSHLVSLHSREEEEFVVNLISENL-EY--PATWIGLG--NMWKDCRMEWSDRGNVKYKALA    EESYCLIMITHEK   EWKSMTCNFIAPVVCKF--        123
```

V. RVV X heavy chain

Accession	Description	Links
CAJ01689.1	Group III snake venom metalloproteinase [Echis ocellatus]	
ADI47578.1	metalloproteinase [Echis carinatus sochureki]	
2E3X_A	Chain A, Crystal Structure Of Russell's Viper Venom Metalloproteinase	Related Information
Q7T046.1	RecName: Full=Coagulation factor X-activating enzyme heavy chain; AltName: Full=Coagulation factor X-activating enzyme chain alpha; AltName: Full=Snake venom metalloproteinase; Short=SVMP; AltName: Full=VL factor X activator; AltName: Full=VLFXA heavy chain; Contains: RecName: Full=Coagulation factor X-activating enzyme heavy chain alternate form; Flags: Precursor	Related Information
Q2UXR0.1	RecName: Full=Zinc metalloproteinase-disintegrin-like Eoc1; AltName: Full=Snake venom metalloproteinase; Short=SVMP; Flags: Precursor	Related Information
ADJ67475.1	factor X activator heavy chain [Daboia russellii russellii]	
lcl|Query_10001	ADAM11	
lcl|Query_10002	ADAM17	
lcl|Query_10003	ADAM19	
lcl|Query_10004	ADAM23	

```
Query_10001  107  [13]CCLLLCHSGXXXADPPPGDSRrwV[23]NSGGEIQSYQLdTTVRKETGGSRGDDRKGGSPVI[ 2]AQVSFLIPAFGLTFTXXXXXXXHLLSLNYVERHFGXXXXXXXXX-GTSEHCYYQGNLRGEANSFVALSTCHGLXGVFSDGKD-VYLLKPH  184
Query_10002  85   [10]--RLLPALFLLLLLXXXGVSA--L[23]LSVSNIQQHSV-----------RRRDLQPESHIE[ 2]----ISFSALHRYFQLYLRSTADHFSKNFQALILDGQGNEREYPVQWXXXXXXXXXGQSNSKVIAHIGESDFTVRINTDAEE--YNVEPL  162
Query_10003  24       ------------------------    ---------------------XXXXXXXXXXXXX    XXXVHGISDRDEILIKLNLYRWYLFAPDYTETHYTKTGTAQTISLNKTDNCFYHGRVRAIRESXXXXXXXXXXXXGLILISNNiSYVLEPL  103
Query_10004  95   [13]KTETILANEDTAFQQTNNSSS--K[10]---------EI-TLPSRLIYYLNKDSESPYHILD[15]AQASFQIEAFGSTFILDLSLNNDLLSADYVEIHYEDDKPQYS-K--GGEHCYYHGSIRGIPKSKAAISTCNGLHGMFEDNTY-VYLIEPL  170
CAJ01689     66       MIQVLLVIICLAVFPYQGCSI--I    LESGNVNDYEV-VYPQKVAALPKGAIQQPEQKYE    DAIQYEFEVKGEPVVLHLEKNKGLFSEDYSETHYSPDDREITTKPSVEDHCYYHGRVQNDAESTASISACNGLKGHFTLRGE-TYFIEPL  144
ADI47578     19       ------------------------    -------------------------KQPEQKYE    DTMQYEFKVKGEAVVLHLEKNKGLFSEDYTETHYAPDGREITTKPAVEDHCYYHGRIQNDADSSASISACNGLKGHFKLRGE-MYFIEPL  97
2E3X_A                ------------------------    ----------------------------------    -------------------------------------------------------------------------------------------
Q7T046       66       MMQVLLVTISLAVFPYQGSSI--I    LESGNVNDYEV-VYPQKITALPEEAVQQPEQKYE    DTMQYEFEVNGEPVVLHLEKNKDLFSEDYSETRYSPDGRETTTKPPVQDHCYYHGRIQNDAYSSASISACNGLKGHFKLQGE-TYLIEPL  144
Q2UXR0       65       -MQVLLITISLAVLPYLGSSI--I    LESGIVNDYEV-VNPQKVTAMLKGAVKQPEQKYE    DTMQYEFKVKGEPVVLHLEKNKGLFSEDYSETHYSPDGREITTNPPVEDHCYYHGRIQNDADSSASISACNGLKGHFKLRGE-MYFIEPL  143
ADJ67475     66       MMQVLLVTISLAVFPYQGSSI--I    LESGNVNDYEV-VYPQKVTAMPKGAVKQPEQKYE    DTMQYEFEVNGEPVVLHLEKNKILFSEDYSETHYYPDGREITTNPPVEDHCYYHGHIQNDGHSSASISACNGLKGHFKLRGE-MYFIEPL  144

Query_10001  185  DSLEHQ[5]PHQIYQTPVPRMSR--GCTEPgCLFSALN   YHIPPGLPKLRrRRQXXXXXXXVHSETKFIELIIVNDHQLXXQMRRSVSL---TSNFAKSVVNLADVXFKEqLNTRIVLVAMETWASGDKIQTDEDSLETL    REFMKYRQESL--P  261
Query_10002  163  WRFLNN[4]RLLVYRSEDIKDISrlESPKV-CGYVKPE[4]WGXXXXXXXXxENIPRRRRATPDPLKNTCKMLVVADHRFFKNMGRGEESt--TINYLIELIDRVDDIYRNtSWDNAHFNGYGVQIEQIIVHSEPENLPPK[12]EEKDAWEVKQLLEQ
Query_10003  104  P-DSQE   QHLIYRSEHLKVPH--GS----CGNEHLE   SKVTDWLAELT-GRTETRHHRXXXXXXXXXXXXXXXXXXXXXXXXXXXXxxxTKKKLVEAANYVDKFYRS-LNIRIALVGLEVWNDYNKCDISENLHSTTL    WSFLAWRRRLLIHK
Query_10004  171  ELTHSX[5]LHVIRRTFGSNAFS--QLEDLgT-DSTSD   WSFLSELQWLRrRRRAVSAVRGIFEEMKYLELMIVNDHKMFRKHRSSHSN---TNNFAKSVVNLVDAIYKEqLNTRVVLVAVETWSDRDRIRVHSDPRQML    HDFSRYRQNFI--K
CAJ01689     145  KIPDSE   AHAVYKYENIEEED--EAPKM-CGVKQSN   RESDEPIKKAS-GLIVPSQKRRL-D-QKFIELVMVVDHSMVTKYNNDSTA---VRTWIYEMVNTVNEIYLP-LNIRVPLVGIVFWSNRDLINVTFTADDTM    DSFGEWRASYLLNR  212
ADI47578     98   KIPDSE   AHAVYKYENIEKED--EAPKI-CGVKKTN   WESDKSIQEAS-QLNLTPEQQRYLNSEKHIKVAIIADYLIYRKYGRNLFT---IRTRIYEIINILNAIYRA-FHMHVALVFLEIWSNGDKINVLPAANVTL    DLFGKWRLSDLLNR  167
2E3X_A       1    ------   ---------------------------   ------------LVSTSAQ--F--NKIFIELVIIVDHSMAKKC--NSTA---TNTKIYEIVNSANEIFNP-LNIHVTLIGVEFWCDRDLINVTSSADETL    NSFGEWRASDLMTR  22
Q7T046       145  KIPDSE   AHAVYKYENIEKED--EAPKM-CGVTQTN   WESDEPIKKAS-QLVATSAKRKF--HKTFIELVIVVDHRVVKKY--DSAA---TNTKIYEIVNTVNEIFIP-LNIRLTLIGVEFWCNRDLINVTSSADDTL    DSFGEWRGSDLLNR  212
Q2UXR0       144  KIPDSE   AHAVYKYENIEEED--EAPKM-CGVKHTN   RESDKSIKKAS-QLNLTPEQQRYLNTPKHIKVAIVADYLIFRKYGRNLFT---IRAKIYEILNILNEIYKA-FNIHVALVFLEIWSNGDKINLFPAANVTL    DLFGKWRERDLMNR  213
ADJ67475     145  KLSNNE   AHAVYKYENIEKED--ETPKM-CGVTQTN   WESDKPIKKAS-QLVSTSAQ--F--NKAFIELIIIVDHSMAKKC--NSTA---TNTKIYEIVNSANEIFNP-LNIHVTLIGVEFWCDRDLINVTSSADETL    DSFGEWRASDLMTR  210

Query_10001  333  EHSD   AAHLFSGHTFRSSRSGTAYV   GGICS   VARGGXXXXYGTV   --GAMAVTLAQTLGQNIGMMWNKHR[5]CRCPd-lWLGCIMEDTGYYl-----PRKFSRCSVDEFNQFLAEGGGSCLFNKPLKX--XXXXXCGNGFVEAGEECDCG  472
Query_10002  330  FSYD[9]LAHLFTYQDFDMGTLGLAYV[8]GGICP[11]VYLNSGLTSTKNY[4]LTKEADLVTTHELGHNFGAEHDPDS[3]CAPNedqGGKYVMYPIAVSgdhXXXXXXXXCSKASILQTIQAKAPECFKERSNK-------VCGNSRVDEGEECDPG  501
Query_10003  245  K-HD   NAQLITGMSY-GTTIGLAPL   MAMCS   AYQSGXXXXDHSE   NAIGVAATMAHEMGHNFGMNHDSDG   CCFAsprDGGCIMAAATGA---PFPRVFNECNRKELERYLQSGGGMCLYNMPDTRTLYGGQRCGNGYLEEGEECDCG  383
Query_10004  318  QHAD   AVHLLSYXXFHYKRSSLSYF   GGICS   VARGVGVNEYG--   LSWAIAQELSQSLAQNLGIQWEPAS[5]XXXXx-xXXXXXXXXX-VY---H-SRKFSKCSIAEYRDFLLRGGGFCLFNRPTK--VXXXTECGNGYVEPGEDCDCG  456
CAJ01689     285  KRHD   YAQLLTNITLDFDSLGMAFI   DGMCK   SDRSVGLIRDDSS   TTFRTAVIMAHEMGHSLGMEHDSRS   CKCA---ASPCIMSKALGK---QPTKVFSSCSYDDYRMYLAKYKPKCILDPPLRKDIASPAVCGNKIWEEGEECDCG  422
ADI47578     240  REHD   NAQLLTGINFDGPTAGLGYV   GSMCE   PQYSAAIVQDHNK   INILVAMAMAHELGHNLGMNHDEKF   CTCG---AKSCIMSGTLSC---EGSFRFSNCSQEENRKYLIRKMPQCILKKPLKTDIVSPPVCGNYLVELGEDCDCG  377
2E3X_A       93   KSHD   NALLFTDMRFDLNTLGITFL   AGMCQ   AYRSVGIVQEQGN   RNFKTAVIMAHELSHNLGMYHDGKN   CICN---DSSCVMSPVLSD---QPSKLFSNCSIHDYQRYLTRYKPKCIFNPPLRKDIVSPPVCGNEIWEEGEECDCG  230
Q7T046       283  KRHD   NAQLFTDMKFDLSTLGITFL   DGMCQ   AYRSVGIVQEHGN   KNFKTAVIMAHELGHNLGMYHDRKN   CICN---DSSCIMSAVLSS---QPSKLFSNCSNHDYRRYLTTYKPKCILNPPLRKDIASPPICGNEIWEEGEECDCG  420
Q2UXR0       286  KNHD   NTQLLTGMNFDGPTAGLGYV   GTMCH   PQFSAAVVQDHNK   INFLVALAMAHELGHNLGMTHDEQF   CTCG---AKSCIMSATLSC---EGSYRFSNCSREENRRYLINKMPQCILIKPSRTDIVSPPVCGNSLVEVGEDCDCG  423
ADJ67475     281  KSHD   NALLFTDMRFDLNTLGITFL   AGMCQ   AYRSVGIVQVQGN   RNFKTAVIMAHELSHNLGMYHDGKN   CICN---DSSCVMSPVLSD---QPSKLFSNCSIHDYQRYLTRYKPKCILYPPLRKDIVSPPVCGNEIWEEGEECDCG  418

Query_10001  473  SfaxXXXXXXXCC--KKCTLTHDAMCS--DXXXXXXXXXXXXXVTCREAV-NECDIPETCTGDSSQCPPNVHKQDGYFCDNEQGRCYGGRCKTRDRQCNALWGRA--SAERFCYEKlNVEGTERGNCGHE-GSRWMPCVKQDVLCGYLLCANA[17]XXXXXXXXXxxgGHVQLVD[1]TDLSYVEDGTPCGPNMLCLDHK-CLPVSAFXXXXXXXxxxXXX[ 89]  785 43%
Query_10002  502  L---LNLQNDLCC-TSDCKLKPNAKCSdrNSPCCKRCQFESADKKCQEAInATCKGESYCTGNSSDCPPPGNALDDTVCVD-LGKCKDGECVPFCEWEKSLRSCAXXETDNSCKIC---CRDEFGRCSPY----------RDINQNFLYLRKG    -----KPCT----------   --VGFCDMNGKCDKRVQDVIERlWVFIDQLSINTFGKflaDNI[158]  839 37%
Query_10003  384  E---VEECHNPCCNAADCTLKQGAECA--HGICCQKCKLVSPGTLCREQA-RTCDLPEFCSGKSPFCPANFYQLDGTPCENGRAYCYNGMCLTYEQQCLQLWGYGARPAPDLCFEKvNAAGDSYGNCGKDiTGNYRKCDTRNAKCGKIQCQSS[17]LNGREIHCRgthVYLNEED[4]LDPGLVMTGTKCGDSHVCFEGH-CQ--NTSFFESGDC---GKK[258]  865 53%
Query_10004  457  -----IQTXXXXXXXKKCSLSNGAHCS--DGPCCNETCLXXXXXXXXXXXxNECDITEICTGDSGQCPPNLHKQDGFACDSNQGRCYNGECKTRDNQCKFIWGNKASGSHKHCYEKlNTEGTQKGNCGKD-GDKWIPCHKHDVFCGLLLCANL[17]FFHQXXXXXcscAHVLLDD[1]TDLGYVEDGTPCGPNMMCMDRR-CLSIQSLNVSSCPMdanGKV[ 88]  768 41%
CAJ01689     423  S---PEDCRNPCCDAETCELFPAAECA--DGPCCHKCKIRTAGTICRPAR-DECDVTEHCTGQSAECPRNELQRNGEPCLDKLGYCYNGDCPIMRNQCISLFGSRATVAEDSCFQQ-NLNGSEHGYCAKE-NGRKIPCAPQDVKCGRLYCLDN    SSRKKNPCK---MHYLNAD   QHKGMVEPGTKCEDGKVCINRK-CVDVKTAYYSTTGF---SQI  622
ADI47578     378  T---PTFCQNPCCNAATCKLTPGSQCA--DGECCDQCRFRRAGTECRPAK-DECDMADLCNGQSDECPKDQFQRNGHPCQNNNGYCYNGKCPVMGNQCISLFGSRATVAEDACFQF-NRLGSDYGYCRKE-NGIKIPCAPEDVKCGRLYCFDN    LPEHKNPCQ---IYYTLRD   ENKGMVEPGTKCENGKVCINGK-CVDVNTAY------------  568
2E3X_A       231  S---PANCQNPCCDAATCKLKPGAECG--NGLCCYQCKIKTAGTVCRRAR-DECDVPEHCTGQSAECPRDQLQQNGKPCQNNRGYCYNGDCPIMRNQCISLFGSRANVAKDSCFQE-NLKGSYYGYCRKE-NGRKIPCAPQDVKCGRLFCLNN    SPRNKNPCN---MHYSCMD   QHKGMVDPGTKCEDGKVCNNKRqCVDVNTAYQSTTG-------  427
Q7T046       421  S---PKDCQNPCCDAATCKLTPGAECG--NGLCCEKCKIKTAGTVCRRAR-DECDVPEHCTGQSAECPADGFHANGQPCQNNNGYCYNGDCPIMTKQCISLFGSRATVAEDSCFQE-NQKGSYYGYCRKE-NGRKIPCAPQDIKCGRLYCLDN    SPGNKNPCK---MHYRCRD   QHKGMVEPGTKCEDGKVCNNKRqCVDVNTAY------------  612
Q2UXR0       424  S---PGYCRNPCCNAATCKLTPGSQCA--DGECCDQCRFTRAGTECRPAR-DECDKADLCTGQSAECPADQFQRNGQPCQNNSGYCYNGICPVMRNQCISLFGSRAIVAEDACFQF-NSLGIDYGYCRKE-NGRKIPCAPEDVKCGRLYCFDN    LPEHKNPCQ---IFYTPRD   EDKGMVDPGTKCENGKVCINGK-CVDVNTAY------------  614
ADJ67475     419  S---PADCQNPCCDAATCKLKPGAECG--NGLCCYQCKIKTAGTVCRRAR-NECDVPEHCTGQSAECPRDQLQQNGQPCQNNRGYCYNGDCPIMRNQCISLFGSRATVAKDSCFQE-NLKGSYYGYCRKE-NGRKIPCAPQDVKCGRLFCLNN    SPRNKNPCN---MHYSCMD   QHKGMVDPGTKCEDGKVCNNKRqCVDVNTAYQSTTGF---SQI  619
```

VI. CRISPs (Helveprins) Family

Accession	Description	Links
ACE73567.1	cysteine-rich seceretory protein Dr-CRPK [Daboia russelii]	
ACE73571.1	cysteine-rich seceretory protein Pg-CRP [Cerrophidion godmani]	
ACE73568.1	cysteine-rich seceretory protein Dr-CRPB [Daboia russelii]	
ACE73566.1	cysteine-rich seceretory protein Cv-CRP [Crotalus viridis]	
ACE73560.1	cysteine-rich seceretory protein Ch-CRPKa [Crotalus horridus]	
ACE73575.1	cysteine-rich seceretory protein Da-CRPa [Deinagkistrodon acutus]	
AAB48565.1	prepro-cysteine-rich venom protein [Protobothrops mucrosquamatus]	
lcl|Query_10001	lcl|Query_208126 CRISP	
lcl|Query_10002	serotriflin	

```
Query_10001  1  [40]SVDFDSESPRKPEIQNEIVDLHNSLRRSVTPTASNMLKMEWYPEAAANAERWAFRCILNHSPYNSRVIXXXKCGENLFMSSHPSPWTRVIQLWYDEYKNFKYGVGANPPNAVIGHYTQVVWYKSYLVGCAAARCPSSSYNYFYVCHXXXXXXXXXXXXXXXXXXXXXXXXXXXXXXXXXXNPCRHENVFSNCNDLVKHQGCQNNNMKSNCPGSCFCRNEIXx  262 92%
Query_10002  1  [19]IVDFDSESPRKPEIQNEIVDLHNSLRRSVTPTASNMLKMEWSSNAAQNAKRWADRCSFAHSPQHLRTMGKLKCGENLFMSSHPSPWTRVIQLWYDEYKNFKYGVGANPPNAVIGHYTQVVWYKSYLVGCAAARCPSSSYNYFYVCHYXXXXXXXXXXXXXXXXXXXXXXXXXXXXXXXXXNPCRHENVFSNCNDLVKHQGCQNNNMKSNCPGSCFCRNEIXx  241 84%
ACE73567     1  [19]SVDFDSESPRRPEIQNEIVDLHNSLRRSVTPTASNMLKMEWYPEAAANAERWAFRCILNHSPYNSRVIGGIKCGENIYMSPYPMKWTAIIHEWHKEKKDFVYGQGASPANAVVGHYTQIVWYKSYRSGCAAAYCPSSEYNYFYVCQYCPAGNIIGKIATPYTSGPPCGDCPSACDNGLCTNPCSHHDEFTNCKDLVKQ-GCHSNYLKTKCPASCFCHNEII-  239
ACE73571     1  [19]NVDFDSESPRKPEIQNKIVDLHNSLRRSVNPTASNMLKMEWYPEAAANAERWAYRCIESHSPRNSRVLGGIKCGENIYMSSIPITWNEIIHAWHGEYKDFIFGVGANPPNAVTGHYTQIVWYKSYRIGCAAAYCPSSEYSYFYVCQYCPAGNIRGKTATPYKSGPPCGDCPSACDDGLCTNPCTREDKYTNCNSLVQKSGCQDTWMQSNCPAICFCQNKII-  240
ACE73568     1      SVDFDSESPRKPEIQNEIVEFHNSLRRSVNPTASNMLKMEWYPEAAANAERWAFRCILDHSPYNSRVIGGIKCGENIYMSSNPIKWIEIIRKWHDEKKNFIYGKGANPSNAVVGHYTQVVWYKSYRIGCAAAYCPSSAYKYFYVCQYCPAGNIVGRTATPYKSGQPCGDCPSACDNGLCTNPCRREDVFTNCIDMAKGRSCQDNYMKLNCPAACFCRNEIK-  221
ACE73566     1      SVDFDSESPRKPEIQNKIVDLHNFLRRSVNPTASNMLKMEWYPEAAANAERWAYRCIESHSPRDSRVLEGIKCGENIYMSPVPMKWTEIIHAWHGENKDFKYGIGADPPNAVIGHYTQVVWYKSYRIGCAAAYCPSSEYSYFYVCQYCPAGNIIGKTATPYKSGPPCGDCPSACDNGLCTNPCTKEDKYTNCKSLVQQAGCQNERMQSDCSAICFCQNKII-  221
ACE73560     1      SVDFDSESPRKPEIQNEIVDLHNSLRRSVNPTASNMLKMEWYPEAAANAERWAYRCIESHSPRDSRVLEGIKCGENIYMSPVPIKWTEIIHGWHGENKNFKYGIGAEPSNAVTGHFTQIVWYKSYRVGCAAAYCPSSKYSYFYVCQYCPAGNIRGKTATPYKSGPPCGDCPSACDNGLCTNPCTKEDKYTNCKSLVQQAGCEDKQIQSDCSAICFCQNKII-  221
ACE73575     1      SVDFDSESPRKPEIQNEIVDLHNSLRRSVNPTASNMLKMEWYPEAAANAERWAYRCIEEHSSRDSRVLEGIKCGENIYMSPNPMKWTEITHAWHGEYKDFKYGVGADPPNAVTGHYTQIVWYKSHHLVC-CCLCPLSKYSYFYVCQYCPAGNIIGKIATPYTSGPPCGDCPSACDNGLCTNPCTQEDKYTNCKSLLQQDSCQDAGMQSKCSASCFCQNKII-  220
AAB48565     1      --------------------------------------MEWYPEAAANAERWAYRCIESHSSRDSRVIGGIKCGENIYMSPYPAKWTDIIHAWHGEYKDFKYGVGAVPSNAATGHYTQIVWYKSYRGGCAAAYCPSSKYRYFYVCQYCPAGNMIGKTATPYTSGPPCGDCPSDCDNGLCTNPCTQENTYSNCNSLVQQSSCQDNNMKTKCPASCFCQNKII-  183
```

VII. Snaclec Family

Accession	Description	Links
Q38L02.1	RecName: Full=Snaclec dabocetin subunit alpha; AltName: Full=C-type lectin-like 8; Flags: Precursor	Related Information
ADK22822.1	dabocetin beta subunit [Daboia russellii russellii]	
ADK22832.1	P31 beta subunit [Daboia siamensis]	
Q719L9.1	RecName: Full=Snaclec crotocetin-1; Short=CRC1; Flags: Precursor	Related Information
B4XSY9.1	RecName: Full=Snaclec A14; AltName: Full=C-type lectin A14; Flags: Precursor	Related Information
P81113.1	RecName: Full=Snaclec alboaggregin-A subunit beta; AltName: Full=Alboaggregin-A subunit 3; Short=AL-A subunit 3	
lcl|Query_10001	lcl|Query_48204 PLA2R1	
lcl|Query_10002	CLEC19A	

```
Query_10001  1   [165]-----------------SGSG---CDIFWkiNTDTQICYQINLlSVLSWNEARVACQGQG----AELLSITDMREQKYIGGLIXXXNTKEAMI--WTGLN--QLDETAGWQWSDGAPLALVNWLMDP[ 7]-AHCKAFNLKi--QN-DWPSYSCESGLPYVCKKYLNA[1107]  1411 51%
Query_10002  1   [ 14]XXXXXXXXXXXXXXQALPESILSYSCPLFW--TEYEGYCYRYFP-INKTWAEADLYCAEFSiglKSAKLXXXXSWEENVFVYDLVNSRVPGIPTD-IWMGLN--DLRQESNFEWTDGSPYDYNYWDGNQ[10]EEDCVQIWYRySSXXxXXXXXXCSREFPFVCKIPSLA[   3]  188 53%
Q38L02       1        MGRFISVSFGLLVVFLSLSGTGAD--CPSEW--SSHEGHCYKVFK-LLKTWEDAEKFCTQQA---NGWHLASIESVEEANFVAQLASETLTKSKYH-AWIGLRdqSKRQQCSSHWTDGSAVSYETVTK--    YTKCFGLNKE-TKYH-EWITLPCGDKNPFICKSWVLH        154
ADK22822     1        MGRFISVGFGLLVVFLSLSGTGAKQDCLSDW--SFYEGYCYKVFN-EKKTWEDAEKFCNEQV---NGGYLVSFRSSEEMDFVIRMTFPIFRFD--F-FWIGLR--DFWRDCYWRWSDGVNLDYKAW--SR    EPNCFVSK---TTDN-QWLRWNCNDPRYFVCKSRVSC        150
ADK22832     1        MGRFIFVSFGLLVVFLSLSGTEAGFSCPNGW--SSFGQHCYKVIE-PLKNWTDAEKFCREQH---KGSHLASIHSSEEEAFVSKVASKVLKFGS---VWIGLN--DPWHNCNWEWSDNARFDYKAMTRRP    --YCTVMVLK--PDRiFWFNRGCEKFVSFVCKFLA--        150
Q719L9       1        MGRFIFVSFGLLVVFLSLSGTGADFDCPSGW--SAYDQYCYRVIK-QLKTWEDAEWFCTKQA---KGAHLVSVESAGEADFVAQLVAENIKQNKYY-VWIGLRiqNKGQQCSTKWSDGSSVNYENLLKSY    SKKCFGLKKE-TEFL-QWYNTDCEEKNLFVCKFPPQR        158
B4XSY9       1        MGRFIFVRVGLLVVFLSLSGTGADFDCPPDW--SAYDQHCYKAFD-EPKRSGDAEKFCTQQA---NGGHLVSIESVEEAEFVAQLISENIKTSADY-VWIGLWnqRKAPYCVSKWTDGSSVIYKNVIERF    IKNCFGLEKE-TNYR-TWFNLSCGDDYPFVCKSPA--        156
P81113       1        -----------------------GFDCPFGW--SSYEGYCYKVYN-KKMNWEDAESFCREQH---KRSHLVSFHSSGEVDFV---VSKTFPILRYDfVWMGLS--DIWKECTKEWSDGARLDYKAWSG--    KSYCLVSK---TTNN-EWLSMDCSRTRYPVCKFXG--        125
```

VIII. KSPI Family

Accession	Description	Links
lcl|Query_140082	kunitoxin	
Q2ES47.1	RecName: Full=Kunitz-type serine protease inhibitor 4; AltName: Full=Kunitz protease inhibitor 4; AltName: Full=Kunitz protease inhibitor IV; Flags: Precursor	Related Information
CAL69605.1	trypsin inhibitor-4 precursor [Daboia siamensis]	Related Information
CAL69613.1	trypsin inhibitor-5 precursor [Daboia siamensis]	Related Information

```
Query_140082  1    MSSGDLLLLLGFFTLWAQLIPXXXXXXXXXXXXXXXXGPCKAKVRAFYYNPASKKCEEFTYGGCEGNANNFKTKDECHYTCVXXXXXXXXx  91 79%
Q2ES47        1    MSSGGLLLLLGLLTLWAELTPISGQDRPKFCHLPVDSGICRAHIPRFYYNPASNQCQGFIYGGCGGNANNFETRDQCRHTCGGK-------  84
CAL69605      1    MSSGGLLLLLGLLTLWAELTPISGHDRPKFCYLPADPGECMAYIRSFYYDSESKKCKEFIYGGCHGNANNFPTRDKCRQTCRAPRKGRHT-  90
CAL69613      1    MSSGGLLLLLGLLTLWAELTPISGHDRPKFCYLPADPGECLAHMRSFYYDSESKKCKEFIYGGCHGNANKFPSRDKCRQTCGASAKGRPT-  90
```

IX. VNGF

Accession	Description	Links
lcl|Query_80281	NGF	
AEH59580.1	venom nerve growth factor 2 [Daboia russelii]	

```
Query_80281  1   MSMLCYTLIIAFLIGIRAAPKSEDNVSLRSPATPDLSDTSCAKTHEALKTSRNTDQHYPAPNKAEDQEFGSAANIIVDPKLFQKRRFQSPRVLFSTQPPPLSRDEQSVEFLDNADSLNRNIRAKRATHPVHNQGEFSVCDSVSVWVANKTTATDMRGNVVTVMVDVNLNNNVYKQYFFETKCKNPNPVPSGCRGIDAKHWNSYCTTTDTFVRALTMERNQASWRFIRINTACVCVISRKNDNFGXALX  248 100%
AEH59580     1   MSMLCYTLIIAFLIGIRAAPKSEDNVSLRSPATPDLSDTSCAKTHEALKTSRNTDQHYPAPNKAEDQEFGSAANIIVDPKLFQKRRFQSPRVLFSTQPPPLSRDEQSVEFLDNADSLNRNIRAKRATHPVHNQGEFSVCDSVSVWVANKTTATDMRGNVVTVMVDVNLNNNVYKQYFFETKCKNPNPVPSGCRGIDAKHWNSYCTTTDTFVRALTMERNQASWRFIRINTACVCVISRKNDNF-----  243
```

X. VEGF

Accession	Description	Links
C0K3N5.1	RecName: Full=Vascular endothelial growth factor A; Flags: Precursor	Related Information
P67861.2	RecName: Full=Snake venom vascular endothelial growth factor toxin VR-1; Short=svVEGF; AltName: Full=VEGF-F; Flags: Precursor	Related Information
lcl|Query_10001	lcl|Query_121782 VEGFA1	
lcl|Query_10002	VEGFA2	
lcl|Query_10003	VEGFA3	
lcl|Query_10004	VEGFB	
lcl|Query_10005	VEGFC	
lcl|Query_10006	VEGFF	

```
Query_10001  1      XXXXXXXXXXGLAALLYFHNAKVLQAAPAQGDGDRQQGEGISFLTVYERSACRPVETMVDIFQEYPDEVEXIFKPSCVALMRCGGCCNDEALECVPTEVYNVTMEIMKLK-P-FQSQHIHPMSFQQHSKCECXXXXXXXXXXXXXXXXXXXX    XXXXXXX[9]HCEPCSERRKHLYKQDPLTCKCSCK--FTDSRCKSKQLELNERTCXXXXXXXX      217 95%
Query_10002  1      XXXXXXXXXXGLAALLYFHNAKVLQAAPAQGDGDRQQGEGISFLTVYERSACRPVETMVDIFQEYPDEVEXIFKPSCVALMRCGGCCNDEALECVPTEVYNVTMEIMKLK-P-FQSQHIHPMSFQQHSKCECXXXXXXXXXXXX--------    -------   HCEPCSERRKHLYKQDPLTCKCSCK--FTDSRCKSKQLELNERTCXXXXXXXX      193 99%
Query_10003  1      XXXXXXXXXXGLAALLYFHNAKVLQAAPAQGDGDRQQGEGISFLTVYERSACRPVETMVDIFQEYPDEVEXIFKPSCVALMRCGGCCNDEALECVPTEVYNVTMEIMKLK-P-FQSQHIHPMSFQQHSKCECXXXXXXXXXXXXXXXXXXX-    -------   -----------------------------------------------------          99%
Query_10004  1      -------------------------XXXSQLRARXVSPTVIRFLEVYNRSVCQPKETMVSVTAEHPSLGSHIVIPSCVALSRCAGCCSDDSLHCVPSRSREVILEVM-AS-F-FPNRYITQLTFEEHLECMCRXXXXXXXXXXXXXXXXXXX    XXXK---   -------------QPDPDTCRCVCR--HRSGHCERRGMKFSEKTCXXXXXXXX[29] 195 65%
Query_10005  1  [96]NFDGRTGDSNPIKFAAAHYNTEILKSIDNE---------------WRKTQCMPREVCVDVGKESGATTNIFFKPPCVSIYRCGGCCNSEGLQCMNISASYVSKTLFEITvPlSHGPKPVTISFANHTSCRCMSKLDVYRQVHSIIRRSLPV[25]LAQHDFS[9]TAEGYHDICGSNKELDEETCQCVCKggVTPSSCGIHK-ELDRTSCQCTCKNKL[97] 422 28%
Query_10006  1      --------------XXXXXXXXXXXXXXXXXXXXXXXXXXXXXXXXXXXXXXXXXXXXXXXSHIFKPSCVILWXCASCCNDDSLKCXXXXXXXXXXXXXXXX-X-XXXXXXXXXXXXXXXXXXXXXXXXXXXXXXXXXXXXXXX    XXXXXXX   -----------------------------------------------------      143 97%
C0K3N5       1      MNFLLSWIHWGLAALLYFHNAKVLQAAPAQGDGDRQQGEVISFLTVYERSACRPVETMVDIFQEYPDEVEYIFKPSCVALMRCGGCCNDEALECVPTEVYNVTMEIMKLK-P-FQSQHIHPMSFQQHSKCECRPKKEVRIRQEN--------    -------   HCEPCSERRKHLYKQDPLTCKCSCK--FTDSRCKSKQLELNERTCRCEKPRR-      192
P67861       1      --------------MAAYLLAVAILFCIQGWPSGTVQGQVRPFLDVYERSACQTRETLVSILQEHPDEISDIFRPSCVAVLRCSGCCTDESMKCTPVGKHTADIQIMRMN-PrTHSSKMEVMKFMEHTACECRPRWKQGEPEGPKEPRRGGV    RAKFPFD   -----------------------------------------------------      144
```

Fig. S4: Sequence similarity between proteins belonging to eight major venom families and their blood homologs in king cobra, *Ophiophagus hannah.* The homolog with the highest identity was considered, in case of more than one homolog for a venom family.

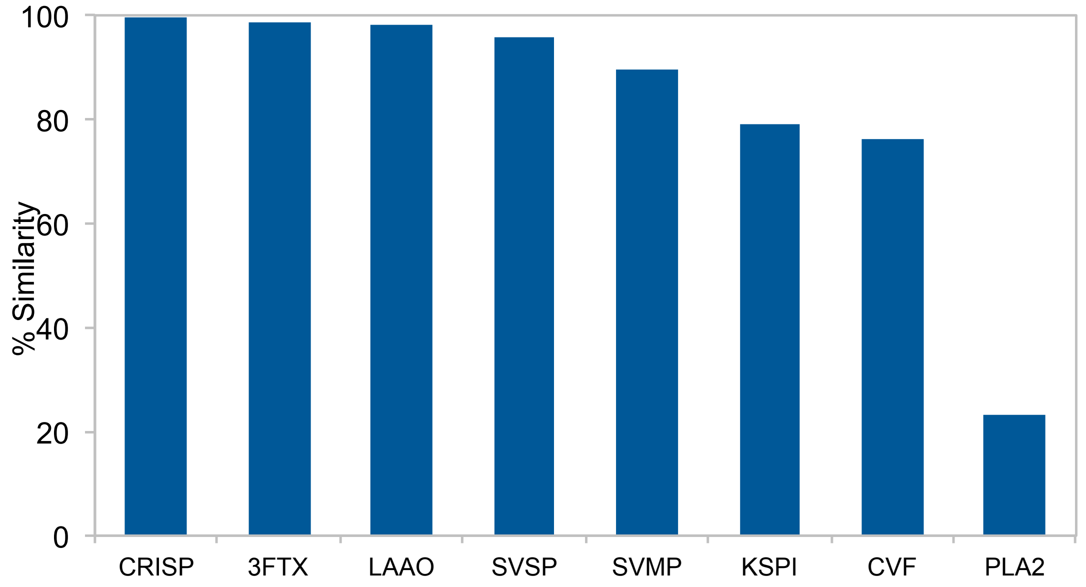

Fig. S5: Comparison of eight toxin family protein sequences from venom-associated proteins from venom gland of king cobra *Ophiophagus hannah* with the putative venom protein homologs from the predicted genome sequencing, assembly and annotation of *Ophiophagus hannah* blood (PRJNA201683; Vonk *et al.* 2013). Additional file 4 from Tan *et al*. 2015 was used as a reference for procuring the accession ID of the venom/venom gland associated protein from *Ophiophagus hannah*. Comparative analyses were performed using blastp, with the venom/venom gland associated protein sequence as the query, against PRJNA201683. In the multiple sequence alignments, the topmost sequence is from the venom/venom gland, and the remaining sequences are from blood. Red indicates an identical match in comparison in at least one of the predicted protein sequence. Blue indicates that amino acid is variable in all the sequences used for comparison. Light gray indicates non-identical amino acid.

I. 3FTX (three finger toxin family)

Accession	Description	Links
Q53B59. 1	RecName: Full=Long neurotoxin OH-37; Flags: Precursor	Related Information
ETE56610.1	hypothetical protein L345_17679 [Ophiophagus hannah]	
ETE57194.1	hypothetical protein L345_17093 [Ophiophagus hannah]	
ETE56824.1	hypothetical protein L345_17464 [Ophiophagus hannah]	
ETE58964.1	hypothetical protein L345_15308 [Ophiophagus hannah]	

```
Q53B59     1   MKTLLLTLVVMTIVCLDLGYSLICFIS----PHDSVTCAPGENVCFLKSWCDAwcgSRGKKLSFGCAATCPKVNPGIDIECCSTDNCNPhpklrp  91
ETE56610   1   -------------------YTRICHKS----SFISETCPDGQNLCYLKSWCDIfcgSRGERLEFGCAATCPEVKPGVNIECCSTDNCNPhpklrp  72
ETE57194   1   MKNLLLTFLVVTIVCLDLGYTLICHRV-----HGLQTCEPDEKFCFRKTTMFF---PNHPVLLMGCTYSCPTAK---YSVCCSTDKCNK------  78
ETE56824   1   MKTVLLTLVVVTIVCLDLGHTLICVKQytifGVTPEICADGQNLCYKTWHMVY---PGGYDHTRGCAATCPKMKNHDTVHCCTTDKCNL------  86
ETE58964   1   MKTLLLTLVVVTIVCLDLGHTRICLTDyskvSETIEICPDGQNFCFKKFPKGI---PFLPWVNRGCAATCPKPEPKVYVDCCARDKCNR------  86
```

Accession	Description	Links
Q2VBP8.1	RecName: Full=Long neurotoxin LNTX1; Flags: Precursor	Related Information
ETE56610.1	hypothetical protein L345_17679 [Ophiophagus hannah]	
ETE57194.1	hypothetical protein L345_17093 [Ophiophagus hannah]	
ETE56824.1	hypothetical protein L345_17464 [Ophiophagus hannah]	
ETE58964.1	hypothetical protein L345_15308 [Ophiophagus hannah]	

```
Q2VBP8     1   MKILLLTLVVVTIMCLDLGYTTKCYKTGERI--ISETCPPGQDLCYMKTWCDVfcgSRGRVIELGCTATCPTVKPHEQITCCSTDNCNPhpkmkqr  94
ETE56610   1   -------------------YTRICHKSS--F--ISETCPDGQNLCYLKSWCDIfcgSRGERLEFGCAATCPEVKPGVNIECCSTDNCNPhpklrp-  72
ETE57194   1   MKNLLLTFLVVTIVCLDLGYTLICHRVHGL-----QTCEPDEKFCFRKTTMFF---PNHPVLLMGCTYSCPTAK---YSVCCSTDKCNK-------  78
ETE56824   1   MKTVLLTLVVVTIVCLDLGHTLICVKQYTIFgvTPEICADGQNLCYKTWHMVY---PGGYDHTRGCAATCPKMKNHDTVHCCTTDKCNL-------  86
ETE58964   1   MKTLLLTLVVVTIVCLDLGHTRICLTDYSKVseTIEICPDGQNFCFKKFPKGI---PFLPWVNRGCAATCPKPEPKVYVDCCARDKCNR-------  86
```

Accession	Description	Links
P84716.1	RecName: Full=Neurotoxin-like protein 1	
ETE58964.1	hypothetical protein L345_15308 [Ophiophagus hannah]	
ETE56824.1	hypothetical protein L345_17464 [Ophiophagus hannah]	
ETE56610.1	hypothetical protein L345_17679 [Ophiophagus hannah]	

```
P84716     1   ---------------------MKCLTKYSRVSETSQTCHVWQNLCFKKWQKGK-------KVSRGCTATCPKPKKDEVIQCCAKDKCNK------  61
ETE58964   1   MKTLLLTLVVVTIVCLDLGHTRICLTDYSKVSETIEICPDGQNFCFKKFPKGI---PFLPWVNRGCAATCPKPEPKVYVDCCARDKCNR------  86
ETE56824   1   MKTVLLTLVVVTIVCLDLGHTLICVKQYTIFGVTPEICADGQNLCYKTWHMVY---PGGYDHTRGCAATCPKMKNHDTVHCCTTDKCNL------  86
ETE56610   1   -------------------YTRICHKS----SFISETCPDGQNLCYLKSWCDIfcgSRGERLEFGCAATCPEVKPGVNIECCSTDNCNPhpklrp  72
```

Accession	Description	Links
Q2VBP1.1	RecName: Full=Short neurotoxin SNTX11; Flags: Precursor	Related Information
ETE57194.1	hypothetical protein L345_17093 [Ophiophagus hannah]	
ETE58964.1	hypothetical protein L345_15308 [Ophiophagus hannah]	
ETE56824.1	hypothetical protein L345_17464 [Ophiophagus hannah]	

```
Q2VBP1     1   MKTLLLTFLVVTIVCLDLGYTLICHRVHGL-----QTCEPDQKFCFRKTTMFFPNH-PVLLMGCTYSCPTEK---YSVCCSTDKCNK   78
ETE57194   1   MKNLLLTFLVVTIVCLDLGYTLICHRVHGL-----QTCEPDEKFCFRKTTMFFPNH-PVLLMGCTYSCPTAK---YSVCCSTDKCNK   78
ETE58964   1   MKTLLLTLVVVTIVCLDLGHTRICLTDYSKVSETIEICPDGQNFCFKKFPKGI-PFLPWVNRGCAATCPKPEPKVYVDCCARDKCNR   86
ETE56824   1   MKTVLLTLVVVTIVCLDLGHTLICVKQYTIFGVTPEICADGQNLCYKTWHMVY-PGGYDHTRGCAATCPKMKNHDTVHCCTTDKCNL   86
```

Accession	Description	Links
A8N286.1	RecName: Full=Haditoxin; AltName: Full=Muscarinic toxin-like protein 3 homolog; Short=MTLP-3 homolog; Flags: Precursor	Related Information
ETE56205.1	hypothetical protein L345_18084 [Ophiophagus hannah]	
ETE58964.1	hypothetical protein L345_15308 [Ophiophagus hannah]	
ETE57194.1	hypothetical protein L345_17093 [Ophiophagus hannah]	
ETE56824.1	hypothetical protein L345_17464 [Ophiophagus hannah]	
ETE56610.1	hypothetical protein L345_17679 [Ophiophagus hannah]	

```
A8N286     1   MKTLLLTLVVVTIVYLDLGYTTKCYNHQSTTPETTEICPDSGYFCYKSSWIDGR----------------EGRIERGCTFTCPELTPnGKYVYCCRRDKCNQ----------------------------------   86
ETE56205   1   -------------------YTTKCYNHQSTTPETTEICPDSGYFCYKSSWIDGRvfflleptlelpnaPENWRSRRTCL--CPAWVPlRSHPVARQSKQCKErgswtvvprlsqqqprlegetffrgrgremlgel  115
ETE58964   1   MKTLLLTLVVVTIVCLDLGHTRICLTDYSKVSETIEICPDGQNFCF-KKFPKGI--------------PFLPWVNRGCAATCPKPEP-KVYVDCCARDKCNR----------------------------------   86
ETE57194   1   MKNLLLTFLVVTIVCLDLGYTLICHRVHGL-----QTCEPDEKFCFRKTTM----ff-----------PNHPVLLMGCTYSCPTAKY----SVCCSTDKCNK----------------------------------   78
ETE56824   1   MKTVLLTLVVVTIVCLDLGHTLICVKQYTIFGVTPEICADGQNLCY-KTWHMVY--------------PGGYDHTRGCAATCPKMKN-HDTVHCCTTDKCNL----------------------------------   86
ETE56610   1   -------------------YTRICHKSSFI----SETCPDGQNLCYLKSWCDIF------------cgSRGERLEFGCAATCPEVKP-GVNIECCSTDNCNPhpklrp----------------------------   72
```

Accession	Description	Links
Q2VBN1.1	RecName: Full=Muscarinic toxin MTX6; Flags: Precursor	Related Information
ETE56824.1	hypothetical protein L345_17464 [Ophiophagus hannah]	
ETE58964.1	hypothetical protein L345_15308 [Ophiophagus hannah]	
ETE57194.1	hypothetical protein L345_17093 [Ophiophagus hannah]	
ETE56610.1	hypothetical protein L345_17679 [Ophiophagus hannah]	
ETE56656.1	hypothetical protein L345_17632 [Ophiophagus hannah]	

```
Q2VBN1     1   MKTLLLTLVVVTILCLDLGYTLTCLTHESLFFETTETCSDGQNLCY-A-----------------------------KWFAVF--PGARRPDGGCAATCPDKVPLEIVNCCTTDKCNL------   86
ETE56824   1   MKTVLLTLVVVTIVCLDLGHTLICVKQYTIFGVTPEICADGQNLCY-K-----------------------------TWHMVY--PGGYDHTRGCAATCPKMKNHDTVHCCTTDKCNL------   86
ETE58964   1   MKTLLLTLVVVTIVCLDLGHTRICLTDYSKVSETIEICPDGQNFCF-K-----------------------------KFPKGI--PFLPWVNRGCAATCPKPEPKVYVDCCARDKCNR------   86
ETE57194   1   MKNLLLTFLVVTIVCLDLGYTLICHRVHGL-----QTCEPDEKFCFRK-----------------------------TTMF-F--PNHPVLLMGCTYSCPTAK---YSVCCSTDKCNK------   78
ETE56610   1   -------------------YTRICHKSSFI----SETCPDGQNLCYLK-----------------------------SWCDIFcgSRGERLEFGCAATCPEVKPGVNIECCSTDNCNPhpklrp   72
ETE56656   1   ---------------------MTCYTQYSLSPPTTKTCPDGQNLCYKRlelpnapenwrsrrtclchawvplrsrsiAGQSKQc-KERNKFFRGCAAACPKAEHNE------------------   84
```

Accession	Description	Links
Q53B61.1	RecName: Full=Weak neurotoxin OH-72; Flags: Precursor	Related Information
ETE58964.1	hypothetical protein L345_15308 [Ophiophagus hannah]	
ETE56824.1	hypothetical protein L345_17464 [Ophiophagus hannah]	
ETE57194.1	hypothetical protein L345_17093 [Ophiophagus hannah]	
ETE56610.1	hypothetical protein L345_17679 [Ophiophagus hannah]	

```
Q53B61     1   -----LTLVVVTIVCLDLGYTLTCLICPEEYCKRIHTCRDGENVCFKGFYEGK---QLGKQFRRGCAATCPEGKPNEIVQCCSTDECNH------   81
ETE58964   1   MKTLLLTLVVVTIVCLDLGHTRICLTDYSKVSETIEICPDGQNFCFKKFPKGI---PFLPWVNRGCAATCPKPEPKVYVDCCARDKCNR------   86
ETE56824   1   MKTVLLTLVVVTIVCLDLGHTLICVKQYTIFGVTPEICADGQNLCYKTWHMVY---PGGYDHTRGCAATCPKMKNHDTVHCCTTDKCNL------   86
ETE57194   1   MKNLLLTFLVVTIVCLDLGYTLICHRVHG-----LQTCEPDEKFCFRKTTMFF---PNHPVLLMGCTYSCPTAK---YSVCCSTDKCNK------   78
ETE56610   1   -------------------YTRICHKSSF----ISETCPDGQNLCYLKSWCDIfcgSRGERLEFGCAATCPEVKPGVNIECCSTDNCNPhpklrp   72
```

Accession	Description	Links
Q2VBN2.1	RecName: Full=Weak neurotoxin WNTX34; Flags: Precursor	Related Information
ETE58964.1	hypothetical protein L345_15308 [Ophiophagus hannah]	
ETE56824.1	hypothetical protein L345_17464 [Ophiophagus hannah]	
ETE57194.1	hypothetical protein L345_17093 [Ophiophagus hannah]	
ETE56610.1	hypothetical protein L345_17679 [Ophiophagus hannah]	

```
Q2VBN2     1   MKTLLLTLVVVTIVCLDLGYSLTCLNCPEQYCKRIHTCRNGENVCFKRFYEGK---LLCKQFRRGCAATCPEAKSREIVQCCSTDECNH------   86
ETE58964   1   MKTLLLTLVVVTIVCLDLGHTRICLTDYSKVSETIEICPDGQNFCFKKFPKGI---PFLPWVNRGCAATCPKPEPKVYVDCCARDKCNR------   86
ETE56824   1   MKTVLLTLVVVTIVCLDLGHTLICVKQYTIFGVTPEICADGQNLCYKTWHMVY---PGGYDHTRGCAATCPKMKNHDTVHCCTTDKCNL------   86
ETE57194   1   MKNLLLTFLVVTIVCLDLGYTLICHRVHG-----LQTCEPDEKFCFRKTTMFF---PNHPVLLMGCTYSCPTAK---YSVCCSTDKCNK------   78
ETE56610   1   -------------------YTRICHKSSF----ISETCPDGQNLCYLKSWCDIfcgSRGERLEFGCAATCPEVKPGVNIECCSTDNCNPhpklrp   72
```

II. SVMP (snake venom metalloproteases)

Accession	Description	Links
A3R0T9.1	RecName: Full=Zinc metalloproteinase-disintegrin-like ohanin; AltName: Full=Snake venom metalloproteinase; Short=SVMP; Flags: Precursor	Related Information
ETE62121.1	hypothetical protein L345_12124 [Ophiophagus hannah]	
ETE62122.1	hypothetical protein L345_12125 [Ophiophagus hannah]	

```
A3R0T9     1    MIQVLLVTICLVVFPYQGSSIILESGKVNDYEVVYPQKIPVLPKSKIQRREQKMYEDTMKYEFKVNGEPVVLHLERNKELFSKDYTETHYSPDGREITTSPPVEDHCYYHGYIQSDIDSTAILNACNGLKgyfrHHGEAYHIEPLKFSDSEAHAVYKYEN   160
ETE62121   1    MIQVLLVTICLVVFPYQGSSIILESGKVNDYEVVYPQKIPVLPKSKIQRREQKMYEDTMKYEFKVNGEPVVLHLERNKELFSKDYTETHYSPDGREITTSPPVEDHCYYHGYIQSDIDSTAILNACNGLK----HHGEAYHIEPLKFSDSEAHAVYKYEN   156
ETE62122   1    ----------------------------------------------------------MQYEFKVNGEPVVLHLEKNKELFSKDYSETHYSPDGREITTSPPLENR------------------------------GEGYLIEPLKLSNSEAHALFKYES   72

A3R0T9     161  IEKEDETPKICGVKHSTWESDEPIEKISQKKDFLEEK------KYLELYIVADYVMFRKYGRNVTTIRMRVFDMVNYITVVYKALNIHVALIGFEIWSLKDKFVINASTKNNLLHFSIWRSTVL--RKRNDNAQLLTGVDLNGYTLGSAYLKAMCDVLQS   312
ETE62121   157  IEKEDETPKICGVKHSTWESDEPIEKISQVSITSEE--------------------FRKYSRNVTAIRMRVFDMVNYITVVYKALNIRVALIGFEIWSLKDKFVINASTKNNLLHFSIWRSTVL--RKRNDNAQLLTGVDLNGYTLGSAYLKAMCDVLQS   294
ETE62122   73   LEKEDKTLKTCGVTNTTWKSDEPLKKTSRTSMSIEKKeylqarKYVEFYIVADNRMFRKYSRSIAAIRMRAFDIVNFINMVYKPLKVHIALIGLEIWSNKDKIEISKTAGATLSHFSSWRKTVLlkHKRNDNAQLLTDIDFTGSTVGLAYVGTMCNSLSS   232

A3R0T9     313  VGIVQDYSKSPYLVGAAMAHEIGHNLGMEHDTKTCSCMRGNCIMSPEEEGSDFPMEFSSCSLYDFQNYMLTDTPQCLINKPSNTSIIKNAVCGNYVEEEGEECDCGSPEQCENNCCEAATCKLKPGAKCAKGACCKKCQFKKAGAECRAARNECDLPEFC   472
ETE62121   295  VGIVQDYSKSPYLVGAAMAHEIGHNLGMEHDTKTCSCMRGNCIMSPEEEGSDFPMEFSSCSLYDFQNYMLTETPQCLINKPSNTSIIKNAVCGNYVEEEGEECDCGSPEQCENNCCEAATCKLKPGAKCAKGACCKKCQFKKAGAECRAARNECDLPEFC   454
ETE62122   233  TAVIQDHSTDPIAMGATMAHEMGHNFGMNHDTDLCTCKTGPCIMA-DKQGYITPQEFSSCSLQFYQNYIMNETPQCIINRP----LIKDVI---------------SP----------------------------------------------------   320

A3R0T9     473  IGQSAECPMDRFHKNGHSCQNDQGYCFRGYCPTLAKQCITLWGSDAKVAPDECFQNNTNGNEYDYCKKTNNVIIPCKPTDvkcgrlycTGGTENPSEGEKISS----DPCKASYSEIEDIGMVDHRTKCGEKMVCS---DGKCIPL--------------   611
ETE62121   455  IGQSAECPMDRFHKNGHSCQNNQGYCFRGYCPTLAKQCITLWGSDAKVAPDECFQNNTNGNEYDYCKKTNNVIIPCKPTK--------RFPVPKPNPASKMTQallvTICLVVFPYQGRLQRHEEKTKYEDTMKYEfklNGEPVVLnleknkrlfskdyt   606
ETE62122        ----------------------------------------------------------------------------------------------------------------------------------------------------------------
```

III. PLA2 (phospholipases)

Accession	Description	Links
Q9DF33.1	RecName: Full=Acidic phospholipase A2 2; Short=svPLA2; AltName: Full=APLA2-2; AltName: Full=Phosphatidylcholine 2-acylhydrolase; Flags: Precursor	Related Information
ETE72123.1	Group 10 secretory phospholipase A2 [Ophiophagus hannah]	
ETE60513.1	hypothetical protein L345_13748 [Ophiophagus hannah]	
ETE72712.1	hypothetical protein L345_01439 [Ophiophagus hannah]	
ETE56084.1	hypothetical protein L345_18206 [Ophiophagus hannah]	
ETE59137.1	Otoconin-90 [Ophiophagus hannah]	

```
Q9DF33     1    --------------------------------------------------mNPAHLLVLSAVCVSLL------GASSIPPQPlhLVQFNGMIRCTIPgSIPWWDYSDYGCYCGSGGSGTPVDELDRCCQVHDNCYTQAQqLTECS--PYSKRYSYDCSEG  102
ETE72123   1    -------------------aaeaqrlagrgeadapaeapgrdgrfpglqlmAPTLLLALLLALPGLLrtghplGKAPSRPRR-gILQLAGMIQCTTG--RTPLAYIRYGCYCGWGGFGWPKDQVDWCCFKHDCCYGKAE-EENCA--PKTRRYPWECKDS  135
ETE60513        ------------------------------------------------------------------------------------------MIQLSTV-TYGLANFTNYGCHCGPGTQGLPVDAIDRCCHSHDCCYNKVE-MYGCN--PKVLNYQFYAQRD  66
ETE72712        --------------------------------------------------------------------------------------MVSTLLCYGDRlQIPLLALNLYGCHCGTGGFGKPLDAVDRCCFLHDCCYRHTRlSLKCHNrVKWQRYKLLCKTS  74
ETE56084        -----------------------------------------------------------------------------------------------------------------------------------------------MMEtPYLKIYSYKCSER  17
ETE59137   641  rggavtqkdatvafdnselrghlfkiageepvtfsttvpeesp--------SPEESFREVCDRFSFLqw----GDNGKLKQE--FPQLGEMLYCLTN--RCPEEFELYGCFCGQEGRGHPTDELDRCCFSHQCCLEQIK-RLGCQP-EKKSRSEVVCFDH  782

Q9DF33     103  TLTCK---------------------------------------------------------------------------ADNDECAAFVCDCDRVAAICFAGA--PYNKENINID-TTTR------C--------------------------------  146
ETE72123   136  KAKCD---------------------------------------------------------------------------DIEDKCQKMACECDRDAAKCLAKA--PYNVTLLFWP-DTRCAEKGPTCpdd-----------------------------  188
ETE60513   67   KVKCV---------------------------------------------------------------------------KSRDRCEKMVCECDQKAASCFQKHlvTYNPQFRNLP-VANCRAQRPFC--------------------------------  118
ETE72712   75   ETECR---------------------------------------------------------------------------S-KSICGRTACECDKQLAECLTAA--KPQRKHSFYK-RDLCQGAKGSCpamhqnwtktlh--------------------  135
ETE56084   18   TVTCKgkyhkpqptrlsktgipfqdramqeplhnasstpfrlgaqlhngvlkrlgaashnsaafvlvmplprftisffppDDNDECGAFICNCDRVAAHCFAAS--PYNNNNYNIDlKARCQ--------------------------------------  137
ETE59137   783  TPTCVgw----------------------------------------------------------------------------TLCENLLCSCDKAAAECMAAA--PFNESWRLSG-RQACQEDKLAC-----rwagrvrppsspprgartsseeeesss  858
```

Accession	Description	Links
P80966.2	RecName: Full=Acidic phospholipase A2 1; Short=svPLA2; AltName: Full=APLA2-1; AltName: Full=OHV A-PLA2; Short=OHV-APLA2; AltName: Full=Phosphatidylcholine 2-acylhydrolase; Flags: Precursor	Related Information
ETE72123.1	Group 10 secretory phospholipase A2 [Ophiophagus hannah]	
ETE56084.1	hypothetical protein L345_18206 [Ophiophagus hannah]	
ETE60513.1	hypothetical protein L345_13748 [Ophiophagus hannah]	
ETE72712.1	hypothetical protein L345_01439 [Ophiophagus hannah]	
ETE59137.1	Otoconin-90 [Ophiophagus hannah]	

```
P80966     1    --------------------------------------------------MNPAHLLVLSAVC----VSLLGASSiPPQPLH-LIQFGNMIQCTVPGFlsWIKYADYGCYCGAGGSGTPVDKLDRCCQVHDNCYTQAQKLPACSS----imdsPYVKIYS  101
ETE72123   1    -------------------aaeaqrlagrgeadapaeapgrdgrfpglqlMAPTLLLALLLALPGLLRTGHPLGKaPSRPRRGILQLAGMIQCTTGRT--PLAYIRYGCYCGWGGFGWPKDQVDWCCFKHDCCYGKAE-EENCA---------PKTRRYP  129
ETE56084   1    -------------------------------------------------mMETPYLKIYSYKCSERTVTCKGKYH-KPQPTR-LSKTG-------------IPFQDRAMQEPLHNASSTPFRLG--AQLHNGVLKRLGAASHNSAAfvlvmplPRFTISF  94
ETE60513        -----------------------------------------------------------------------------------------MIQLSTVTY-gLANFTNYGCHCGPGTQGLPVDAIDRCCHSHDCCYNKVE-MYGCN---------PKVLNYQ  60
ETE72712        -------------------------------------------------------------------------------MVSTLLCYGDRLQI----P--LLALNLYGCHCGTGGFGKPLDAVDRCCFLHDCCYRHTRLSLKCHNR-------VKWQRYK  68
ETE59137   641  rggavtqkdatvafdnselrghlfkiageepvtfsttvpee----------SPSPEESFREVCDRFSFLQWGDNG---KLKQEFPQLGEMLYCLTNRC--PEEFELYGCFCGQEGRGHPTDELDRCCFSHQCCLEQIK-RLGCQPE--------KKSRSE  77

P80966     102  YDCSERTVTCKADNDECAAFICNCDRVAAHCFAAS--PYNNNNYNIDTtTRC----------------------------  151
ETE72123   130  WECKDSKAKCDDIEDKCQKMACECDRDAAKCLAKA--PYNVTLLFWPD-TRCAEKGPTCpdd------------------  188
ETE56084   95   FPPD--------DNDECGAFICNCDRVAAHCFAAS--PYNNNNYNIDLkARCQ---------------------------  137
ETE60513   61   FYAQRDKVKCVKSRDRCEKMVCECDQKAASCFQKHlvTYNPQFRNLPV-ANCRAQRPFC---------------------  118
ETE72712   69   LLCKTSETECR-SKSICGRTACECDKQLAECLTAA--KPQRKHSFYKR-DLCQGAKGSCpamhqnwtktlh---------  135
ETE59137   777  VVCFDHTPTCV-GWTLCENLLCSCDKAAAECMAAA--PFNESWRLSGR-QACQEDKLAC-----rwagrvrppsspprga  847
```

IV. CRISP (cysteine rich secretory proteins)

Accession	Description	Links
ACN93671.1	opharin precursor [Ophiophagus hannah]	
ETE62137.1	hypothetical protein L345_12110 [Ophiophagus hannah]	
ETE58161.1	hypothetical protein L345_16118 [Ophiophagus hannah]	

```
ACN93671   1    ------MIAFT-LLSLAAVLQQSSGTVDFASESSNKRENQKQIVDKHNALRRSVKPTARNMLQMEWNSNAAQNAKRWADRCSFAHSPPHLRAVGKFSCGENLFMSSQPYAWSRVIQSWYDENKKFVYGVGANPPGSVIGHYTQIVWYKSHLLGCAAARCS  153
ETE62137   1    myypveMIAFIfLLSLAAVLQQSSGTVDFASESSNKRENQKQIVDKHNALRRSVKPTARNMLQMEWNSNAAQNAKRWADRCSFAHSPPHLRTVGKFSCGENLFMSSQPYAWSRVIQSWYDENKKFVYGVGANPPGSVIGHYTQIVWYKSHLLGCAAARCS  160
ETE58161   1    ------MLILI------------------PSQPGDKKK-QKEIVDLHNSLRRSVSPTASNMLKMQWYPEAASNAERWASNCILGHSPDYSRVLEGIQCGENLYKSSHPHAGSRVIQSLYDEYKYFNYGVGANLPASLIGHYTQIVWYKTYRIGCAVNYCP  135

ACN93671   154  SSKY--LYVCQYCPAGNIRGSIATPYKSGPPCGDCPSACDNGLCTNPCKYKDDFSNCQSLAKQTKCQTEWIKSKCPASCFCHNKII  237
ETE62137   161  SSKY--LYVCQYCPAGNIRGSIATPYKSGPPCGDCPSACVNGLCTNPCKYKDDFSNCQSLAKQTKCQTEWIKSKCPASCFCRTEII  244
ETE58161   136  SSEYnyFYVCQYCPSGNMRGSTATPYKSGPTCGDCPSACDNGLCTNPCTLYNEYTNCDSLVKQSSCQDEWIKSKCPASCFCHNKII  221
```


Accession	Description	Links
ACN93671.1	opharin precursor [Ophiophagus hannah]	
ETE62137.1	hypothetical protein L345_12110 [Ophiophagus hannah]	
ETE58161.1	hypothetical protein L345_16118 [Ophiophagus hannah]	

```
ACN93671   1    ------MIAFT-LLSLAAVLQQSSGTVDFASESSNKRENQKQIVDKHNALRRSVKPTARNMLQMEWNSNAAQNAKRWADRCSFAHSPPHLRAVGKFSCGENLFMSSQPYAWSRVIQSWYDENKKFVYGVGANPPGSVIGHYTQIVWYKSHLLGCAAARCS  153
ETE62137   1    myypveMIAFIfLLSLAAVLQQSSGTVDFASESSNKRENQKQIVDKHNALRRSVKPTARNMLQMEWNSNAAQNAKRWADRCSFAHSPPHLRTVGKFSCGENLFMSSQPYAWSRVIQSWYDENKKFVYGVGANPPGSVIGHYTQIVWYKSHLLGCAAARCS  160
ETE58161   1    ------MLILI------------------PSQPGDKKK-QKEIVDLHNSLRRSVSPTASNMLKMQWYPEAASNAERWASNCILGHSPDYSRVLEGIQCGENLYKSSHPHAGSRVIQSLYDEYKYFNYGVGANLPASLIGHYTQIVWYKTYRIGCAVNYCP  135

ACN93671   154  SSKY--LYVCQYCPAGNIRGSIATPYKSGPPCGDCPSACDNGLCTNPCKYKDDFSNCQSLAKQTKCQTEWIKSKCPASCFCHNKII  237
ETE62137   161  SSKY--LYVCQYCPAGNIRGSIATPYKSGPPCGDCPSACVNGLCTNPCKYKDDFSNCQSLAKQTKCQTEWIKSKCPASCFCRTEII  244
ETE58161   136  SSEYnyFYVCQYCPSGNMRGSTATPYKSGPTCGDCPSACDNGLCTNPCTLYNEYTNCDSLVKQSSCQDEWIKSKCPASCFCHNKII  221
```

V. KUN (kunitoxin)

Accession	Description	Links
B6RLX2.1	RecName: Full=Kunitz-type serine protease inhibitor TCI; AltName: Full=Trypsin and chymotrypsin bi-functional serine protease inhibitor; Short=OH-TCI; Flags: Precursor	Related Information
ETE57835.1	hypothetical protein L345_16446 [Ophiophagus hannah]	
ETE56995.1	hypothetical protein L345_17293 [Ophiophagus hannah]	

```
B6RLX2     1     MSSGRLLLLLGLLTLWAELTPVSGLGRPKFCELPAVSGFCKAYIPSFYYNPDASACQKFIYGGCGGNANKFKTIEECHRTCVG   83
ETE57835   1     MASGGLLLLLGLLTLWAELTPVSGQDCPKFCYLPAETGPCKAKMPRFYYNPASKQCEKFTYGGCKGNDNNFKTLDQCRYTCV-   82
ETE56995   1     MSSGGLLLLLGLLTLWAELTPVSSQDRPGFCLLRPDRGPCEGNIRAFYYSPSSNSCQEFIYGGCQGNANRFKTNNECHHTCVE   83
```

VI. LAAO (L amino acid oxidases)

Accession	Description	Links
P81383.3	RecName: Full=L-amino-acid oxidase; Short=LAAO; Short=LAO; Short=Oh-LAAO; Flags: Precursor	Related Information
ETE56914.1	hypothetical protein L345_17374 [Ophiophagus hannah]	
ETE59524.1	hypothetical protein L345_14746 [Ophiophagus hannah]	

```
P81383    1    MNDF-LLLLLVLFLGVPRSENHVINLEE---CFQEPEYENWLATASHGLTKTLNPKKIVIVGAGISGLTAAKLFREAGHEVVILEASDRVGGRIKTHREDGWYVDVGPMRVPQTHRIVREYIKKFNISLNPFRQTDENAWYLIKHVRQKMSA--NNPENF  154
ETE56914  1    L--FlVLLLLVLFLGVPRSENHVINLEE---CFQEPEYENWLATASHGLTKTLNPKKIVIVGAGISGLTAAKLFREAGHEVVILEASDRVGGRIKTHREDGWYVDVGPMRVPKTHRIVREYIKKFNISLNPFRQTDENAWYLIKHVRQKMSA--NNPENF  153
ETE59524  1    MTIW-------------VSENHANEQEDwmkCFEDPNYEELLNIARNGLEKTSEPKKIVIVGAGISGLTAAKLLKDAGHQVHILEASNRIGGRINTHREKDWYVELGAMRLPKAHRICREYIKKFNLSLNPFVLSNKNAWYLFRNRRERVSAvnENPNVF  147

P81383    155  GYQLNPNERGKSASQLFDETLDKVTDDCTLQKEKYDSFSTKEYLIKEGKLSTGAVEMIGDFLNEEAGFHNSFLISVMDHFLFL---------NNSFDEITGGFDQLPERFFKDMDSIVHLNSTVEKIVHINNKVTVFY--EGLSTNMRLVADYVLITATA  303
ETE56914  154  GYQLNPNERGKSASQLFDETLDKVVLR-SRRDVQADKSNALEYLIKEGKLSTGAVEMIGDFLNEEAGFHNSFLISVMDHFLFL---------NNSFDEITGGFDQLPESFFKDMDSIVHLNSTVEKIVHINNKVTVFY--EGLSTNMRLVADYVLITATA  301
ETE59524  148  GYPMRNTELGKSAEELYKSTLDRTTKNCTRLKMKYDSYSTKEYLIKEGNLSSGAV--------AMIGDLFNKGVEYYVSFFTSvlnyitfsdENGFEEITGGFDQLPNSFYQEMSRIIHLNSRVEKIIRSGKKVKVFFhkEDEDDSSLLIVDYVLVTATA  299

P81383    304  RATRLIKFVPPLSIPKTRALRSLIYASATKIILVCTDKFWEKDGIHGGRSITDLPSRVIYYPNHDFTNGIGVLLASYTWYSDSEFYTTLSDEKCVDVVMDDLVEIHNVSKDYLKSVCGKHVVQKWALDQYSMGAFSTYTPYQITHYSQMLAQNEGRIYFA  463
ETE56914  302  RATRLIKFVPPLSIPKTRALRSLIYASATKIILVCTDKFWEKDGIHGGRSITDLPSRVIYYPNHDFTNGIGVLLASYTWYSDSEFYTTLSDEKCVDVVMDDLVEIHKVSKDYLKSVCGKHVVQKWALDQYSMGAFSTYTPYQITHYSQMLAQNEGRIYFA  461
ETE59524  300  KATRLIKFQPPLSHLKAYALYSLHYTSATKVVLVCTDKFWEREGIRGGVSMTDNPTRLVSYPSHDFPGGLGVLLVSYTVDDDADFFVPLSDDECLDVVMNDLSKIHNISKIYLESVCNRHVIQKWALDKFSMGAFSFPTPYQYSFFIKALFQNEGRVYFA  459

P81383    464  GEYTAHPHGWIETSMKSAIREAINIH-NA-------------  491
ETE56914  462  GEYTAHPHGWIETSMKSAIREAINIH-NA-------------  489
ETE59524  460  GEHTTYPFAWIDSAMKSAIRAASNIHlNAhksisqelkesaw  501
```

VII. CVF (cobra venom factor)

Accession	Description	Links
AFJ59923.1	OVF precursor protein [Ophiophagus hannah]	Related Information
ETE59418.1	Complement C3 [Ophiophagus hannah]	
ETE58564.1	Cobra venom factor [Ophiophagus hannah]	

```
AFJ59923  1     [908]LKV-VPEGVQKCIVTIIKLDPRAKGVDGTQREVVKARKLDDKVPDTEIETKITIQADPVAQIIENSIDGSKLNHLIITPSGCGEQNMIRMTAPVIATYYLDTTEQWETLGRNHRNEAVKQIMTGYAQQMVYKKA[10]ASSTWLTAYVVKVFAMA  1068
ETE59418  1          MDIgKPEGEQKNIVTIIELDPSVKGIGGTQEQTVTANKLDDKVPETEIETKISVLGDPVAQVIEDSIDGSKLKHLIITPSGCGEQNMITMTPSVIATYYLDATGQWENLGVDRRTEAVKQIMKGIQDSKLQLLL    LLLLRLTAYVVKVFAMA  151
ETE58564                 --------------------------------------------------------------------------------------------------------------------------------------    -----LTAYVVKIFALA  12

AFJ59923  1069  TKMVAGISHEIICGGVRWLILNRQQPDGAFKENAPVLSGTMQGGIQGDESEVTVTAFTLVALLESKTICNDSVNSLDSSIKKATDYLLKKYEKLQRPYTTALTAYALAAADRLNDDR[9]NRWEEYNAHTHNVEGTSYALLALLKMKKFDQTGPIVRWLT  1234
ETE59418  152   AKMVKDISHEIICGGVKWLILNRQQPDGVFKENAPVIHGEMLGGTKGAEPEASLTAFVMAALLESRSVCNEHINILDSSINKATDYLLKKYEKLQRPYTTALTAYALAAADRLNDDR   ----------------------------------------  268
ETE58564  13    AKTVKDISHEIICGGVRWLILSRQQPDGVFKENAPVLSGTMQGGIQGAEPEVCLTGFTLVALLESRSICNEYINVRD---------------------------VVMGSVKSHHQER   NCWEEYNAHTHNIEGTSYALLALLKMKKFVEAGPVVKWLI  142

AFJ59923  1235  DQNFYGGTYGQT    QATVMLFQALAEYKIQMPTHKDLNLDIIIKLPERELPLHYRLDATNAILARTAETKLNQDFTVSASGDGTATMTILTVYNAQLQEKANVCNKFHLDVSVENIHLNFKHAKGAKGALMLKICMRYLGEVDSTMTIIDISMLTGFL  1390
ETE59418  269   ------------    -ATVMVFQALAEYEIQMPTHKDLNLDIAIKLPERELPVRYRINYENALLARTTETKLNEDFTVSASGDGKATMTILTVYNAQLREDANVCNKFHLDVSVENVQLNLKQAKGAKGALKLKICTRYLGEVDSTMTIIDVSMLTGFF  411
ETE58564  143   DQKYYGGTYGQT[27]QATVMVFQALAEYEIQMPTHKDLNLDIVIKLPERNLPLRYRLDATNAILARTAEVQYFSKY--------------------------------------------LKQAKGAKGALKLKICTRYLGEVDSTMTIIDVSLLTGFV  281

AFJ59923  1391  PDAEDLTRLSEGVDRYISRYEVDNNMAQKVAVIIYLDK[41]FYHPDKGTGLLNKICVGNICRCAAETCSLLSQQEKIDLPLRIQKACASNVDYVYKTKLLRIEEKDGYDIYVMDVLEVIKPGTDENPQANARQYISQRKCQEALNLNVNDDYLIWGLRS  1587
ETE59418  412   PDAEDLTRLSKGVDRYISKFEIDNNMAQKGTVVIYLDK    FYHPDKGTGLLNKICHGNICRCAEETCSLLNQQKKIDLQLRIQKACAPNVDYVYKTKLLRIEEKDGNDIYVMDVLEVIKPGTDRNPQAKARQYVSQRKCQEALNLKLDNDYLIWGLSS  567
ETE58564  282   PDAEDLMKLSKGVDRYISKFEINNNMAQKGTVIIYLD-    ----------------------------------------------------------------------------------------------------------------------

AFJ59923  1588  DLWPMKDKFSYLITKNTWIERWPHEDECQDEEFQNLCLDFAHLSNILTIFGCPT  1641
ETE59418  568   DLWPKKDDISYLITKNTWIERWPSEDECQDEEFQNLCDDFAQLSNTLTIFGCPT  621
ETE58564        ------------------------------------------------------
```

VIII. SVSP (snake venom serine proteases)

Accession	Description	Links
A8QL56.1	RecName: Full=Alpha- and beta-fibrinogenase OhS1; AltName: Full=Snake venom serine protease; Short=SVSP; Flags: Precursor	Related Information
ETE72364.1	hypothetical protein L345_01814 [Ophiophagus hannah]	
ETE66745.1	hypothetical protein L345_07470 [Ophiophagus hannah]	

```
A8QL56     1    --------------------------------------------MALIRVLASLLILQLSYAV--------TPF-DRIIGGFECNEYEHRSLVHLYNSSGFFCSGTLLNHEWVLTAAHCNRDDIQIKLGVHNVSVNYEDEQIRVPKEKLCCHSTNNCTQ  106
ETE72364   1    --------------------------------------------MAMIRVLASLLILQLSYSKSLdngakeTPIiDLIMGGSECYKSEHPFLVYLYNSAGFFCSGTLLNHEWVLTAAHCNRKDIQIKLGIHNVHVHYEDEQIRVPKEKLCCLSTKNCTQ  115
ETE66745   1    ivykpedsarlcivqhldlqnkkdptfcppthpntfdddddndddDTSSNPMFSQSPISCCYGKAI------IPF-DRIIGGFECNKNEHRSLVYLYNSAGXQGFSA-----------------------------------------------------  100

A8QL56     107  LGQDIMLIRLNSSVNYSEHIAPLSLPSNRPSMGSVCRVMGWGLLTSPEVTFPKVPHCVDINILHIQVCQAAYPSMSENYLLCAGVLEGGKDSCKGDSGGPLICNREIQGIVSWGGFPCAQLLEPGVYTKVFDYIDWIEGIIAGNTSVTCPSDNF  260
ETE72364   116  WSQDIMLIRLNSSVKNSEHIGPLSLPSSPPNMSSVCSVMGWGTITSPEVTYPEVPYCVNIQILHNLVCQAAYPTMSGKNILCAGALEGGKDSCKGDSGGPLICNEQIQGIVSWGRFPCAQLLEPGVYTKVFDYIDWIEGIIAGNTSVICP----  265
ETE66745   101  -------------------------------QGPCSTTNGCSPLHTATVTFPKVPHCVDINILHIQVCQAAYPSMSENYLLCAGVLEGGKDSCKGDSGGPLICNRQIQGVVSWGGFPCAQLLEPGVYTKVFDYIDWIEGIIAGNTSVTCPSDNF  223
```

Fig. S6: Graphical summary of comparative domain analysis performed for all toxin-associated genes across 18 completely sequenced snake species.

A *ACHE*

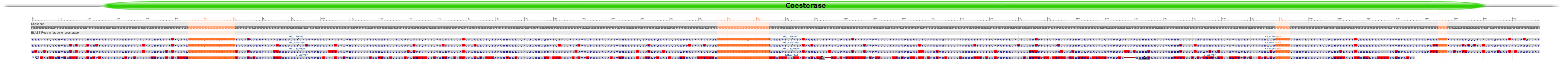

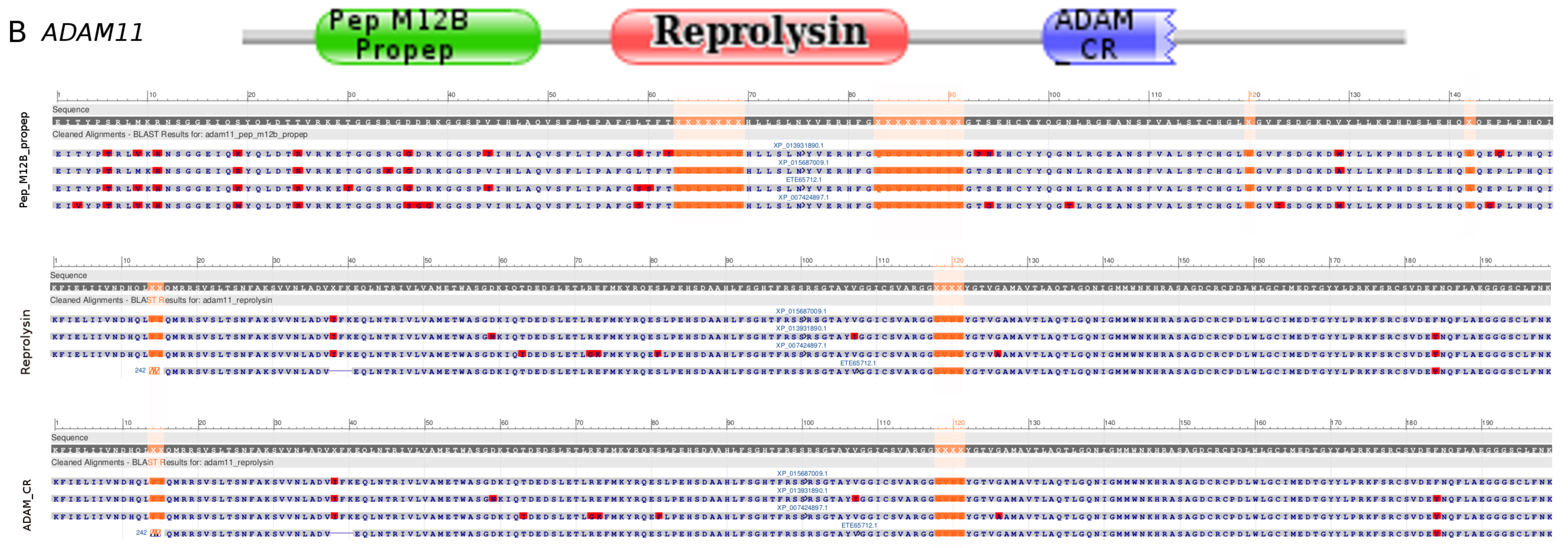
B ADAM11
Pep M12B Propep
Reprolysin
ADAM CR
Pep_M12B_propep
Sequence
Cleaned Alignments - BLAST Results for: adam11_pep_m12b_propep
XP_013931890.1
XP_015687009.1
ETE65712.1
XP_007424897.1
Reprolysin
Sequence
Cleaned Alignments - BLAST Results for: adam11_reprolysin
XP_015687009.1
XP_013931890.1
XP_007424897.1
ETE65712.1
ADAM_CR
Sequence
Cleaned Alignments - BLAST Results for: adam11_reprolysin
XP_015687009.1
XP_013931890.1
XP_007424897.1
ETE65712.1

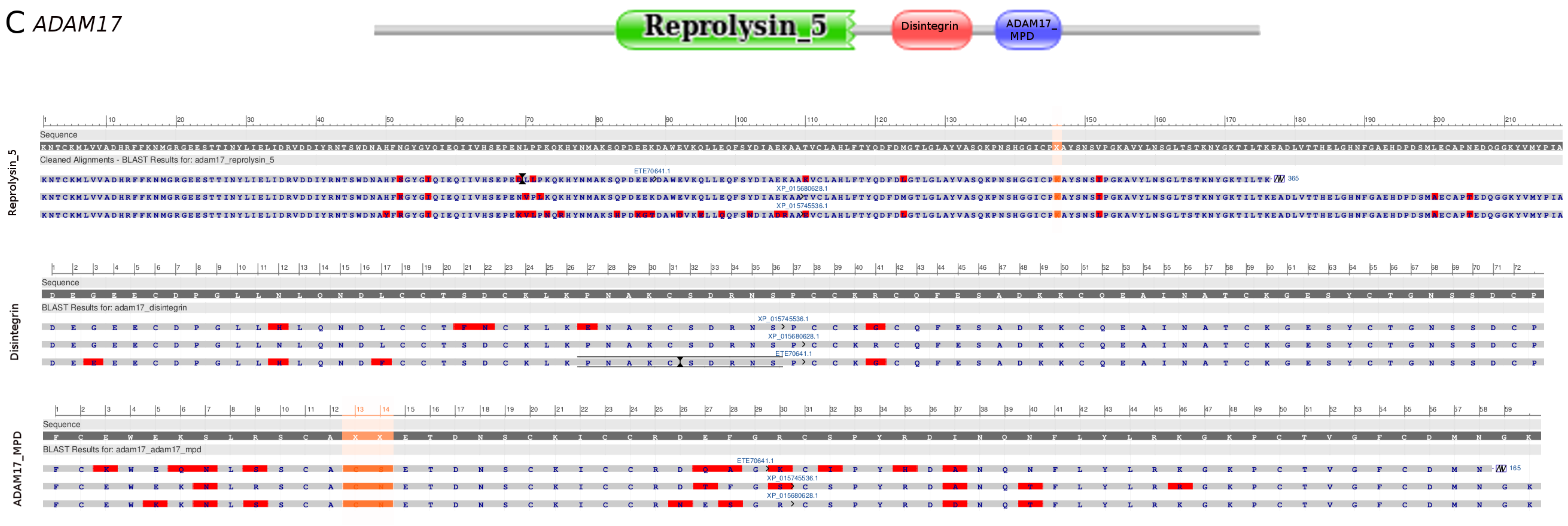

C ADAM17
Reprolysin_5
Disintegrin
ADAM17_MPD
Sequence
Cleaned Alignments - BLAST Results for: adam17_reprolysin_5
BLAST Results for: adam17_disintegrin
BLAST Results for: adam17_adam17_mpd
ETE70641.1
XP_015680628.1
XP_015745536.1

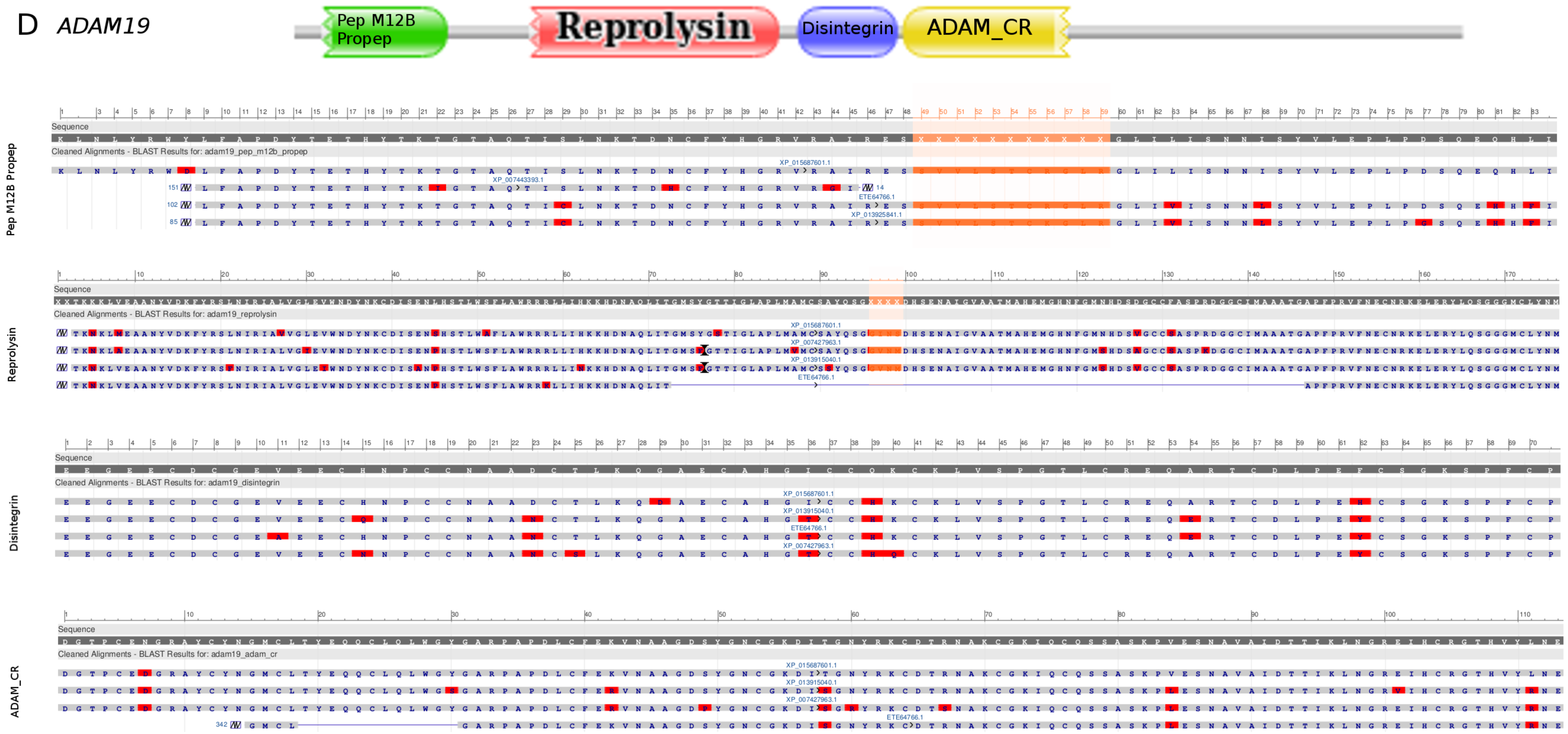
D ADAM19
Pep M12B Propep
Reprolysin
Disintegrin
ADAM_CR
Sequence
Cleaned Alignments - BLAST Results for: adam19_pep_m12b_propep
Cleaned Alignments - BLAST Results for: adam19_reprolysin
Cleaned Alignments - BLAST Results for: adam19_disintegrin
Cleaned Alignments - BLAST Results for: adam19_adam_cr
XP_015687601.1
XP_007443393.1
ETE64766.1
XP_013925841.1
XP_007427963.1
XP_013915040.1
Pep M12B Propep
Reprolysin
Disintegrin
ADAM_CR

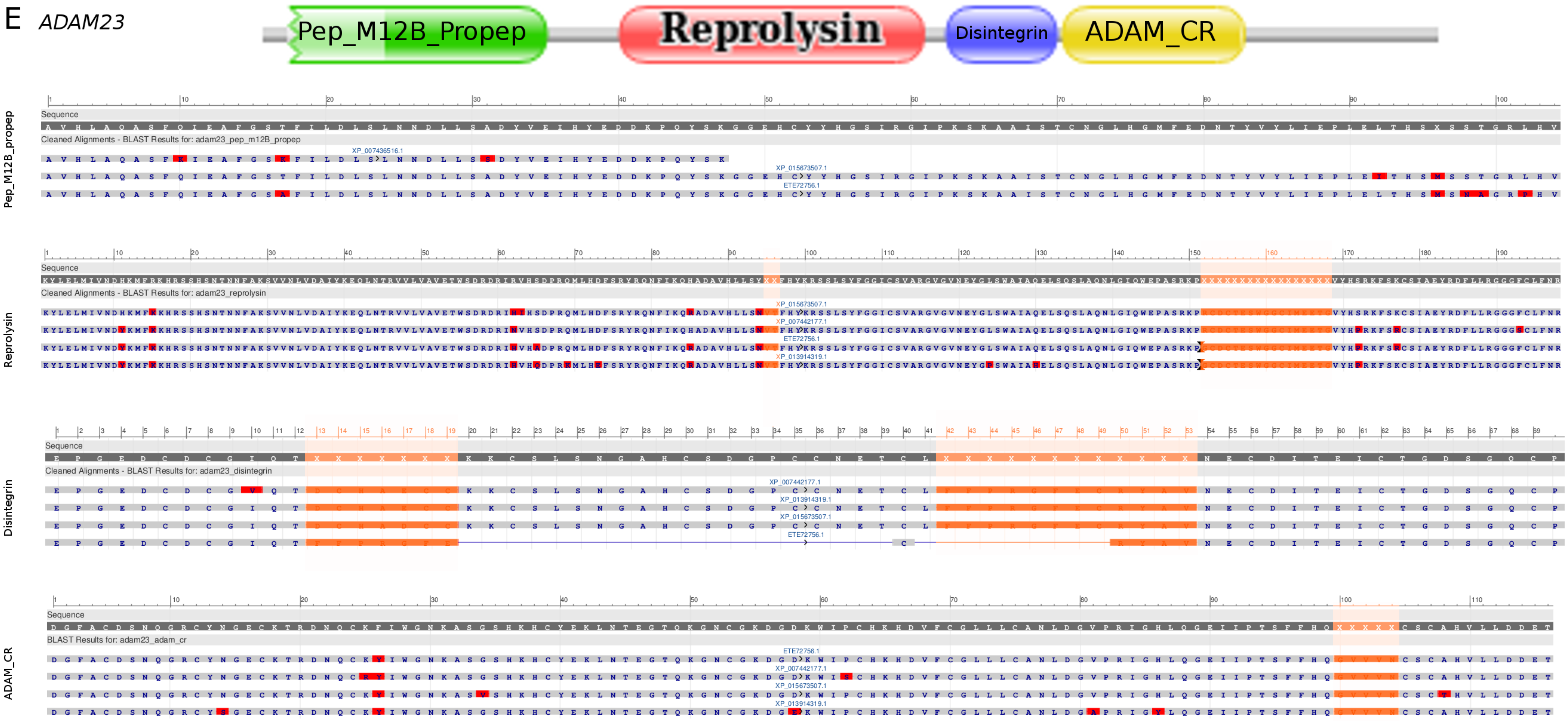
E
ADAM23
Pep_M12B_Propep
Reprolysin
Disintegrin
ADAM_CR
Pep_M12B_propep
Sequence
Cleaned Alignments - BLAST Results for: adam23_pep_m12B_propep
XP_007436516.1
XP_015673507.1
ETE72756.1
Reprolysin
Sequence
Cleaned Alignments - BLAST Results for: adam23_reprolysin
XP_015673507.1
XP_007442177.1
ETE72756.1
XP_013914319.1
Disintegrin
Sequence
Cleaned Alignments - BLAST Results for: adam23_disintegrin
XP_007442177.1
XP_013914319.1
XP_015673507.1
ETE72756.1
ADAM_CR
Sequence
BLAST Results for: adam23_adam_cr
ETE72756.1
XP_007442177.1
XP_015673507.1
XP_013914319.1

F *CLEC3A*

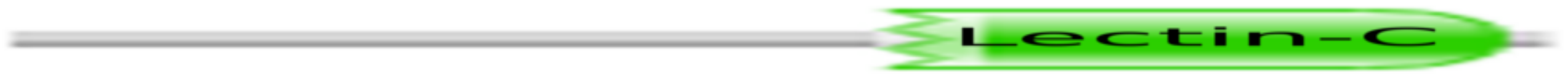
Lectin-C

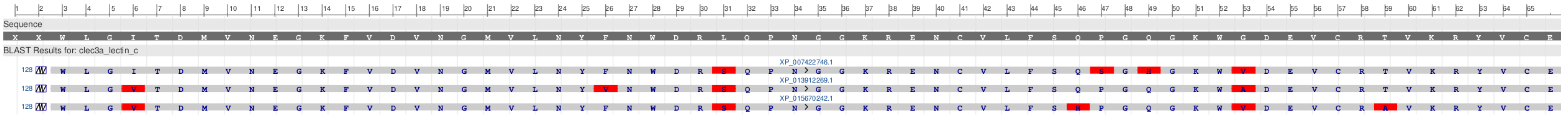
Sequence
BLAST Results for: clec3a_lectin_c
XP_007422746.1
XP_013912269.1
XP_015670242.1

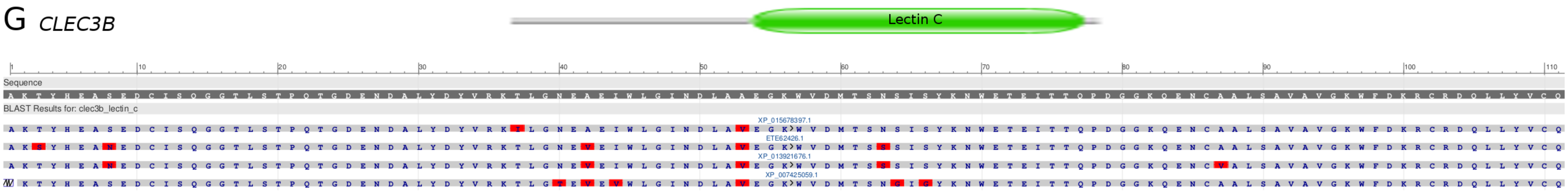
G
CLEC3B
Lectin C
Sequence
BLAST Results for: clec3b_lectin_c
XP_015678397.1
ETE62426.1
XP_013921676.1
XP_007425059.1

H *CLEC11A*

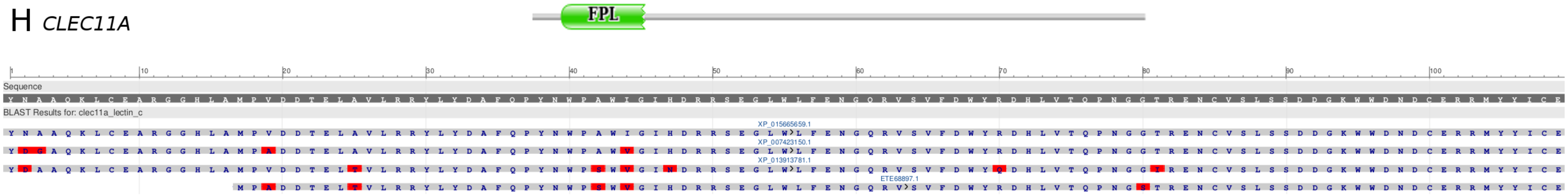
FPL
Sequence
BLAST Results for: clec11a_lectin_c
XP_015665659.1
XP_007423150.1
XP_013913781.1
ETE68897.1

CLEC16A

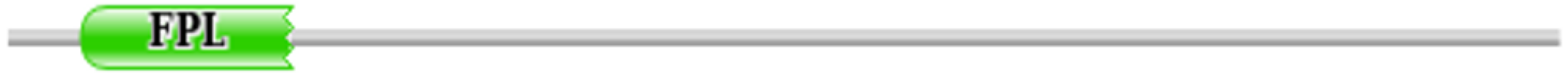
FPL

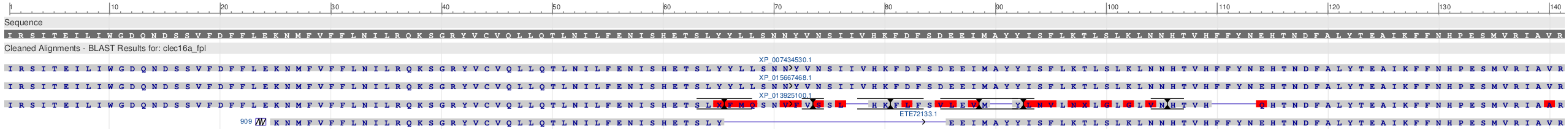
Sequence
Cleaned Alignments - BLAST Results for: clec16a_fpl
XP_007434530.1
XP_015667468.1
XP_013925100.1
ETE72133.1
909

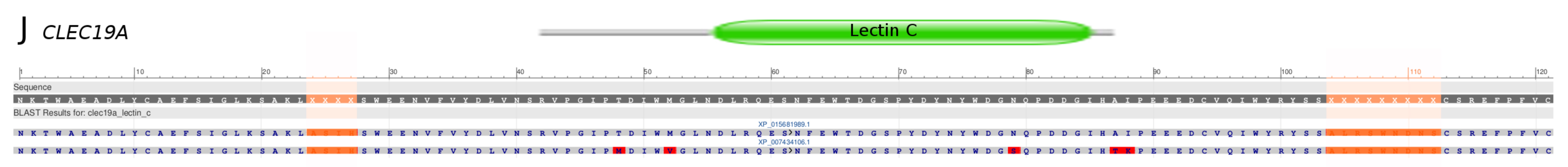
J
CLEC19A
Lectin C
Sequence
BLAST Results for: clec19a_lectin_c
XP_015681989.1
XP_007434106.1

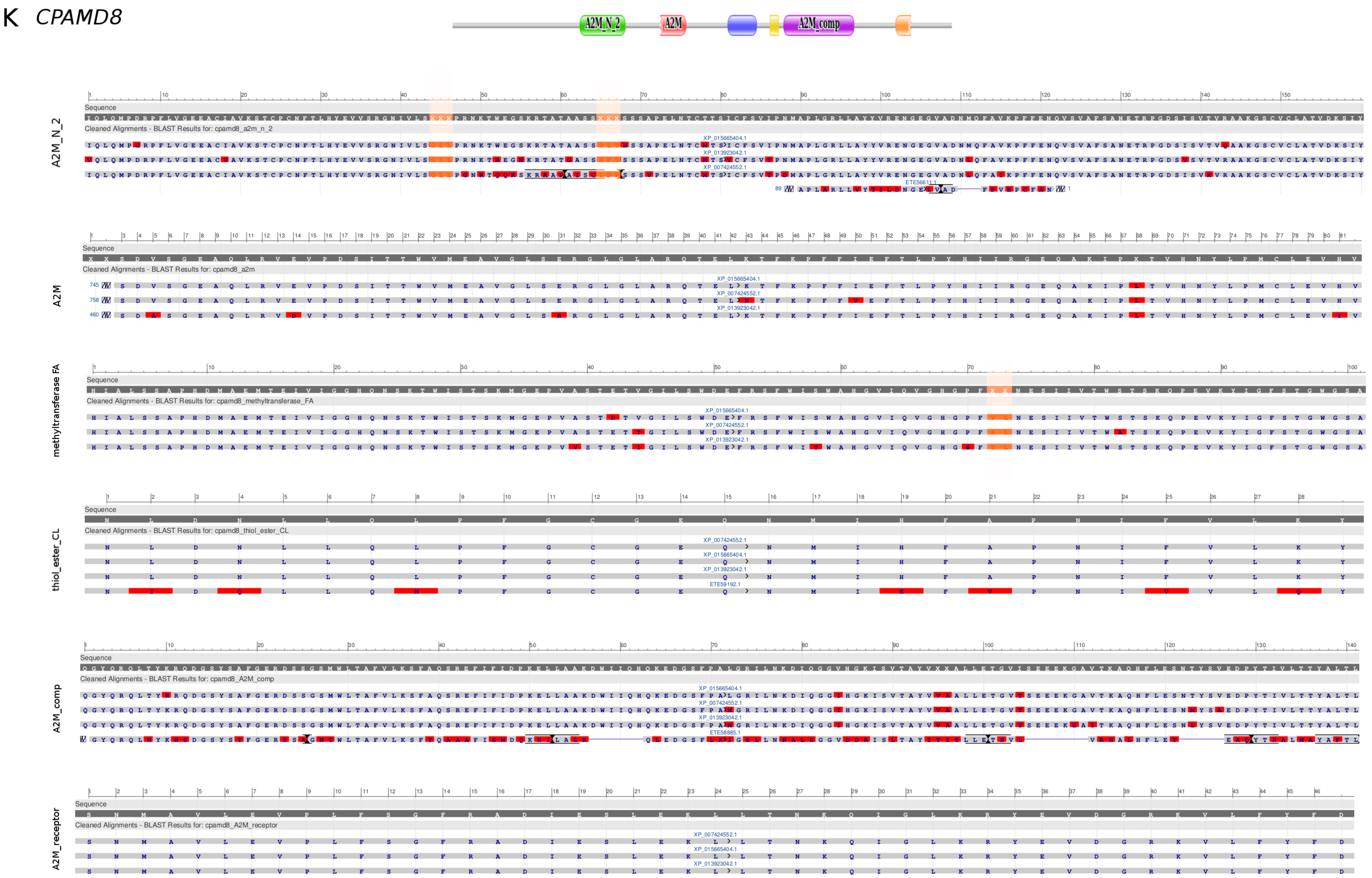

K CPAMD8
A2M_N_2
A2M
A2M_comp
Sequence
Cleaned Alignments - BLAST Results for: cpamd8_a2m_n_2
Cleaned Alignments - BLAST Results for: cpamd8_a2m
Cleaned Alignments - BLAST Results for: cpamd8_methyltransferase_FA
Cleaned Alignments - BLAST Results for: cpamd8_thiol_ester_CL
Cleaned Alignments - BLAST Results for: cpamd8_A2M_comp
Cleaned Alignments - BLAST Results for: cpamd8_A2M_receptor
XP_015665404.1
XP_013923042.1
XP_007424552.1
ETE56611.1
ETE59192.1
ETE58885.1
methyltransferase FA
thiol_ester_CL
A2M_receptor

L *CRISP*

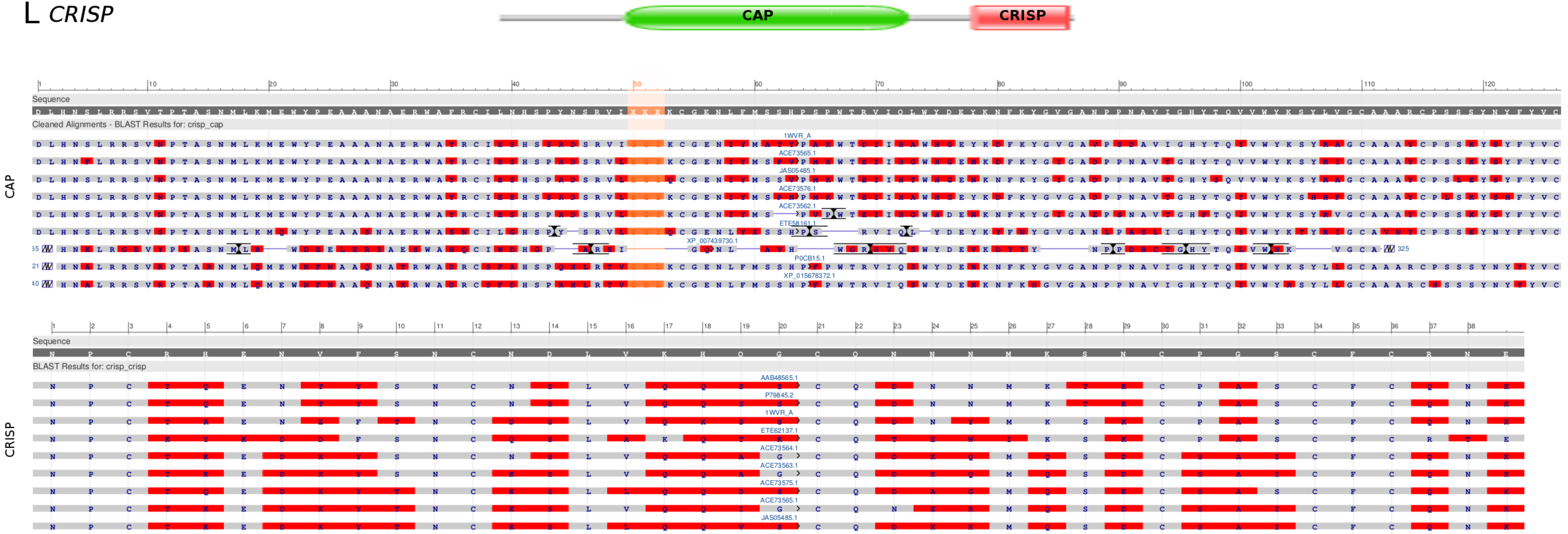
CAP
CRISP
Sequence
Cleaned Alignments - BLAST Results for: crisp_cap
1WVR_A
ACE73565.1
JAS05485.1
ACE73576.1
ACE73562.1
ETE58161.1
XP_007439730.1
P0CB15.1
XP_015678372.1
CAP
Sequence
BLAST Results for: crisp_crisp
AAB48565.1
P79845.2
1WVR_A
ETE62137.1
ACE73564.1
ACE73563.1
ACE73575.1
ACE73565.1
JAS05485.1
CRISP

M

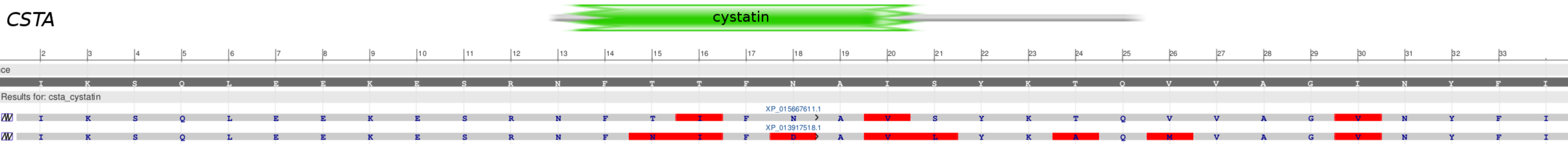
CSTA
cystatin
Sequence
X I K S Q L E E K E S R N F T T F N A I S Y K T Q V V A G I N Y F I
BLAST Results for: csta_cystatin
XP_015667611.1
XP_013917518.1

N *EDN1*

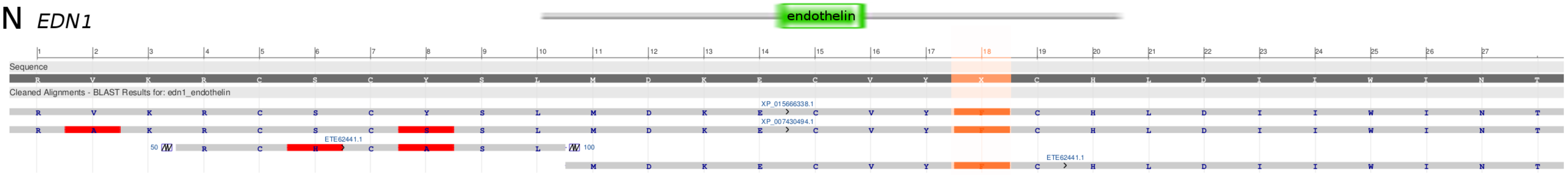
endothelin
Sequence
R V K R C S C Y S L M D K E C V Y X C H L D I I W I N T
Cleaned Alignments - BLAST Results for: edn1_endothelin
XP_015666338.1
XP_007430494.1
ETE62441.1
50
100
ETE62441.1

O *EDN3*

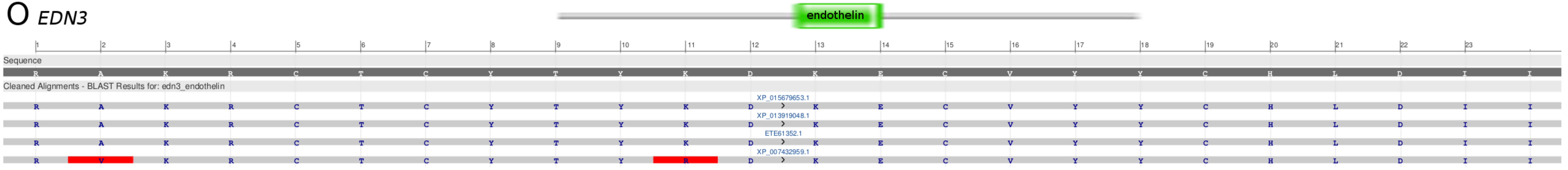

endothelin
1 2 3 4 5 6 7 8 9 10 11 12 13 14 15 16 17 18 19 20 21 22 23
Sequence
R A K R C T C Y T Y K D K E C V Y Y C H L D I I
Cleaned Alignments - BLAST Results for: edn3_endothelin
XP_015679653.1
R A K R C T C Y T Y K D K E C V Y Y C H L D I I
XP_013919048.1
R A K R C T C Y T Y K D K E C V Y Y C H L D I I
ETE61352.1
R A K R C T C Y T Y K D K E C V Y Y C H L D I I
XP_007432959.1
R V K R C T C Y T Y R D K E C V Y Y C H L D I I

P *F5*

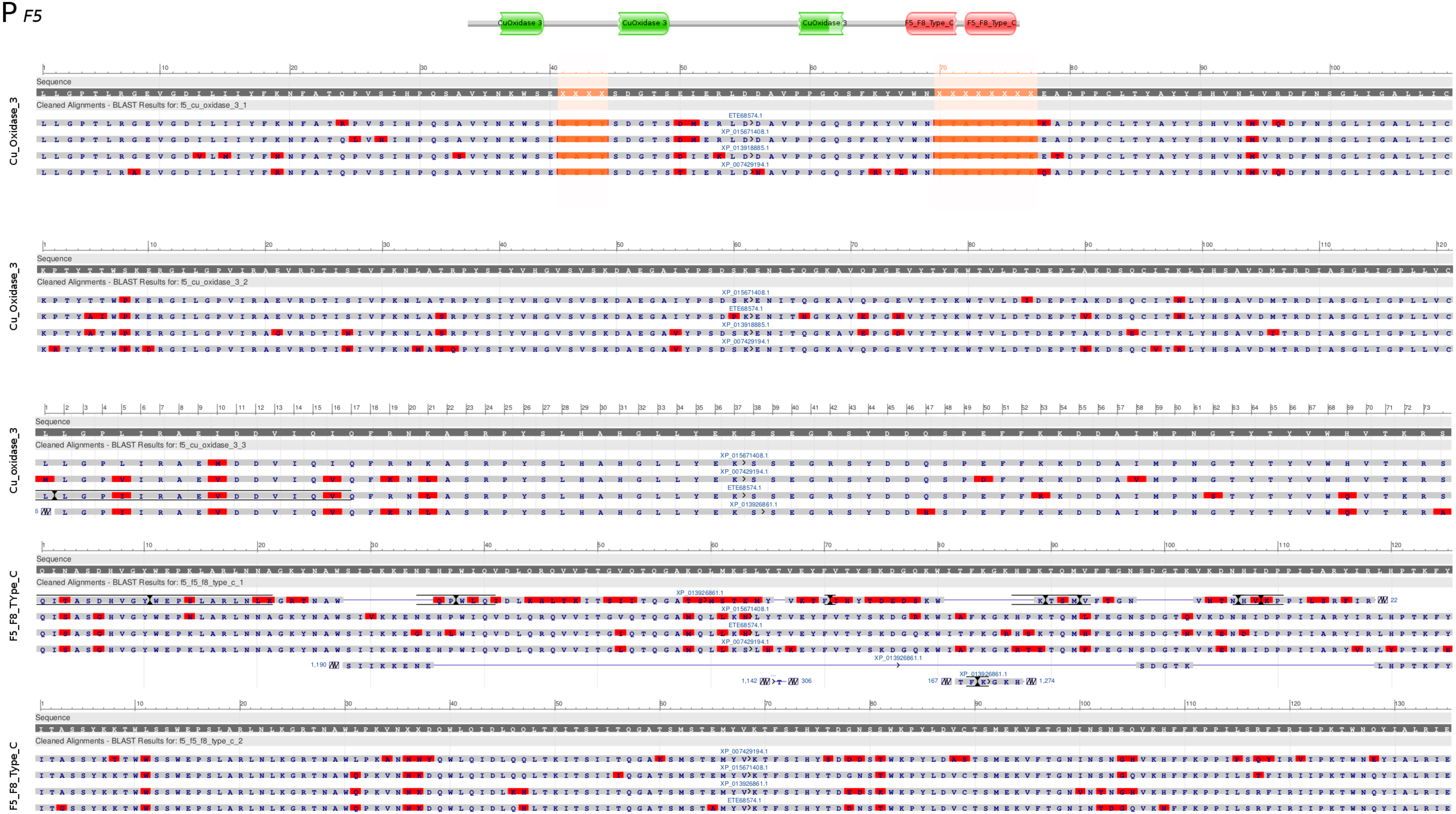
CuOxidase 3
CuOxidase 3
CuOxidase 3
F5_F8_Type_C
F5_F8_Type_C
Cu_Oxidase_3
Sequence
Cleaned Alignments - BLAST Results for: f5_cu_oxidase_3_1
ETE68574.1
XP_015671408.1
XP_013918885.1
XP_007429194.1
Cu_Oxidase_3
Sequence
Cleaned Alignments - BLAST Results for: f5_cu_oxidase_3_2
XP_015671408.1
ETE68574.1
XP_013918885.1
XP_007429194.1
Cu_oxidase_3
Sequence
Cleaned Alignments - BLAST Results for: f5_cu_oxidase_3_3
XP_015671408.1
XP_007429194.1
ETE68574.1
XP_013926861.1
F5_F8_TYpe_C
Sequence
Cleaned Alignments - BLAST Results for: f5_f5_f8_type_c_1
XP_013926861.1
XP_015671408.1
ETE68574.1
XP_007429194.1
XP_013926861.1
XP_013926861.1
F5_F8_Type_C
Sequence
Cleaned Alignments - BLAST Results for: f5_f5_f8_type_c_2
XP_007429194.1
XP_015671408.1
XP_013926861.1
ETE68574.1

Q *KLK14*

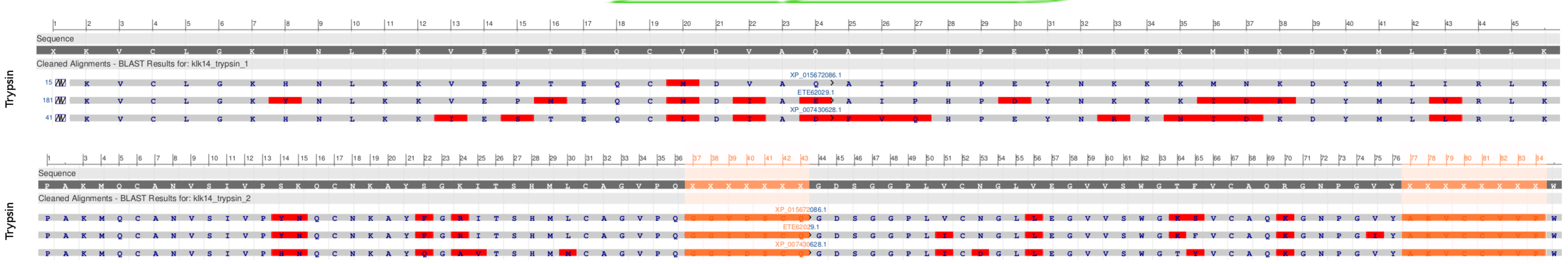
Trypsin
Trypsin
Sequence
Cleaned Alignments - BLAST Results for: klk14_trypsin_1
XP_015672086.1
ETE62029.1
XP_007430628.1
Trypsin
Sequence
Cleaned Alignments - BLAST Results for: klk14_trypsin_2
Trypsin

R *KLKB1*

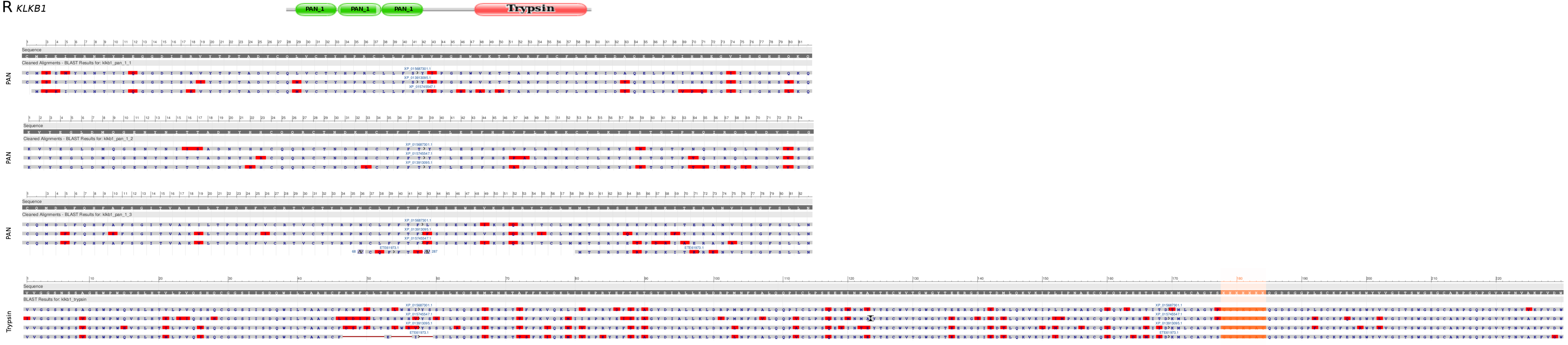
PAN_1
PAN_1
PAN_1
Trypsin
PAN
Sequence
Cleaned Alignments - BLAST Results for: klkb1_pan_1_1
PAN
Sequence
Cleaned Alignments - BLAST Results for: klkb1_pan_1_2
PAN
Sequence
Cleaned Alignments - BLAST Results for: klkb1_pan_1_3
Trypsin
Sequence
BLAST Results for: klkb1_trypsin

S *kunitoxin*

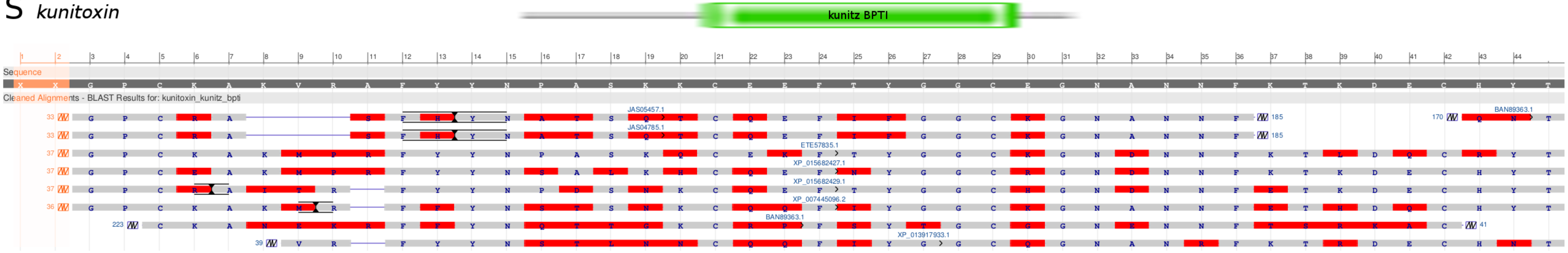

kunitz BPTI
Sequence
Cleaned Alignments - BLAST Results for: kunitoxin_kunitz_bpti
JAS05457.1
BAN89363.1
JAS04785.1
ETE57835.1
XP_015682427.1
XP_015682429.1
XP_007445096.2
BAN89363.1
XP_013917933.1

T *NGF*

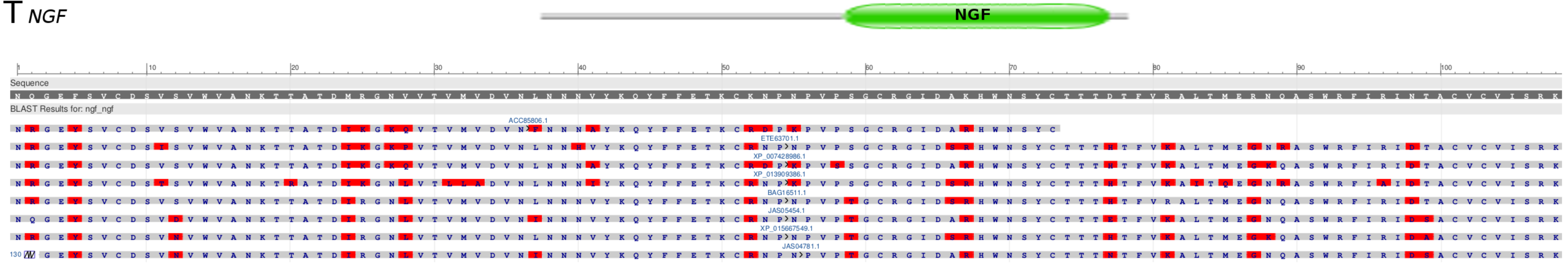
NGF
Sequence
BLAST Results for: ngf_ngf
ACC85806.1
ETE63701.1
XP_007428986.1
XP_013909386.1
BAG16511.1
JAS05454.1
XP_015667549.1
JAS04781.1

U *NPR1*

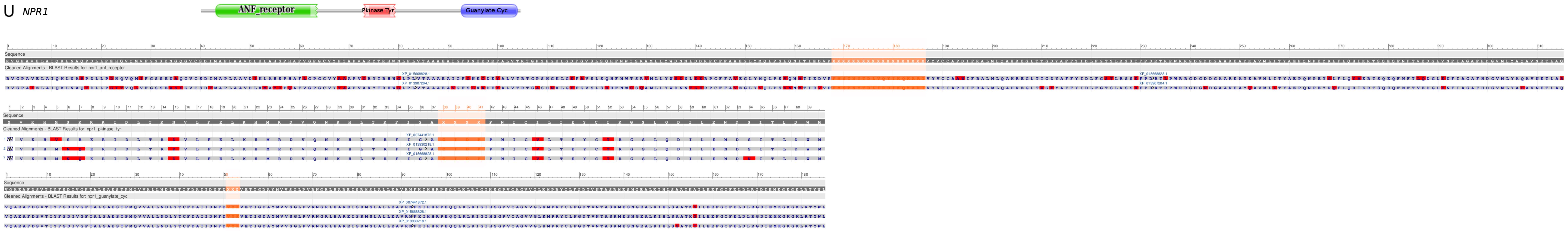
ANF_receptor
Pkinase Tyr
Guanylate Cyc
Sequence
Cleaned Alignments - BLAST Results for: npr1_anf_receptor
XP_015668828.1
XP_013907204.1
Sequence
Cleaned Alignments - BLAST Results for: npr1_pkinase_tyr
XP_007441872.1
XP_013930218.1
XP_015668828.1
Sequence
Cleaned Alignments - BLAST Results for: npr1_guanylate_cyc
XP_007441872.1
XP_015668828.1
XP_013930218.1

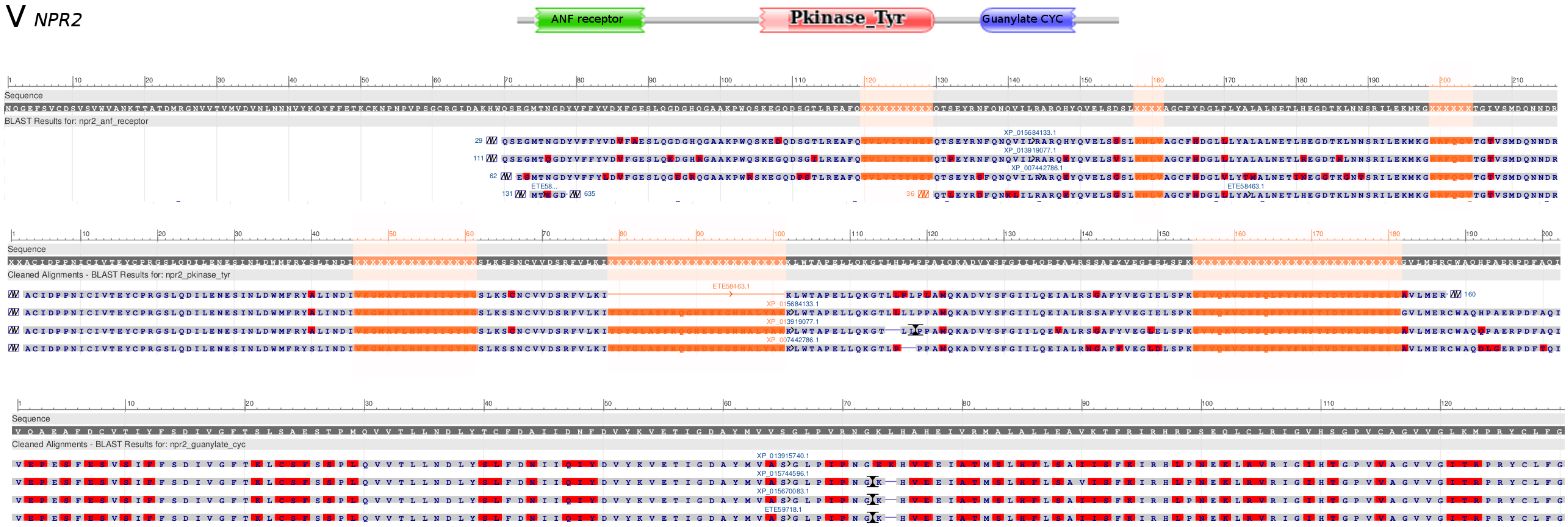
V NPR2
ANF receptor
Pkinase_Tyr
Guanylate CYC
Sequence
BLAST Results for: npr2_anf_receptor
XP_015684133.1
XP_013919077.1
XP_007442786.1
ETE58463.1
Sequence
Cleaned Alignments - BLAST Results for: npr2_pkinase_tyr
ETE58463.1
XP_015684133.1
XP_013919077.1
XP_007442786.1
Sequence
Cleaned Alignments - BLAST Results for: npr2_guanylate_cyc
XP_013915740.1
XP_015744596.1
XP_015670083.1
ETE59718.1

W *NPR3*

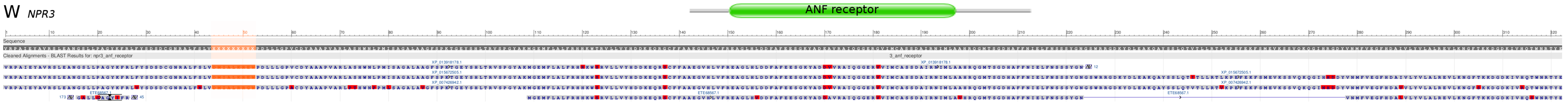
ANF receptor
Sequence
Cleaned Alignments - BLAST Results for: npr3_anf_receptor

X *PLA2G1B*

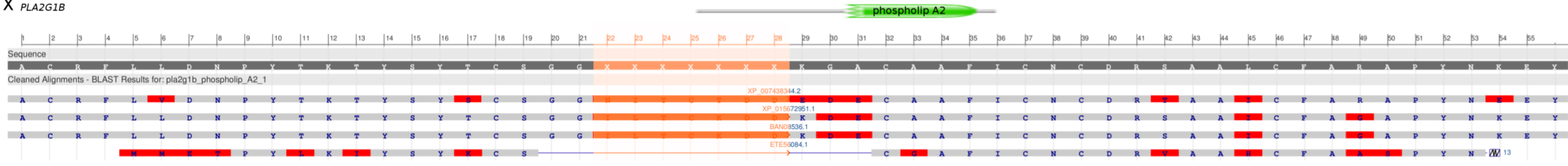
phospholip A2
Sequence
Cleaned Alignments - BLAST Results for: pla2g1b_phospholip_A2_1
XP_007438344.2
XP_015672951.1
BAN08536.1
ETE56084.1

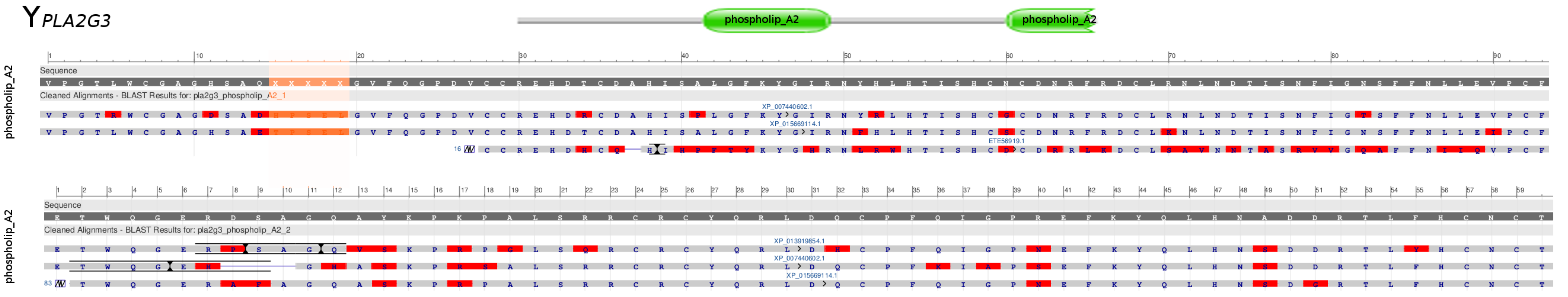

Y PLA2G3
phospholip_A2
phospholip_A2
phospholip_A2
Sequence
Cleaned Alignments - BLAST Results for: pla2g3_phospholip_A2_1
XP_007440602.1
XP_015669114.1
ETE56919.1
phospholip_A2
Sequence
Cleaned Alignments - BLAST Results for: pla2g3_phospholip_A2_2
XP_013919854.1
XP_007440602.1
XP_015669114.1

Z *PLA2G4A*

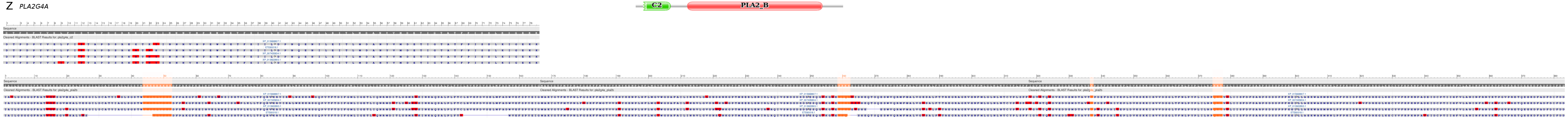
C2
PLA2_B
Sequence
Cleaned Alignments - BLAST Results for: pla2g4a_c2
XP_015668807.1
ETE60018.1
XP_007435834.1
XP_013922893.1

AA *PLA2G4C*

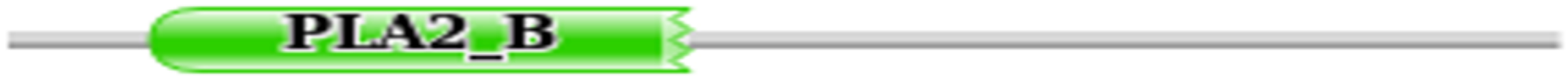
PLA2_B

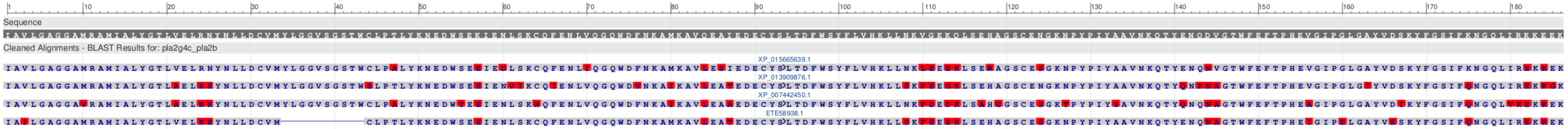
Sequence
Cleaned Alignments - BLAST Results for: pla2g4c_pla2b
XP_015665639.1
XP_013909876.1
XP_007442450.1
ETE58938.1

AB *PLA2G6*

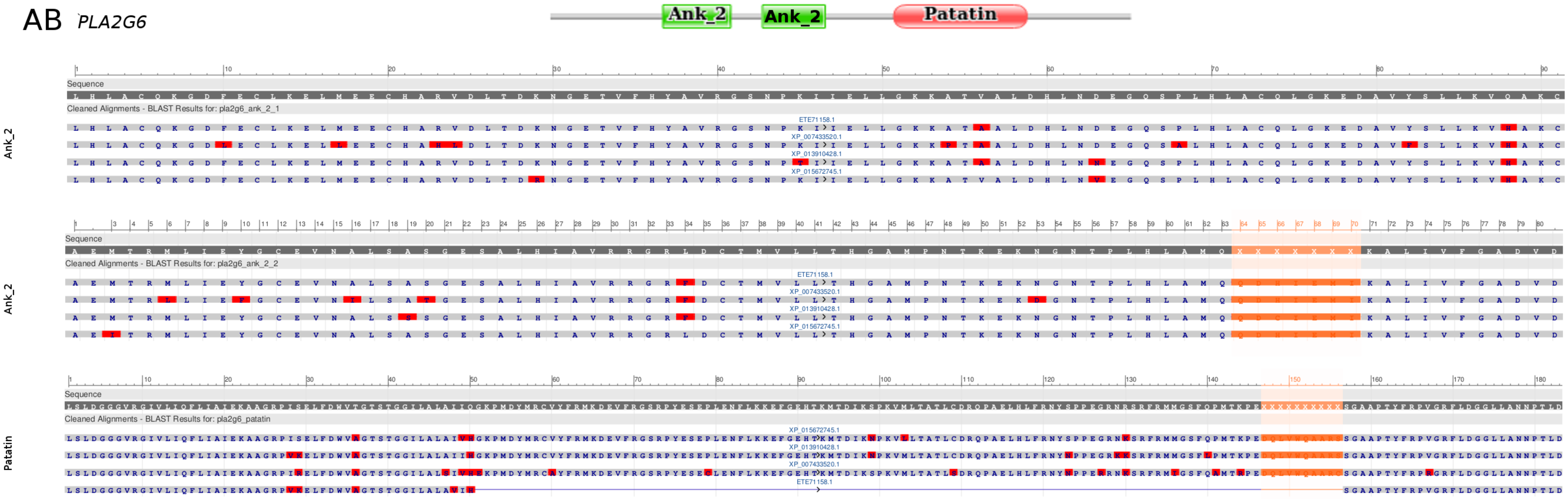
Ank_2
Ank_2
Patatin
Sequence
Cleaned Alignments - BLAST Results for: pla2g6_ank_2_1
ETE71158.1
XP_007433520.1
XP_013910428.1
XP_015672745.1
Ank_2
Sequence
Cleaned Alignments - BLAST Results for: pla2g6_ank_2_2
ETE71158.1
XP_007433520.1
XP_013910428.1
XP_015672745.1
Ank_2
Sequence
Cleaned Alignments - BLAST Results for: pla2g6_patatin
XP_015672745.1
XP_013910428.1
XP_007433520.1
ETE71158.1
Patatin

AC *PLA2G7*

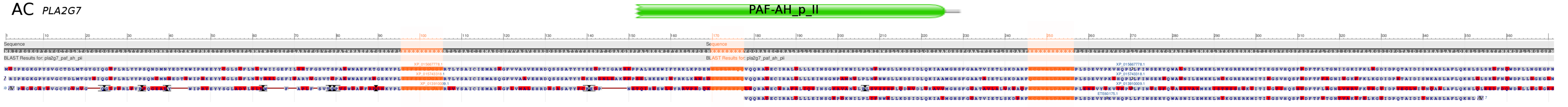

AD *PLA2G10*

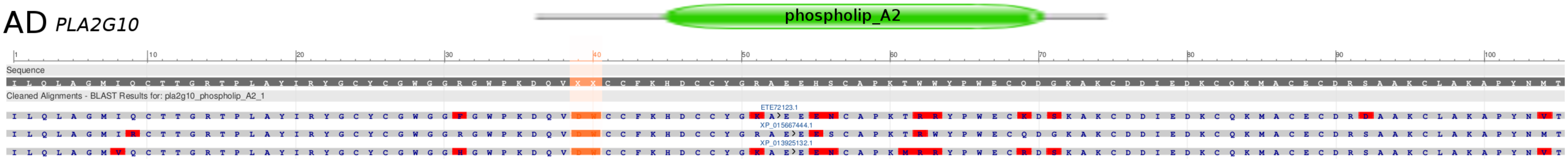
phospholip_A2
1
10
20
30
40
50
60
70
80
90
100
Sequence
ILQLAGMIQCTTGRTPLAYIRYGCYCGWGGRGWPKDQVXXCCFKHDCCYGRAEEHSCAPKTWWYPWECQDGKAKCDDIEDKCQKMACECDRSAAKCLAKAPYNMT
Cleaned Alignments - BLAST Results for: pla2g10_phospholip_A2_1
ETE72123.1
ILQLAGMIQCTTGRTPLAYIRYGCYCGWGGFGWPKDQVDWCCFKHDCCYGKA>EEENCAPKTRRYPWECKDSKAKCDDIEDKCQKMACECDRDAAKCLAKAPYNVT
XP_015667444.1
ILQLAGMIRCTTGRTPLAYIRYGCYCGWGGRGWPKDQVDWCCFKHDCCYGRAE>EESCAPKTRWYPWECQDGKAKCDDIEDKCQKMACECDRSAAKCLAKAPYNMT
XP_013925132.1
ILQLAGMVQCTTGRTPLAYIRYGCYCGWGGHGWPKDQVDWCCFKHDCCYGKAE>EENCAPKMRRYPWECRDSKAKCDDIEDKCQKMACECDRSAAKCLAKAPYNVT

AE *PLA2G12A*

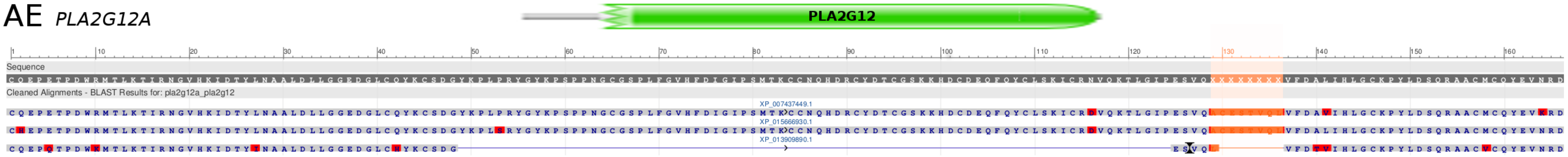
PLA2G12
Sequence
Cleaned Alignments - BLAST Results for: pla2g12a_pla2g12
XP_007437449.1
XP_015666930.1
XP_013909890.1

AF *PLA2G12B*

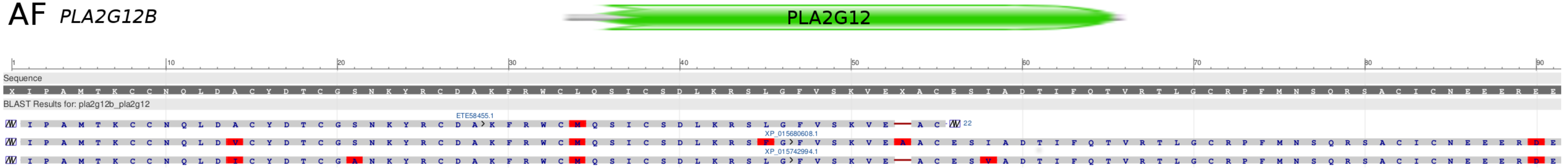
PLA2G12
1
10
20
30
40
50
60
70
80
90
Sequence
X I P A M T K C C N Q L D A C Y D T C G S N K Y R C D A K F R W C L Q S I C S D L K R S L G F V S K V E X A C E S I A D T I F Q T V R T L G C R P F M N S Q R S A C I C N E E E R E E
BLAST Results for: pla2g12b_pla2g12
ETE58455.1
22
XP_015680608.1
XP_015742994.1

AG *PLA2G15*

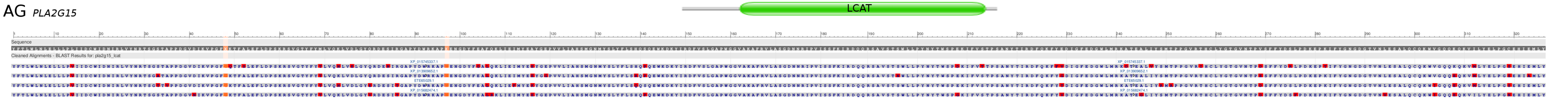
LCAT
Sequence
Cleaned Alignments - BLAST Results for: pla2g15_lcat
XP_015745337.1
XP_013909652.1
ETE65029.1
XP_015682474.1

AH *PLA2R1*

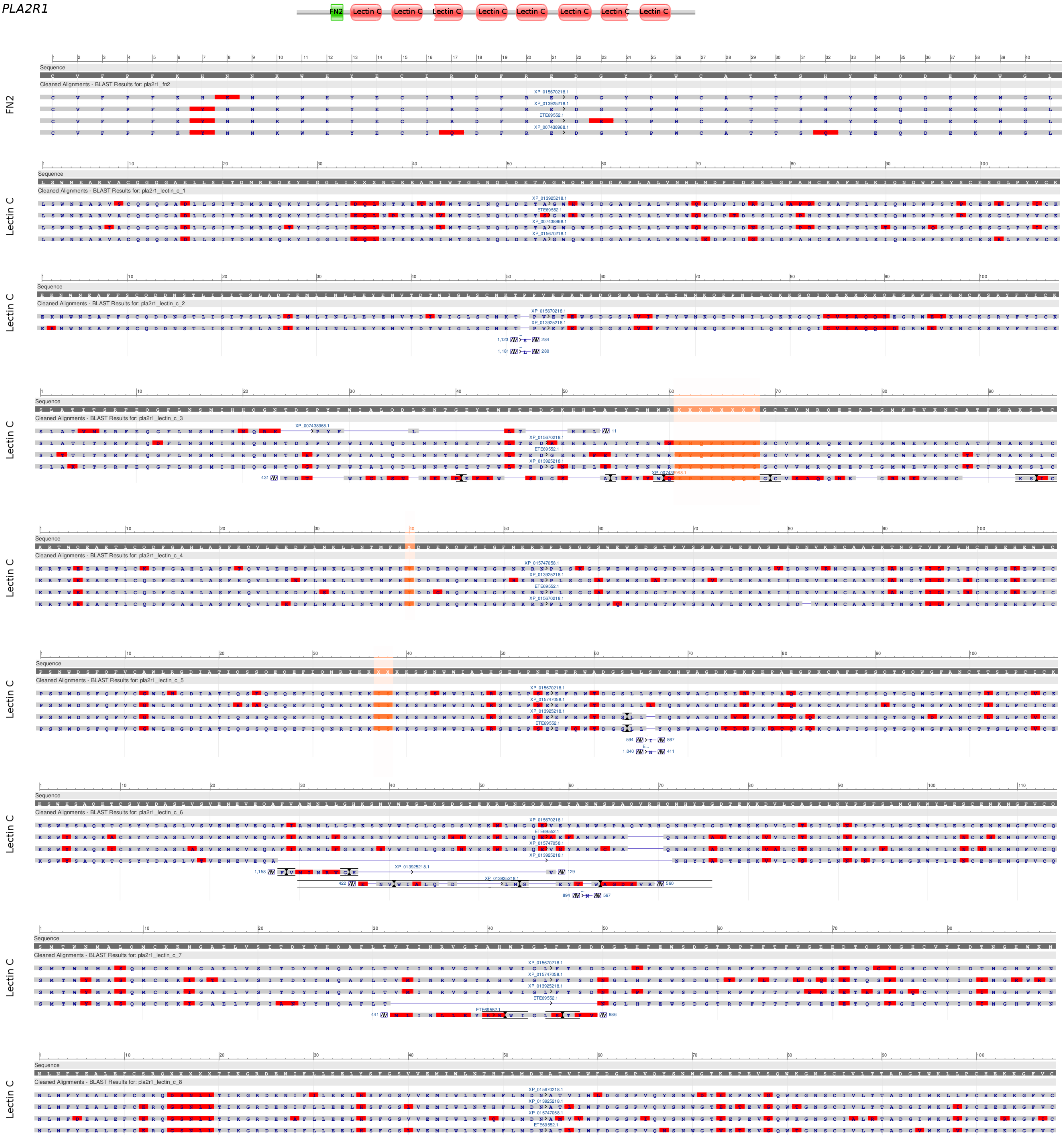
FN2
Lectin C
Sequence
Cleaned Alignments - BLAST Results for: pla2r1_fn2
Cleaned Alignments - BLAST Results for: pla2r1_lectin_c_1
Cleaned Alignments - BLAST Results for: pla2r1_lectin_c_2
Cleaned Alignments - BLAST Results for: pla2r1_lectin_c_3
Cleaned Alignments - BLAST Results for: pla2r1_lectin_c_4
Cleaned Alignments - BLAST Results for: pla2r1_lectin_c_5
Cleaned Alignments - BLAST Results for: pla2r1_lectin_c_6
Cleaned Alignments - BLAST Results for: pla2r1_lectin_c_7
Cleaned Alignments - BLAST Results for: pla2r1_lectin_c_8
XP_015670218.1
XP_013925218.1
ETE69552.1
XP_007438968.1
XP_015747058.1

AI *PLAA*

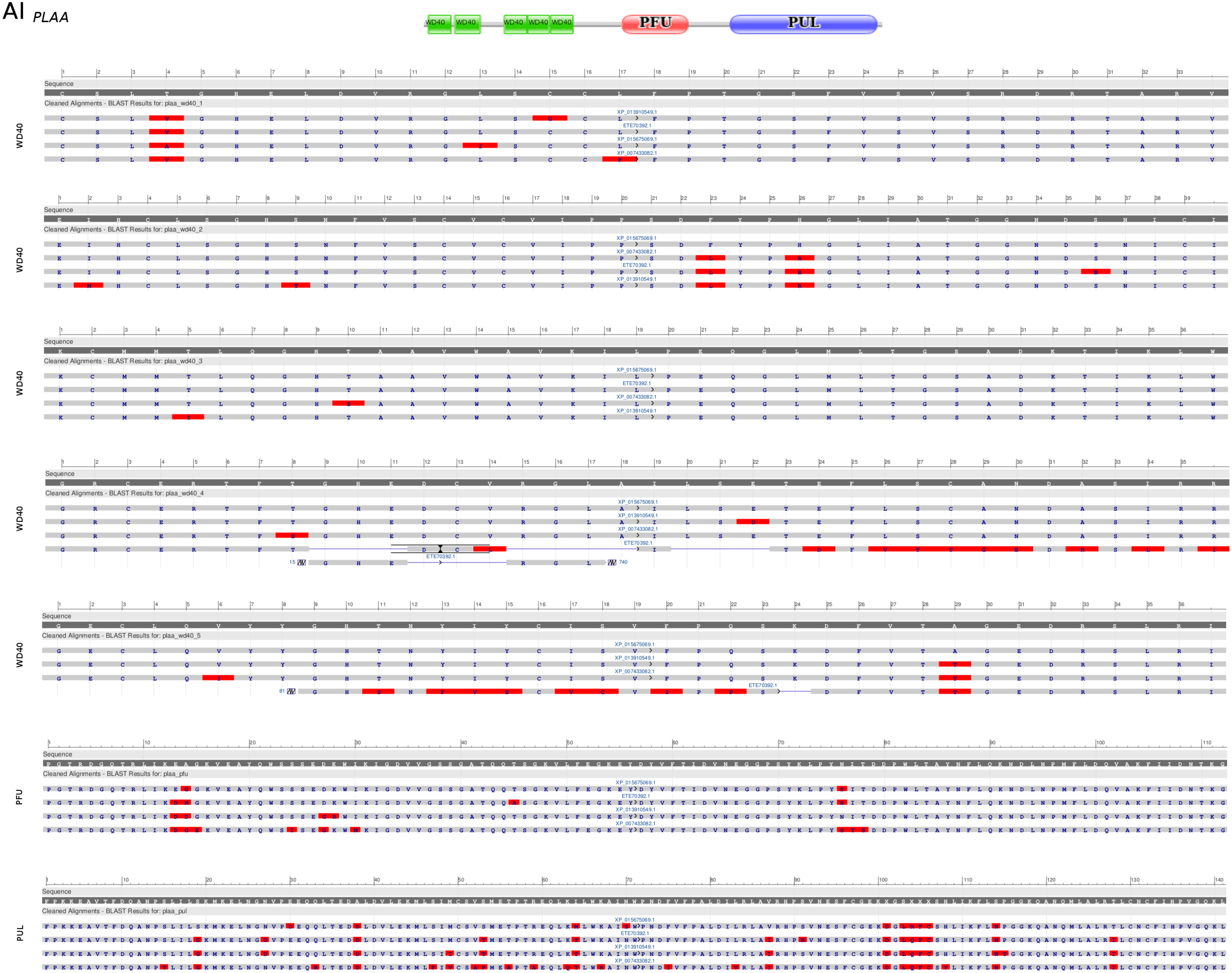

WD40
WD40
WD40
WD40
WD40
PFU
PUL
Sequence
Cleaned Alignments - BLAST Results for: plaa_wd40_1
XP_013910549.1
ETE70392.1
XP_015675069.1
XP_007433082.1
WD40
Sequence
Cleaned Alignments - BLAST Results for: plaa_wd40_2
XP_015675069.1
XP_007433082.1
ETE70392.1
XP_013910549.1
WD40
Sequence
Cleaned Alignments - BLAST Results for: plaa_wd40_3
XP_015675069.1
ETE70392.1
XP_007433082.1
XP_013910549.1
WD40
Sequence
Cleaned Alignments - BLAST Results for: plaa_wd40_4
XP_015675069.1
XP_013910549.1
XP_007433082.1
ETE70392.1
WD40
Sequence
Cleaned Alignments - BLAST Results for: plaa_wd40_5
XP_015675069.1
XP_013910549.1
XP_007433082.1
ETE70392.1
WD40
Sequence
Cleaned Alignments - BLAST Results for: plaa_pfu
XP_015675069.1
ETE70392.1
XP_013910549.1
XP_007433082.1
PFU
Sequence
Cleaned Alignments - BLAST Results for: plaa_pul
XP_015675069.1
ETE70392.1
XP_013910549.1
XP_007433082.1
PUL

AJ *PROK1*

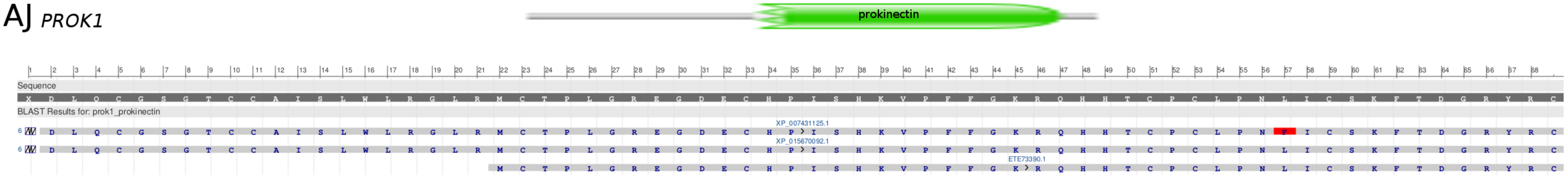
prokinectin
Sequence
X D L Q C G S G T C C A I S L W L R G L R M C T P L G R E G D E C H P I S H K V P F F G K R Q H H T C P C L P N L I C S K F T D G R Y R C
BLAST Results for: prok1_prokinectin
XP_007431125.1
XP_015670092.1
ETE73390.1

AK *PROK2*

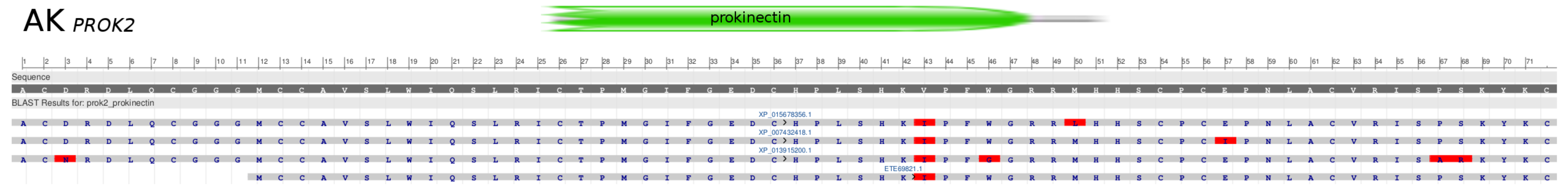

prokinectin
Sequence
A C D R D L Q C G G G M C C A V S L W I Q S L R I C T P M G I F G E D C H P L S H K V P F W G R R M H H S C P C E P N L A C V R I S P S K Y K C
BLAST Results for: prok2_prokinectin
XP_015678356.1
XP_007432418.1
XP_013915200.1
ETE69821.1

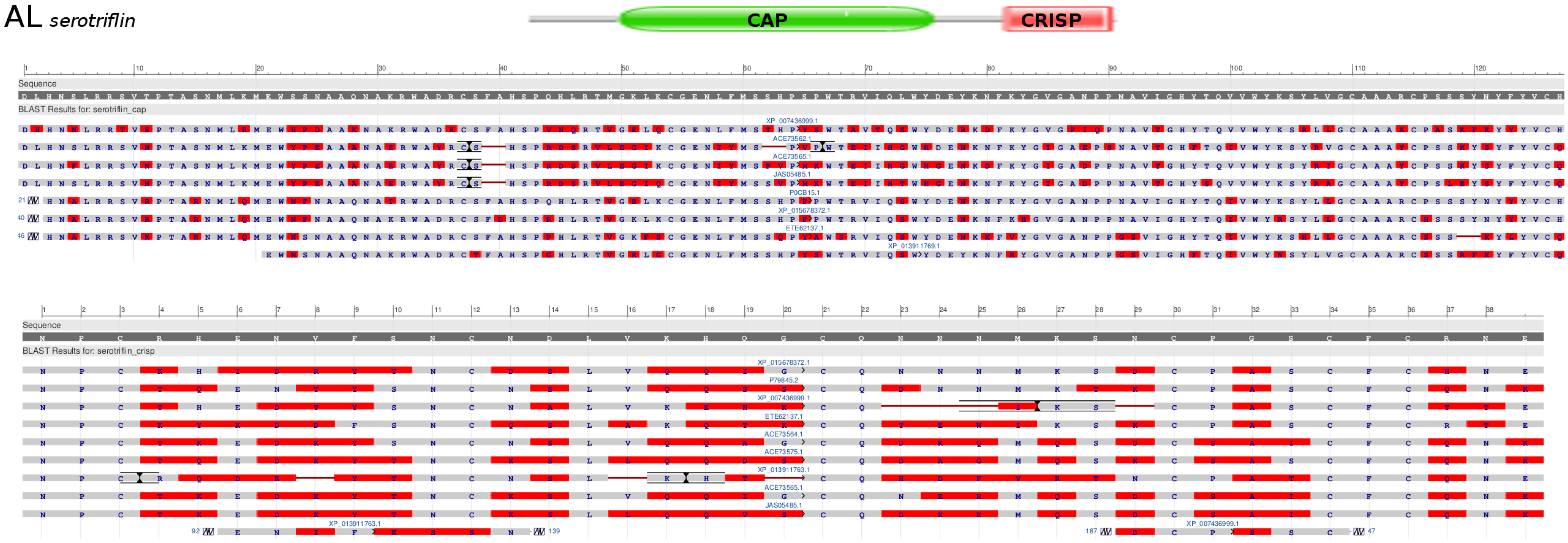
AL serotriflin
CAP
CRISP
Sequence
BLAST Results for: serotriflin_cap
XP_007436999.1
ACE73562.1
ACE73565.1
JAS05485.1
P0CB15.1
XP_015678372.1
ETE62137.1
XP_013911769.1
Sequence
BLAST Results for: serotriflin_crisp
XP_015678372.1
P79845.2
XP_007436999.1
ETE62137.1
ACE73564.1
ACE73575.1
XP_013911763.1
ACE73565.1
JAS05485.1
XP_013911763.1
XP_007436999.1

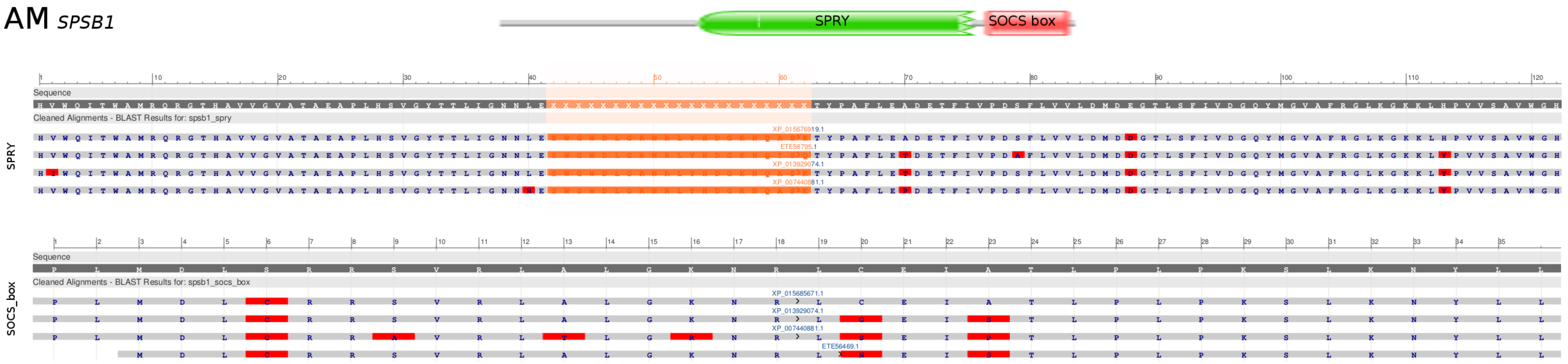
AM SPSB1
SPRY
SOCS box
Sequence
Cleaned Alignments - BLAST Results for: spsb1_spry
XP_015676919.1
ETE58795.1
XP_013929074.1
XP_007440881.1
Sequence
Cleaned Alignments - BLAST Results for: spsb1_socs_box
XP_015685671.1
XP_013929074.1
XP_007440881.1
ETE56469.1
SPRY
SOCS_box

AN *SPSB3*

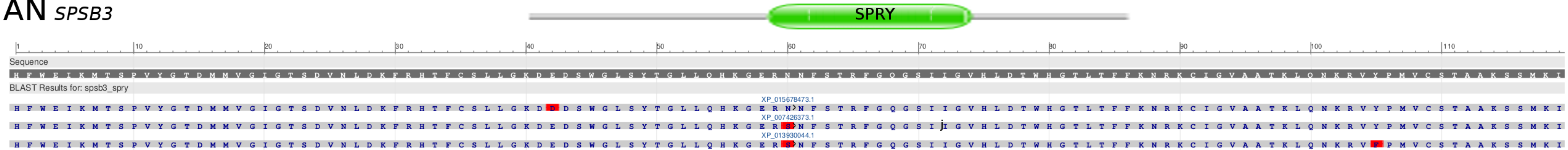
SPRY
1
10
20
30
40
50
60
70
80
90
100
110
Sequence
HFWEIKMTSPVYGTDMMVGIGTSDVNLDKFRHTFCSLLGKDEDSWGLSYTGLLQHKGERNNFSTRFGQGSIIGVHLDTWHGTLTFFKNRKCIGVAATKLQNKRVYPMVCSTAAKSSMKI
BLAST Results for: spsb3_spry
XP_015678473.1
HFWEIKMTSPVYGTDMMVGIGTSDVNLDKFRHTFCSLLGKDDDSWGLSYTGLLQHKGERNNFSTRFGQGSIIGVHLDTWHGTLTFFKNRKCIGVAATKLQNKRVYPMVCSTAAKSSMKI
XP_007426373.1
HFWEIKMTSPVYGTDMMVGIGTSDVNLDKFRHTFCSLLGKDEDSWGLSYTGLLQHKGERSNFSTRFGQGSIIGVHLDTWHGTLTFFKNRKCIGVAATKLQNKRVYPMVCSTAAKSSMKI
XP_013930044.1
HFWEIKMTSPVYGTDMMVGIGTSDVNLDKFRHTFCSLLGKDEDSWGLSYTGLLQHKGERSNFSTRFGQGSIIGVHLDTWHGTLTFFKNRKCIGVAATKLQNKRVFPMVCSTAAKSSMKI

AO *SPSB4*

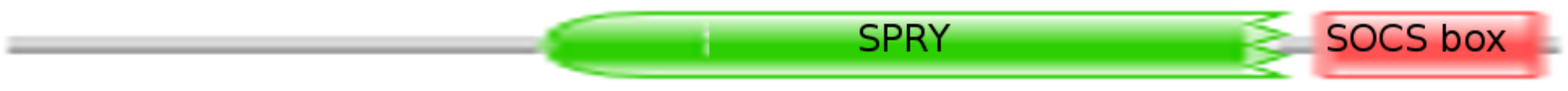
SPRY
SOCS box

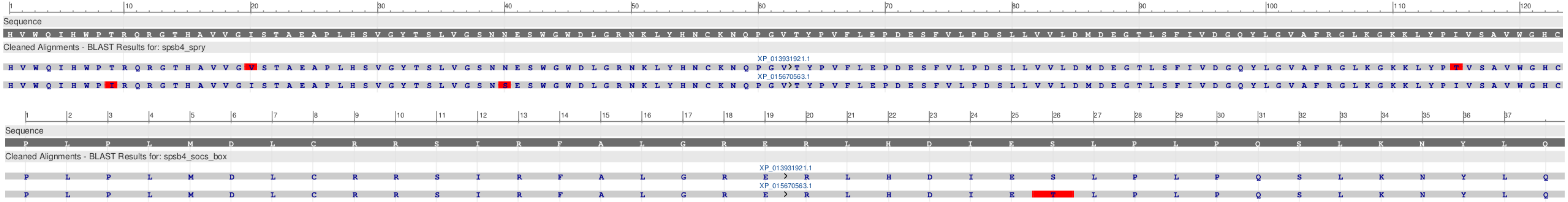
1 10 20 30 40 50 60 70 80 90 100 110 120
Sequence
H V W Q I H W P T R Q R G T H A V V G I S T A E A P L H S V G Y T S L V G S N N E S W G W D L G R N K L Y H N C K N Q P G V T Y P V F L E P D E S F V L P D S L L V V L D M D E G T L S F I V D G Q Y L G V A F R G L K G K K L Y P I V S A V W G H C
Cleaned Alignments - BLAST Results for: spsb4_spry
XP_013931921.1
H V W Q I H W P T R Q R G T H A V V G V S T A E A P L H S V G Y T S L V G S N N E S W G W D L G R N K L Y H N C K N Q P G V T Y P V F L E P D E S F V L P D S L L V V L D M D E G T L S F I V D G Q Y L G V A F R G L K G K K L Y P T V S A V W G H C
XP_015670563.1
H V W Q I H W P Y R Q R G T H A V V G I S T A E A P L H S V G Y T S L V G S N S E S W G W D L G R N K L Y H N C K N Q P G V T Y P V F L E P D E S F V L P D S L L V V L D M D E G T L S F I V D G Q Y L G V A F R G L K G K K L Y P I V S A V W G H C
1 2 3 4 5 6 7 8 9 10 11 12 13 14 15 16 17 18 19 20 21 22 23 24 25 26 27 28 29 30 31 32 33 34 35 36 37
Sequence
P L P L M D L C R R S I R F A L G R E R L H D I E S L P L P Q S L K N Y L Q
Cleaned Alignments - BLAST Results for: spsb4_socs_box
XP_013931921.1
P L P L M D L C R R S I R F A L G R E R L H D I E S L P L P Q S L K N Y L Q
XP_015670563.1
P L P L M D L C R R S I R F A L G R E R L H D I E T L P L P Q S L K N Y L Q

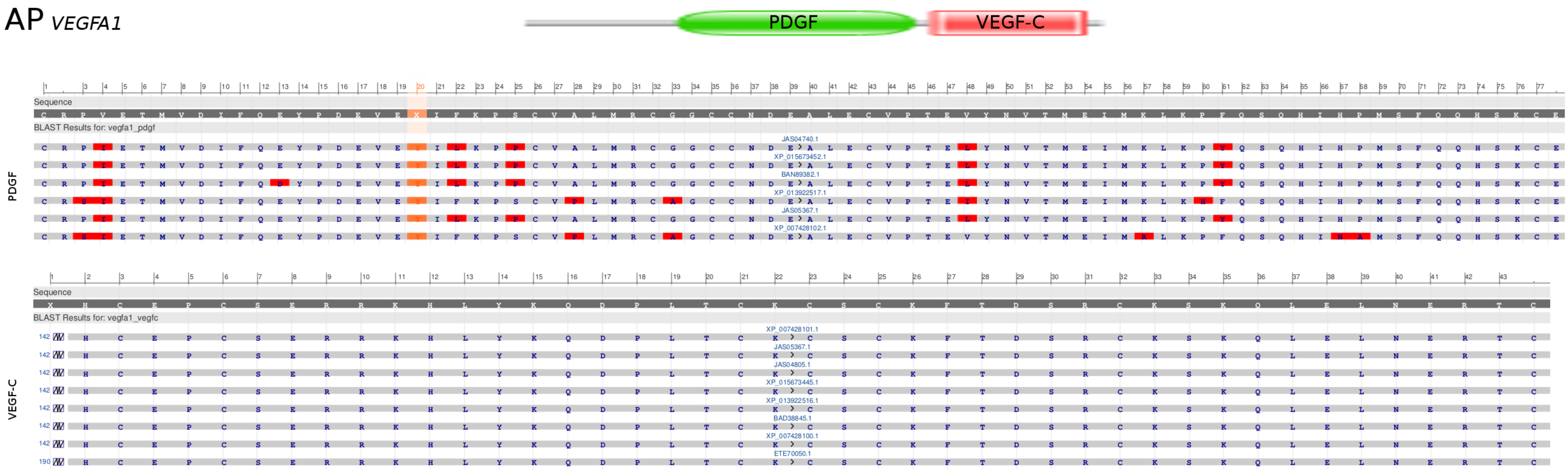
AP VEGFA1
PDGF
VEGF-C
Sequence
BLAST Results for: vegfa1_pdgf
JAS04740.1
XP_015673452.1
BAN89382.1
XP_013922517.1
JAS05367.1
XP_007428102.1
PDGF
Sequence
BLAST Results for: vegfa1_vegfc
XP_007428101.1
JAS05367.1
JAS04805.1
XP_015673445.1
XP_013922516.1
BAD38845.1
XP_007428100.1
ETE70050.1
VEGF-C

AQ *VEGFA2*

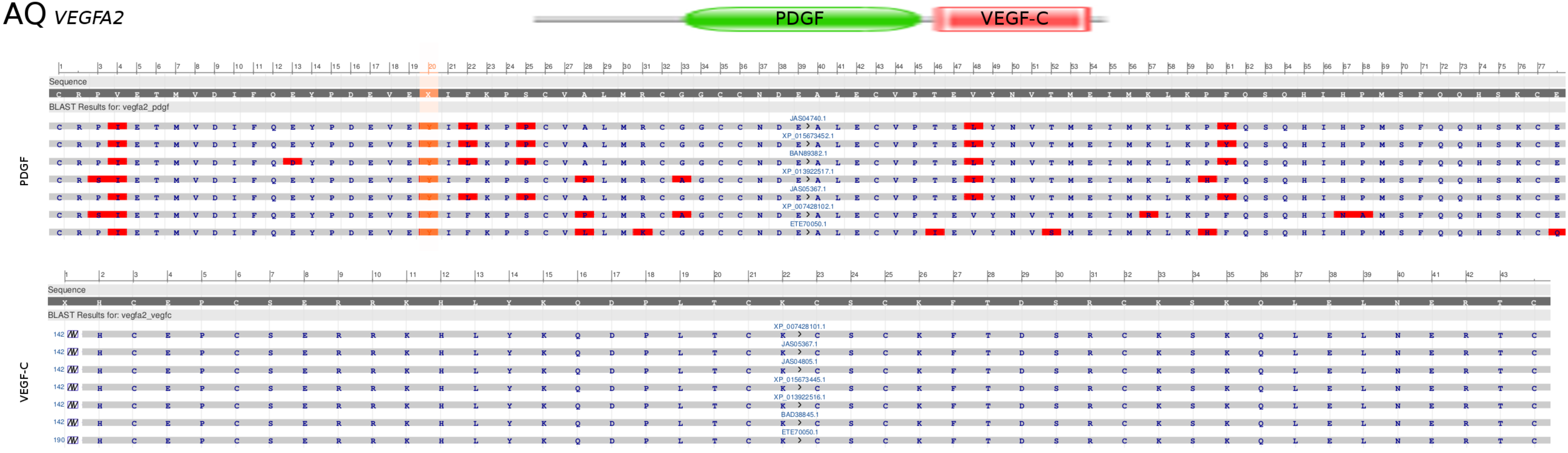
PDGF
VEGF-C
Sequence
CRPVETMVDIFQEYPDEVEXIFKPSCVALMRCGGCNDEALECVPTEVYNVTMEIMKLKPFQSQHIHPMSFQQHSKCE
BLAST Results for: vegfa2_pdgf
JAS04740.1
XP_015673452.1
BAN89382.1
XP_013922517.1
JAS05367.1
XP_007428102.1
ETE70050.1
PDGF
Sequence
XHCEPCSERRKHLYKQDPLTCKCSCKFTDSRCKSKQLELNERTC
BLAST Results for: vegfa2_vegfc
XP_007428101.1
JAS05367.1
JAS04805.1
XP_015673445.1
XP_013922516.1
BAD38845.1
ETE70050.1
VEGF-C

AR *VEGA3*

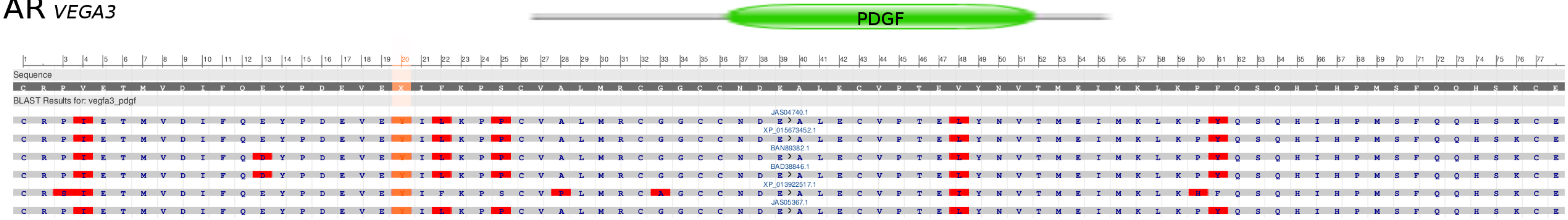
PDGF
Sequence
BLAST Results for: vegfa3_pdgf
JAS04740.1
XP_015673452.1
BAN89382.1
BAD38846.1
XP_013922517.1
JAS05367.1

AS *VEGFB*

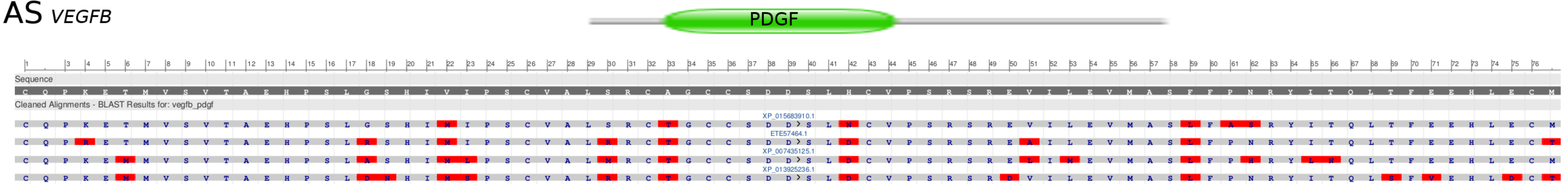
PDGF
Sequence
CQPKETMVSVTAEHPSLGSHIVIPSCVALSRCAGCCSDDSLHCVPSRSREVILEVMASFFPNRYITQLTFEEHLECM
Cleaned Alignments - BLAST Results for: vegfb_pdgf
XP_015683910.1
ETE57464.1
XP_007435125.1
XP_013925236.1

AT *VEGFC*

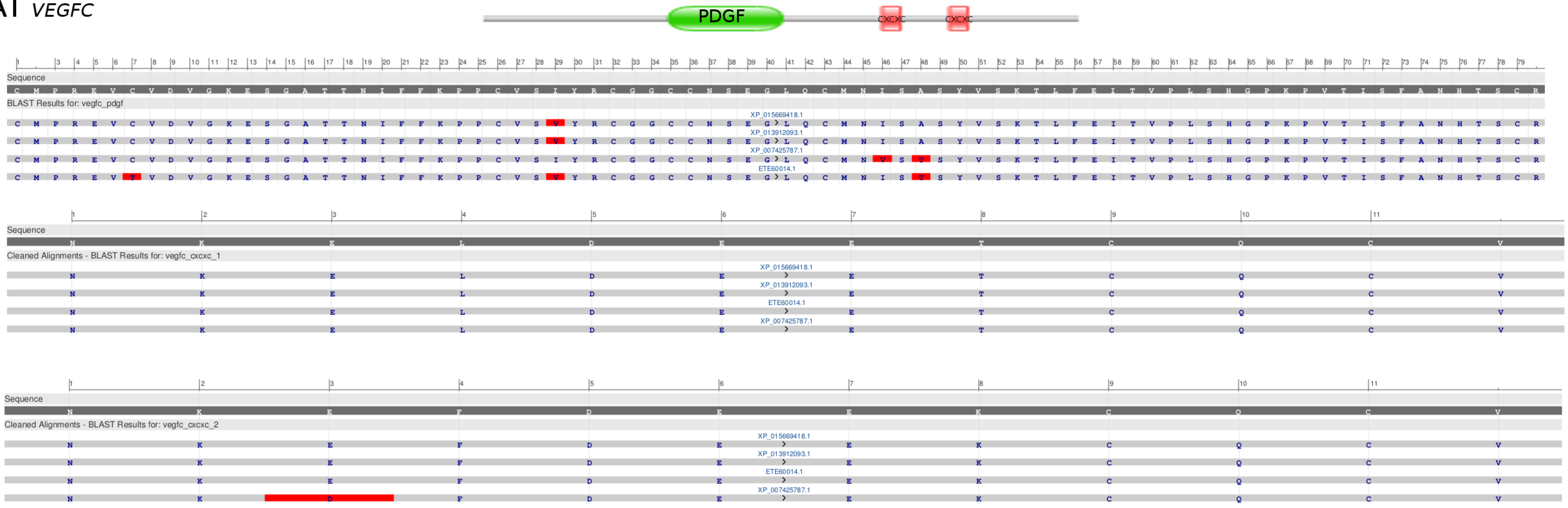
PDGF
CXCXC
CXCXC
Sequence
C M P R E V C V D V G K E S G A T T N I F F K P P C V S I Y R C G G C C N S E G L Q C M N I S A S Y V S K T L F E I T V P L S H G P K P V T I S F A N H T S C R
BLAST Results for: vegfc_pdgf
XP_015669418.1
XP_013912093.1
XP_007425787.1
ETE60014.1
Sequence
N K E L D E E T C Q C V
Cleaned Alignments - BLAST Results for: vegfc_cxcxc_1
XP_015669418.1
XP_013912093.1
ETE60014.1
XP_007425787.1
Sequence
N K E F D E E K C Q C V
Cleaned Alignments - BLAST Results for: vegfc_cxcxc_2
XP_015669418.1
XP_013912093.1
ETE60014.1
XP_007425787.1

AU *VEGFF*

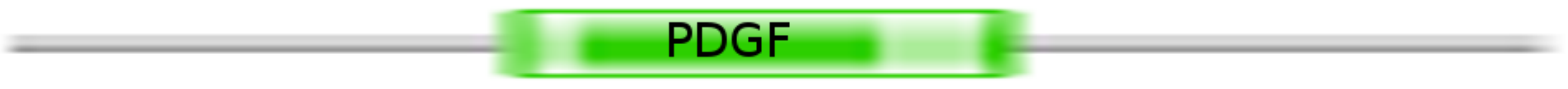
PDGF

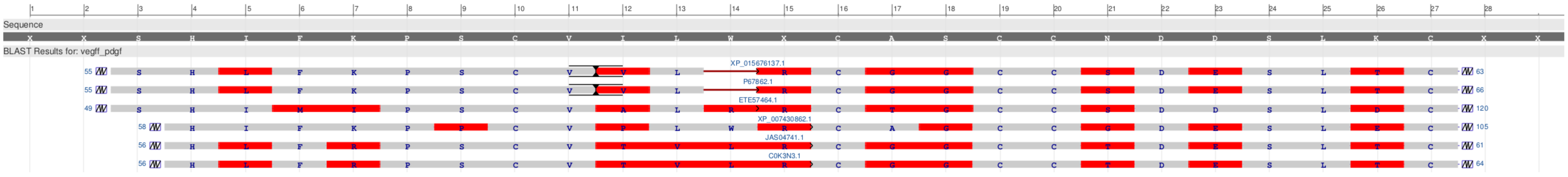
Sequence
BLAST Results for: vegff_pdgf
XP_015676137.1
P67862.1
ETE57464.1
XP_007430862.1
JAS04741.1
C0K3N3.1

AV *WAP*

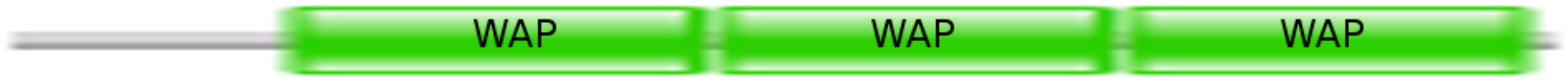
WAP
WAP
WAP

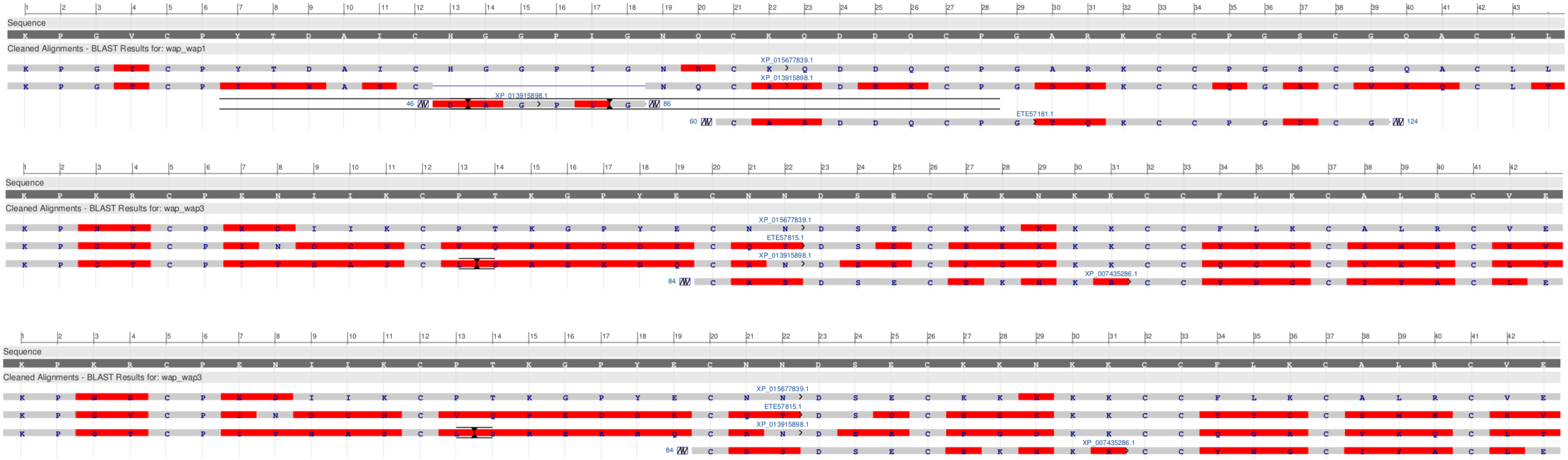
Sequence
Cleaned Alignments - BLAST Results for: wap_wap1
XP_015677839.1
XP_013915898.1
XP_013915898.1
46
86
ETE57181.1
60
124
Sequence
Cleaned Alignments - BLAST Results for: wap_wap3
XP_015677839.1
ETE57815.1
XP_013915898.1
XP_007435286.1
84
Sequence
Cleaned Alignments - BLAST Results for: wap_wap3
XP_015677839.1
ETE57815.1
XP_013915898.1
XP_007435286.1
84

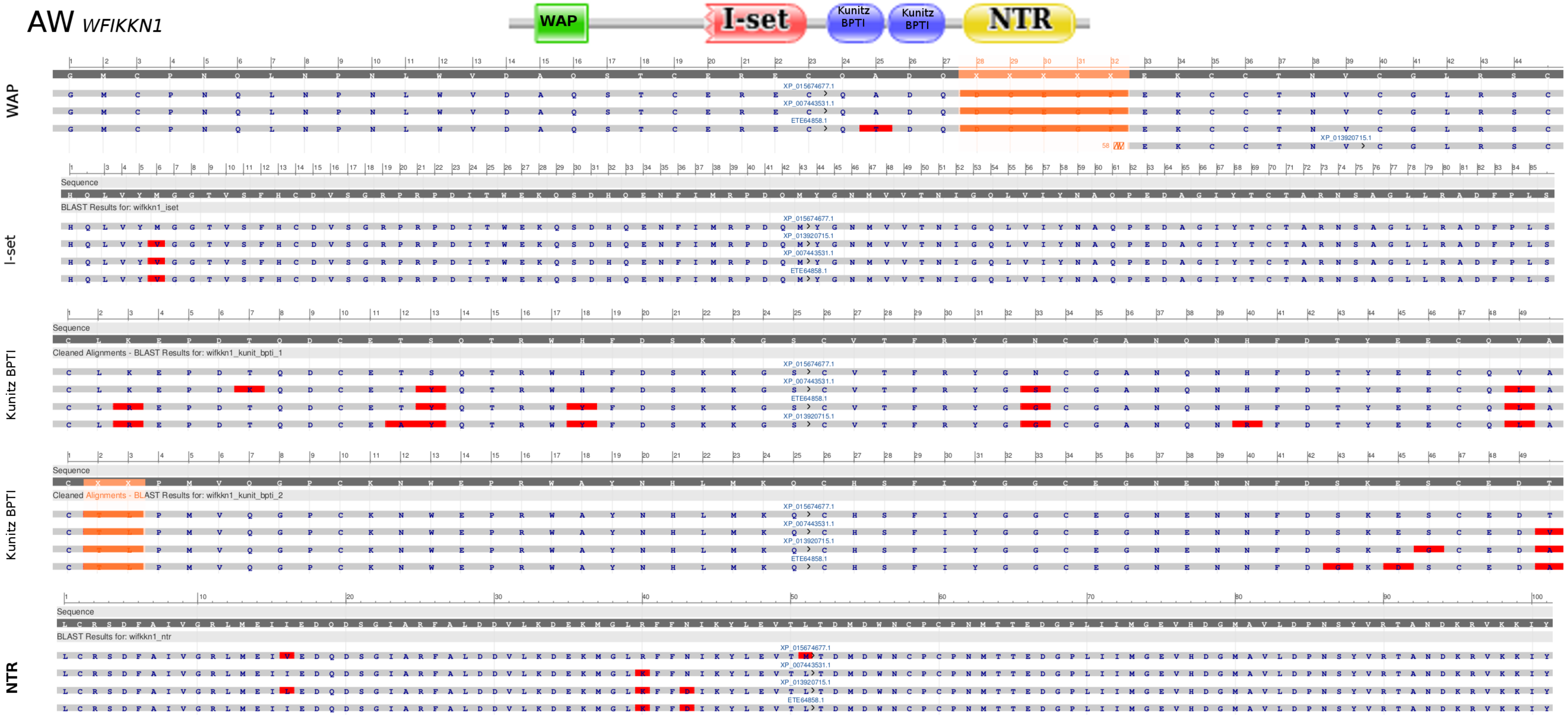

AW WFIKKN1
WAP
I-set
Kunitz BPTI
Kunitz BPTI
NTR
WAP
XP_015674677.1
XP_007443531.1
ETE64858.1
XP_013920715.1
I-set
Sequence
BLAST Results for: wifkkn1_iset
Kunitz BPTI
Sequence
Cleaned Alignments - BLAST Results for: wifkkn1_kunit_bpti_1
Kunitz BPTI
Sequence
Cleaned Alignments - BLAST Results for: wifkkn1_kunit_bpti_2
NTR
Sequence
BLAST Results for: wifkkn1_ntr

AX *WFIKKN2*

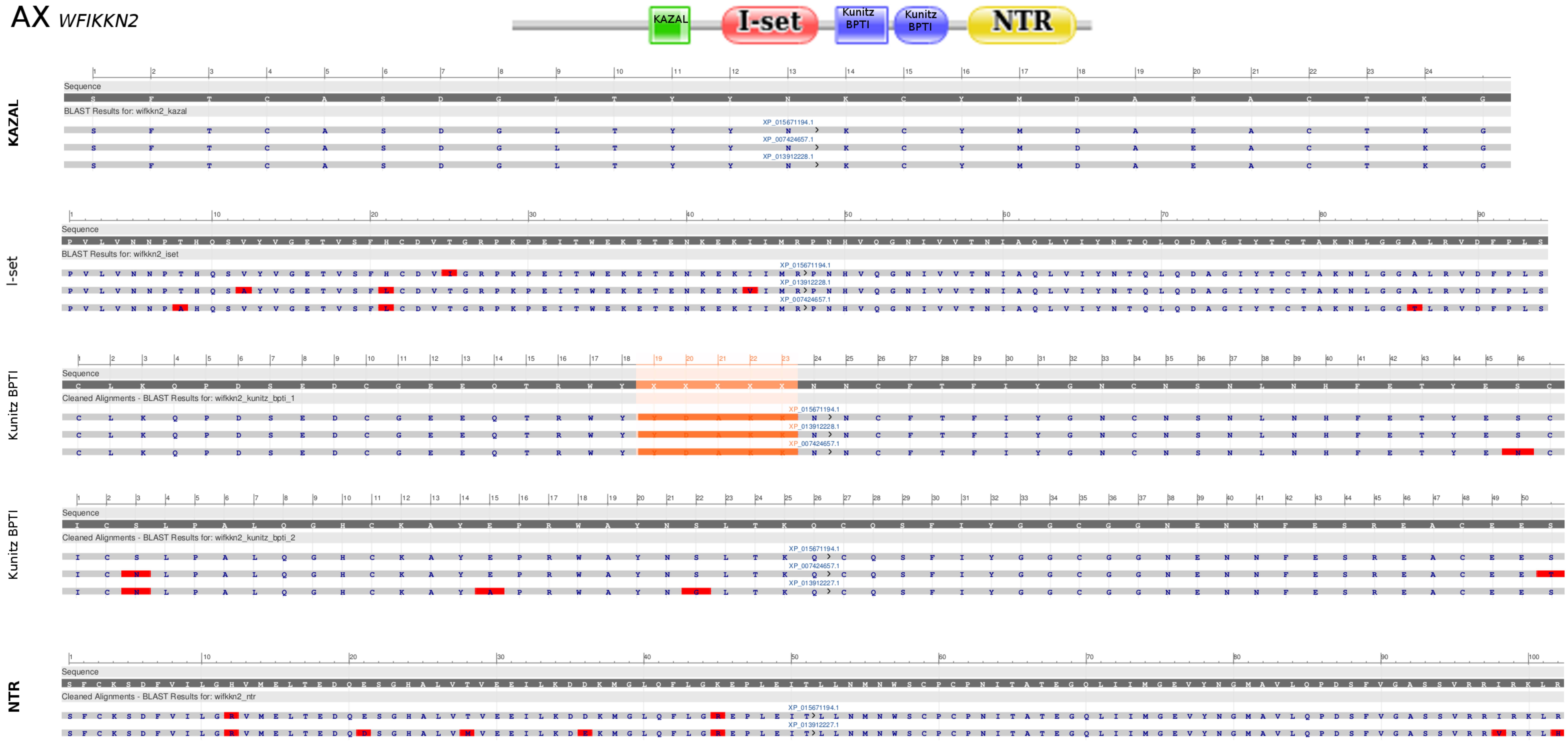

KAZAL
I-set
Kunitz BPTI
Kunitz BPTI
NTR
KAZAL
I-set
Kunitz BPTI
Kunitz BPTI
NTR
Sequence
BLAST Results for: wifkkn2_kazal
BLAST Results for: wifkkn2_iset
Cleaned Alignments - BLAST Results for: wifkkn2_kunitz_bpti_1
Cleaned Alignments - BLAST Results for: wifkkn2_kunitz_bpti_2
Cleaned Alignments - BLAST Results for: wifkkn2_ntr
