# Supplementary material for: Comparative analyses of putative toxin gene homologs from an Old World viper, Daboia russelii
Source: PeerJ. 2017 Dec 5;5:e4104. doi: 10.7717/peerj.4104 (PMC5721910; doi:10.7717/peerj.4104)
Supplement: Supplemental Information 2 [file peerj-05-4104-s002.pdf]

Fig. S7: A larger pool of sequences available from various snakes and other reptile groups (Colubridae, Boidae, Pythonidae, Acrochordidae, Lizards, Crocodiles and Testudines) were compared with the sequence of Russell's viper for putative homologs of NGF, PDGF, Kunitz BPTI, CAP and CRISP domains.

A *NGF*

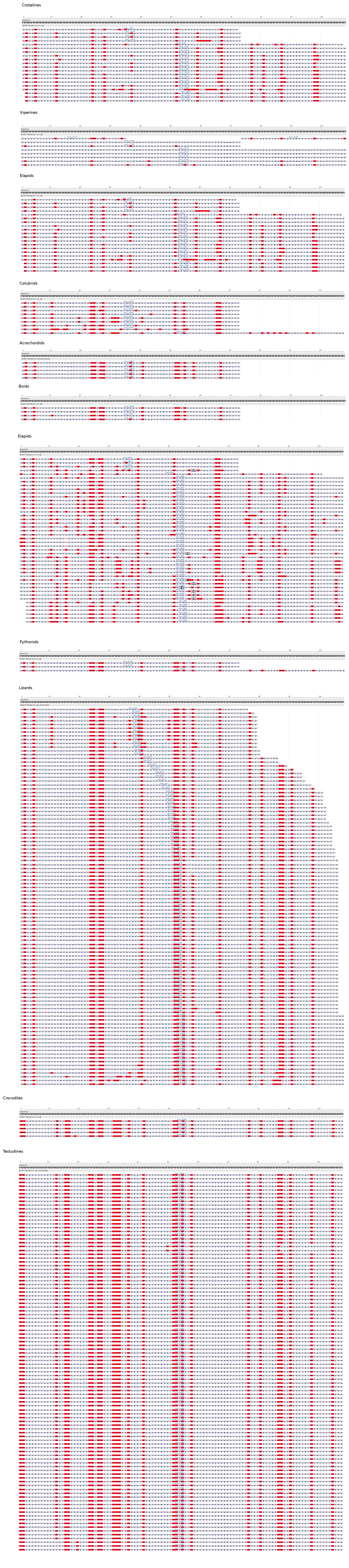

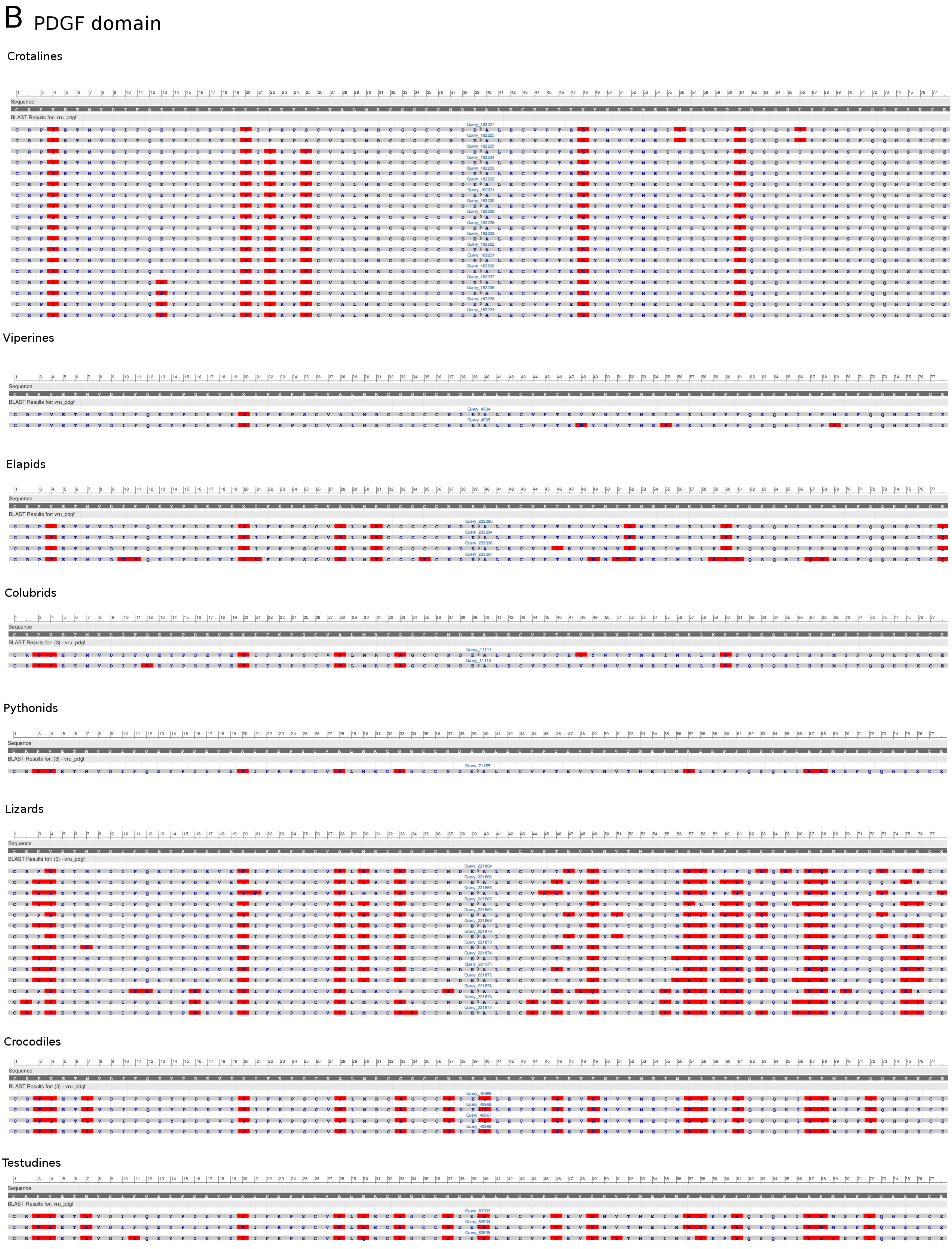

B PDGF domain
Crotalines
Viperines
Elapids
Colubrids
Pythonids
Lizards
Crocodiles
Testudines

C kunitoxin

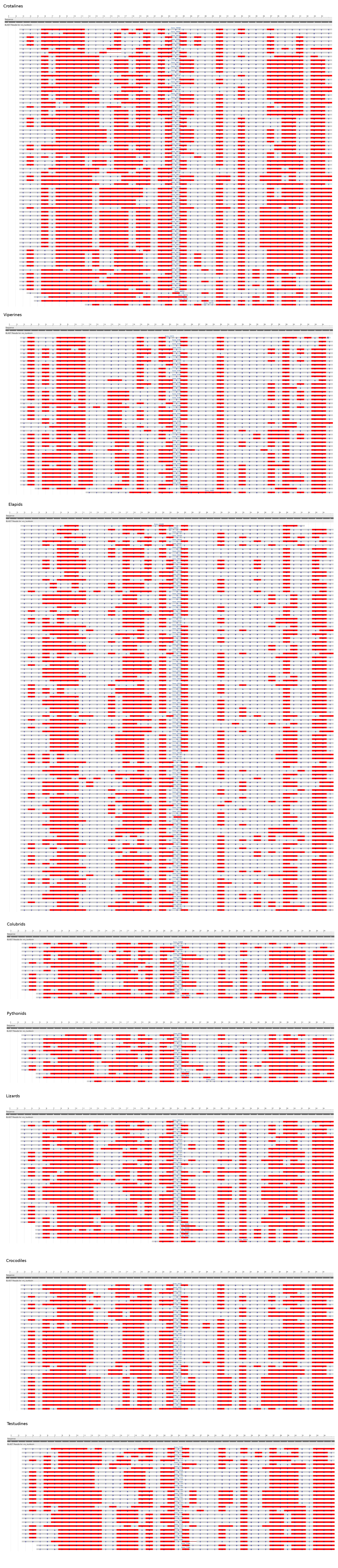

Crotalines
Viperines
Elapids
Colubrids
Pythonids
Lizards
Crocodiles
Testudines

D CAP domain

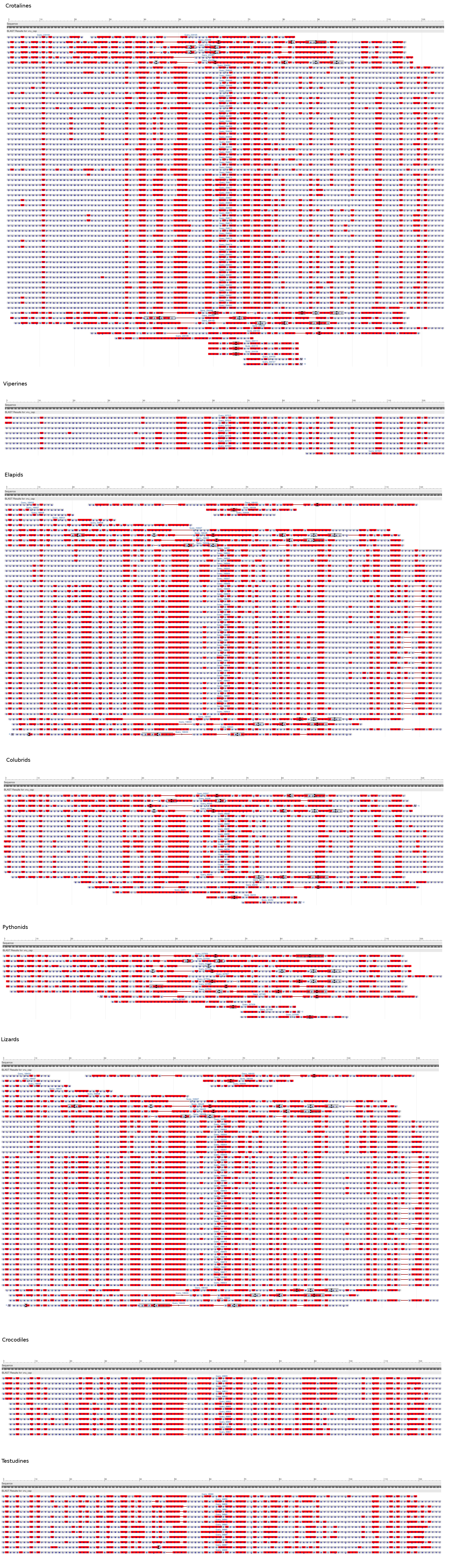
Crotalines
Viperines
Elapids
Colubrids
Pythonids
Lizards
Crocodiles
Testudines

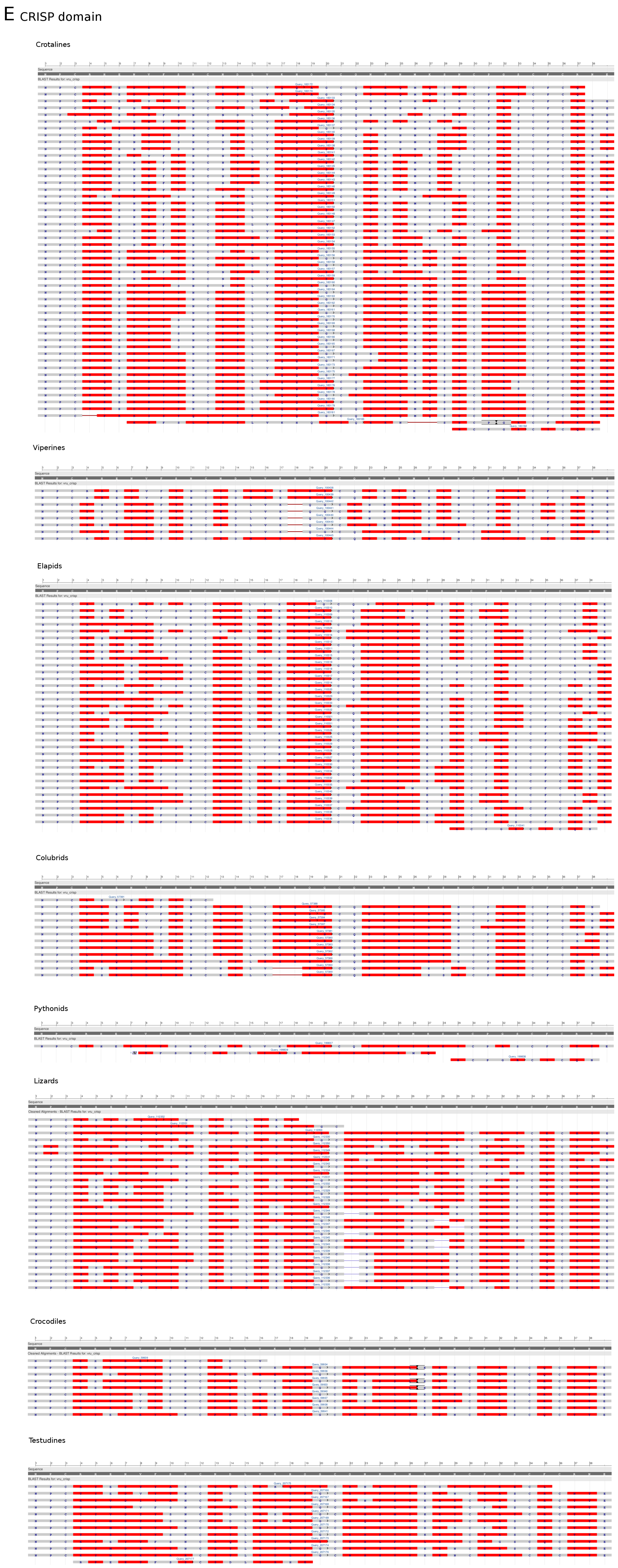
E CRISP domain
Crotalines
Viperines
Elapids
Colubrids
Pythonids
Lizards
Crocodiles
Testudines

Fig. S8: 3D protein structural model quality and function measuring parameter comparison across crotalines (CR), viperines (VP) and elapids in NGF,PDGF, Kunitz BPTI, CAP and CRISP domains. The status of the parameters being investigated using Phyre2 are indicated in the color legends on the side.

A. *NGF*

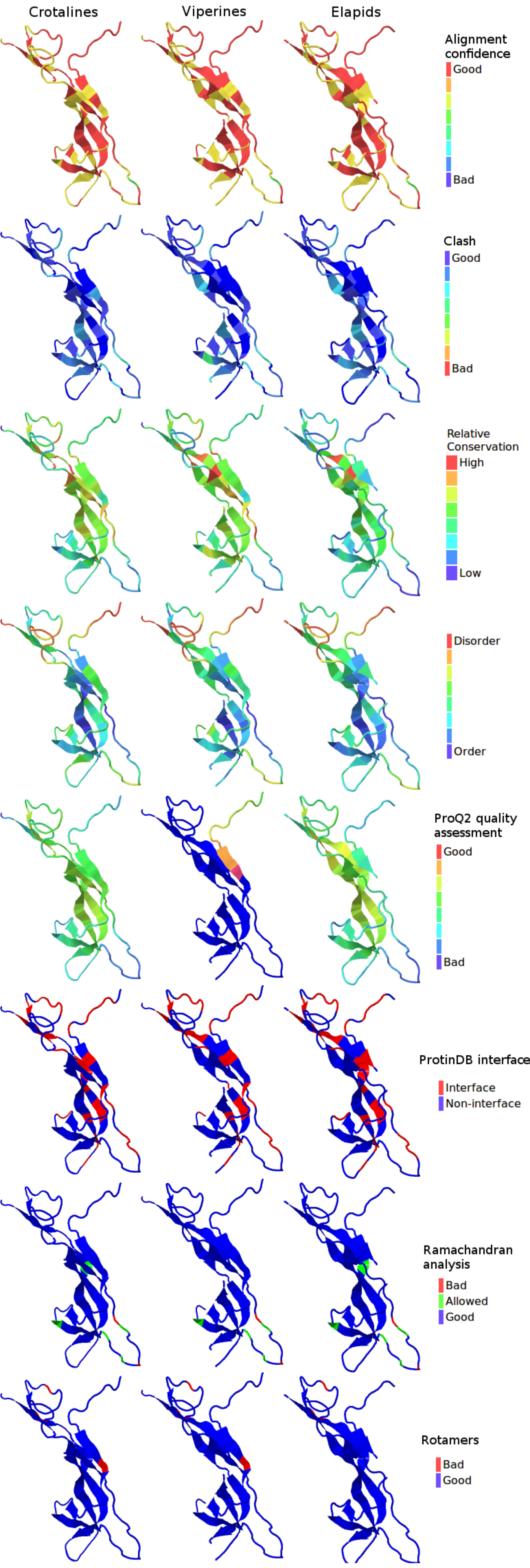

B. PDGF

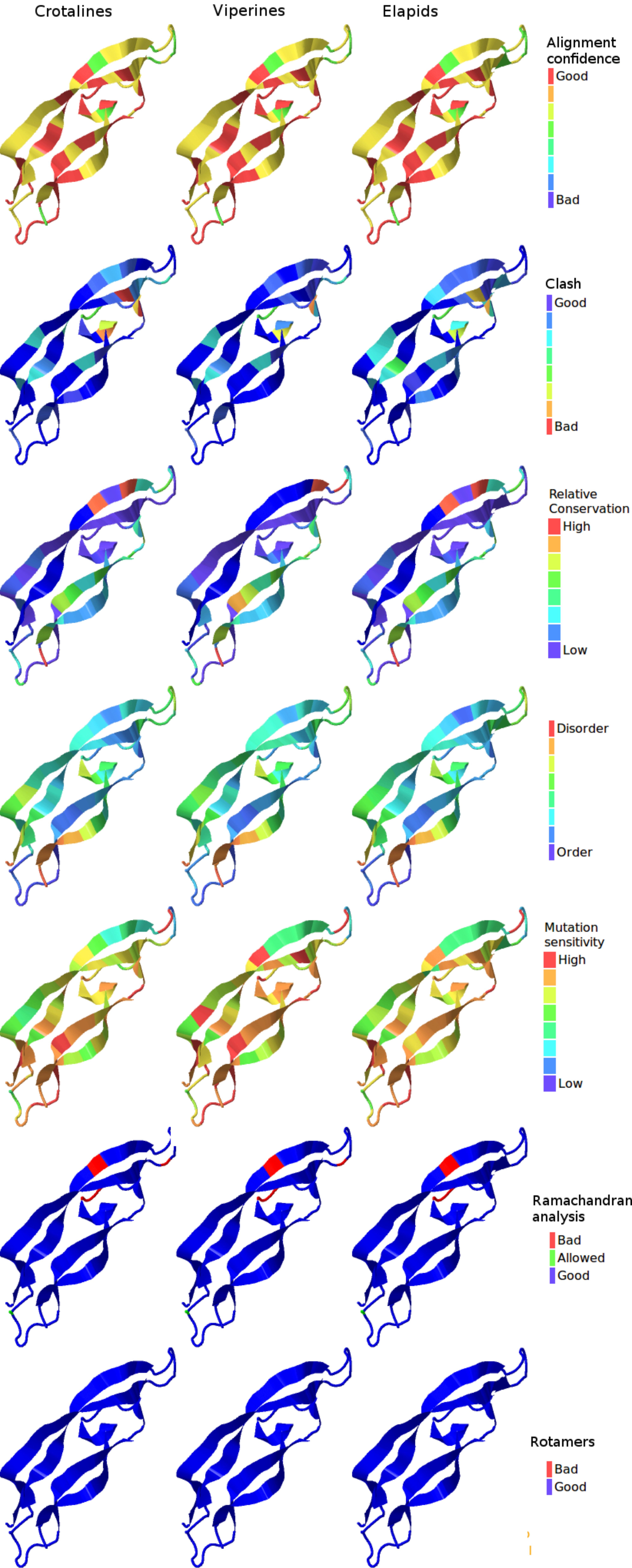

Crotalines
Viperines
Elapids
Alignment confidence
Good
Bad
Clash
Good
Bad
Relative Conservation
High
Low
Disorder
Order
Mutation sensitivity
High
Low
Ramachandran analysis
Bad
Allowed
Good
Rotamers
Bad
Good

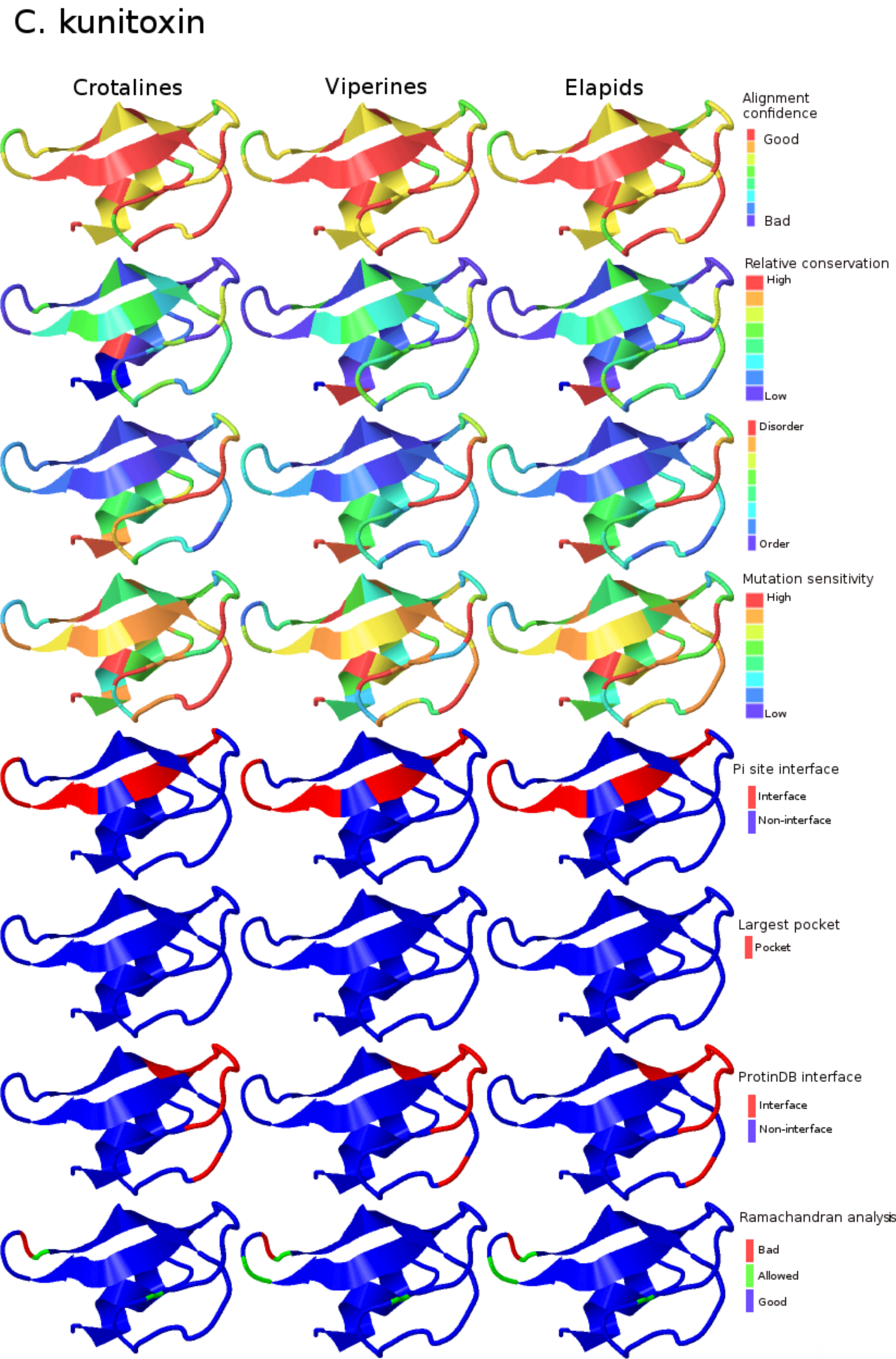
C. kunitoxin
Crotalines
Viperines
Elapids
Alignment confidence
Good
Bad
Relative conservation
High
Low
Disorder
Order
Mutation sensitivity
High
Low
Pi site interface
Interface
Non-interface
Largest pocket
Pocket
ProtinDB interface
Interface
Non-interface
Ramachandran analysis
Bad
Allowed
Good

D. CAP

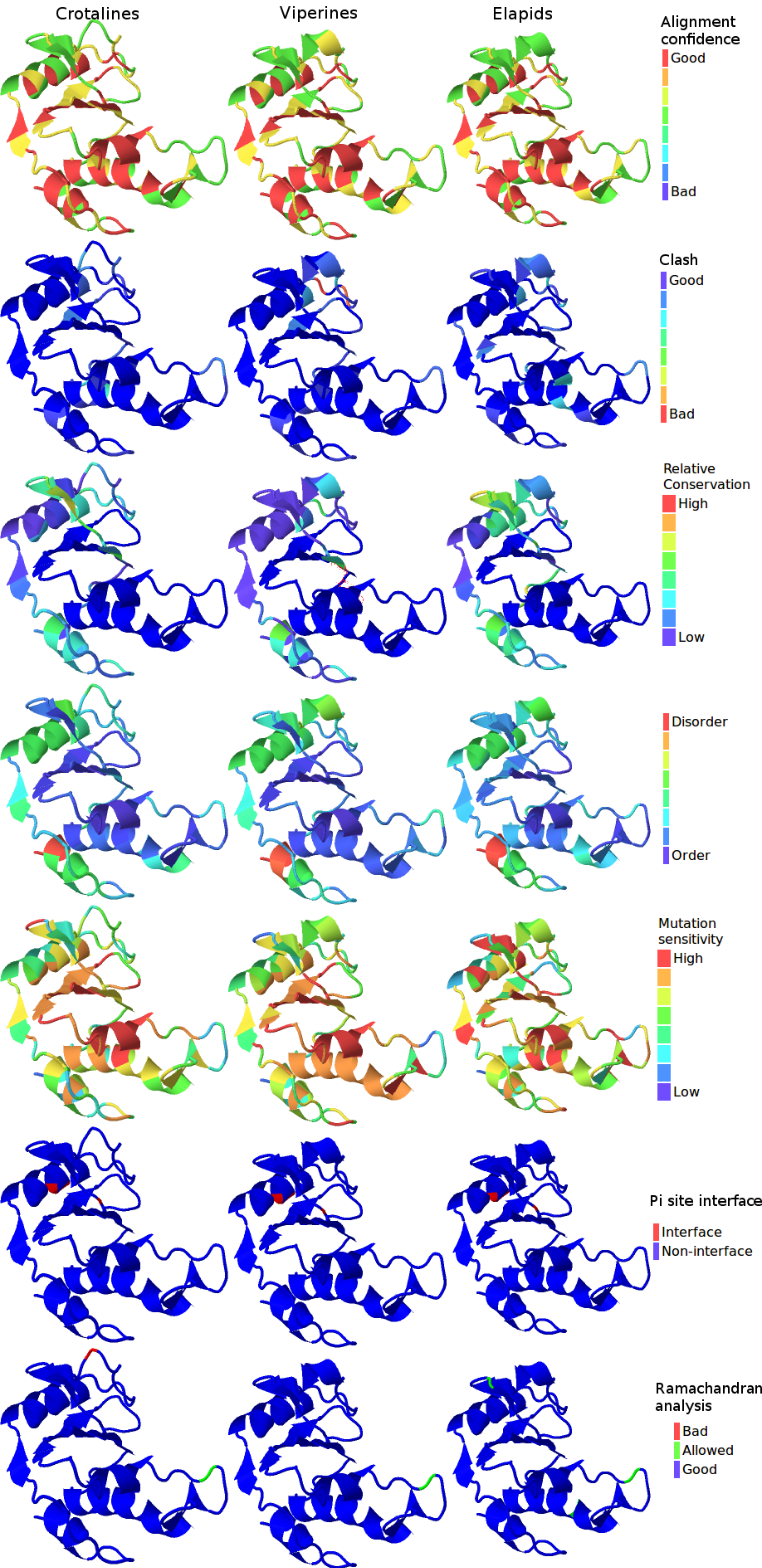

Crotalines
Viperines
Elapids
Alignment confidence
Good
Bad
Clash
Good
Bad
Relative Conservation
High
Low
Disorder
Order
Mutation sensitivity
High
Low
Pi site interface
Interface
Non-interface
Ramachandran analysis
Bad
Allowed
Good

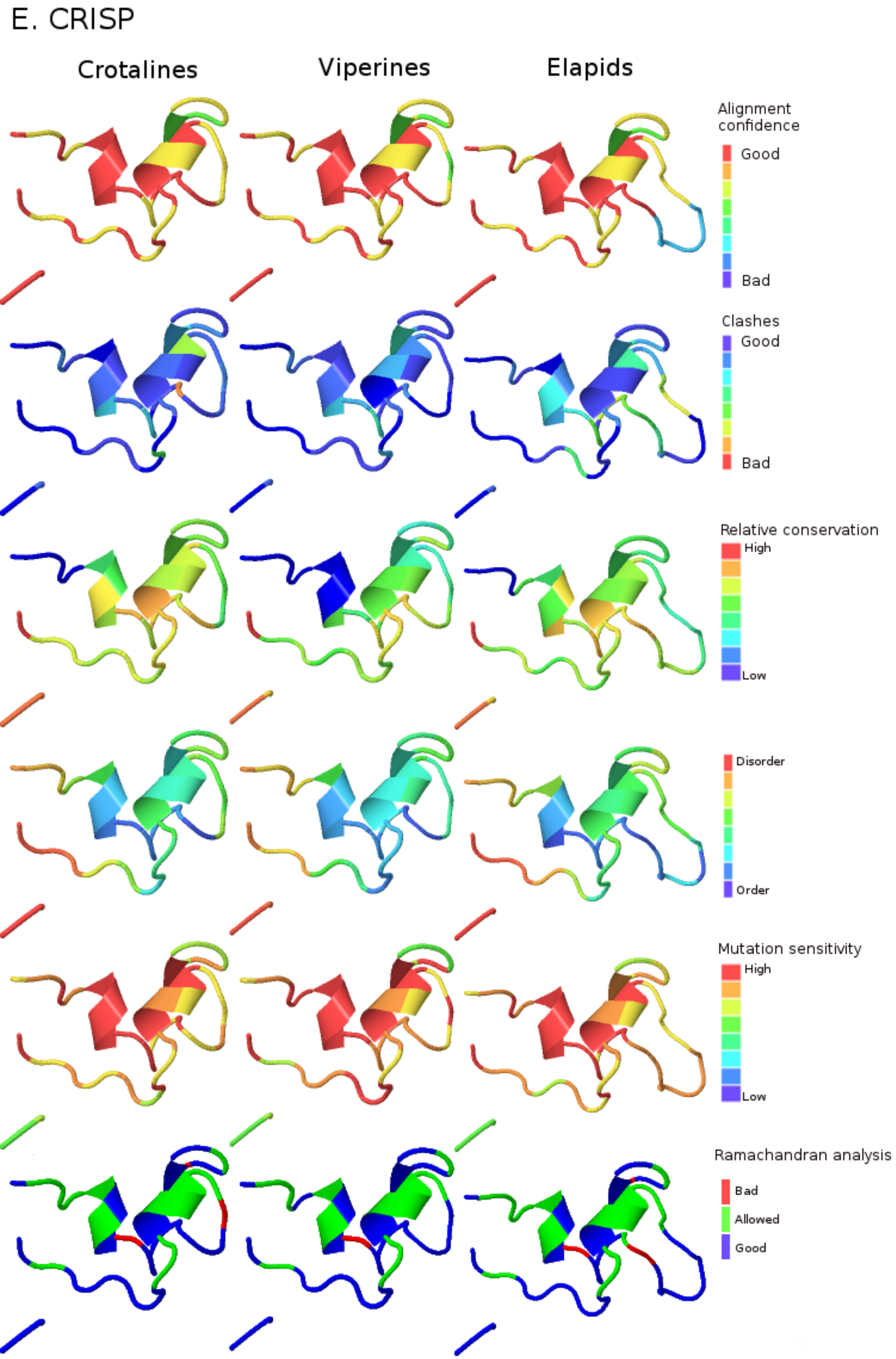
E. CRISP
Crotalines
Viperines
Elapids
Alignment confidence
Good
Bad
Clashes
Good
Bad
Relative conservation
High
Low
Disorder
Order
Mutation sensitivity
High
Low
Ramachandran analysis
Bad
Allowed
Good

Fig. S9: A comparative compositional analysis of the major venom proteins in Russell's viper (A). Data from 4 past studies were compiled together for plotting. The actual data is shown in B.

A

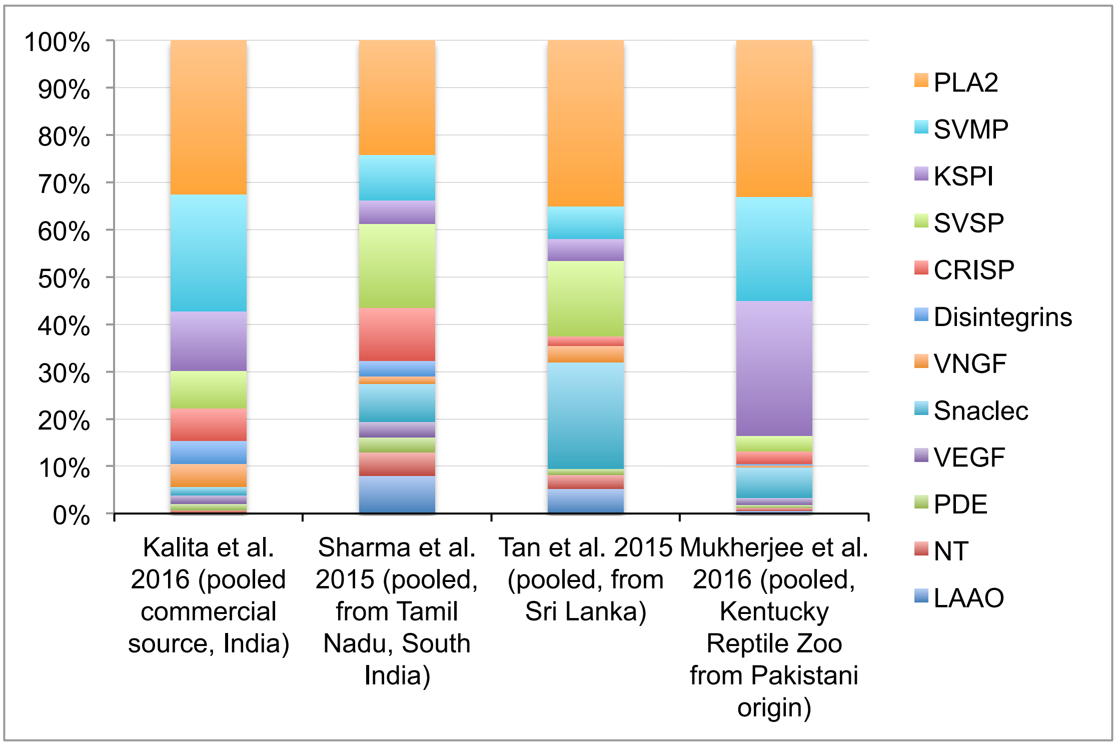

B

	Kalita et al. 2016	Sharma et al. 2015	Tan et al. 2015	Mukherjee et al. 2016
LAAO	0.3	7.9	5.2	0.6
NT	0.4	4.8	3	0.6
PDE	1.4	3.2	1.3	0.6
VEGF	1.8	3.2	0	1.5
Snaclec	1.8	7.9	22.4	6.4
VNGF	4.8	1.6	3.5	0.4
Disintegrins	4.9	3.2	0	0.4
CRISP	6.8	11	2	2.6
SVSP	8	17.5	16	3.2
KSPI	12.5	4.8	4.6	28.4
SVMP	24.8	9.5	6.9	21.8
PLA2	32.5	23.8	35	32.8
